# Chemical and biological diversity of new natural products from marine sponges: a review (2009–2018)

**DOI:** 10.1007/s42995-022-00132-3

**Published:** 2022-08-01

**Authors:** Li-Li Hong, Ya-Fang Ding, Wei Zhang, Hou-Wen Lin

**Affiliations:** 1 Research Center for Marine Drugs, State Key Laboratory of Oncogenes and Related Genes, Department of Pharmacy, Ren Ji Hospital, School of Medicine, Shanghai Jiao Tong University, Shanghai, 200127 China; 2 School of Food and Pharmacy, Zhejiang Ocean University, Zhoushan, 316000 China; 3 Centre for Marine Bioproducts Development, Flinders University, Adelaide, SA 5042 Australia

**Keywords:** Marine sponges, New compounds, Bioactivity, Marine natural products

## Abstract

Marine sponges are productive sources of bioactive secondary metabolites with over 200 new compounds isolated each year, contributing 23% of approved marine drugs so far. This review describes statistical research, structural diversity, and pharmacological activity of sponge derived new natural products from 2009 to 2018. Approximately 2762 new metabolites have been reported from 180 genera of sponges this decade, of which the main structural types are alkaloids and terpenoids, accounting for 50% of the total. More than half of new molecules showed biological activities including cytotoxic, antibacterial, antifungal, antiviral, anti-inflammatory, antioxidant, enzyme inhibition, and antimalarial activities. As summarized in this review, macrolides and peptides had higher proportions of new bioactive compounds in new compounds than other chemical classes. Every chemical class displayed cytotoxicity as the dominant activity. Alkaloids were the major contributors to antibacterial, antifungal, and antioxidant activities while steroids were primarily responsible for pest resistance activity. Alkaloids, terpenoids, and steroids displayed the most diverse biological activities. The statistic research of new compounds by published year, chemical class, sponge taxonomy, and biological activity are presented. Structural novelty and significant bioactivities of some representative compounds are highlighted. Marine sponges are rich sources of novel bioactive compounds and serve as animal hosts for microorganisms, highlighting the undisputed potential of sponges in the marine drugs research and development.

**Supplementary Information:**

The online version contains supplementary material available at 10.1007/s42995-022-00132-3.

## Introduction

Marine sponges are the oldest metazoan group with approximately 15,000 species having been described, of which 8553 species were accepted (Thomas et al. 2010; Van Soest et al. 2012). Under extreme marine environments, sponges continue to produce novel bioactive metabolites to protect them from threats of predators, competitors, and pathogens (Paul et al. 2006; Wu et al. 2021a). Their chemical arsenal encompasses terpenoids, alkaloids, polyketides, peptides, steroids, and so on. Starting with the isolation of nucleoside derivatives from sponge *Tectitethya crypta*, the discovery of sponge-derived natural products experienced a rapid growth period, followed by a stable period. Up to now, more than 18,149 new compounds have been isolated from sponges with an increasing number of over 200 new compounds isolated yearly (Carroll et al. 2021; Hu et al. 2015). Many of these molecules demonstrated diverse biological activities, such as anticancer, antibacterial, antifungal, anti-inflammatory, antiviral, antioxidant, antimalarial, and pest resistance properties (Abraham et al. 2021; Carroll et al. 2020; 2021). For this reason, sponges continue to be an attractive subject for natural product chemists based on the large number of compounds produced, the diversity of structures encountered, and the therapeutic potential of molecules.

This review summarizes sponge-derived 2762 new compounds with 1419 bioactive from 878 original research papers during 2009–2018. These new compounds in terms of published year, chemical class, sponge taxonomy, and biological activity are classified, analyzed, and evaluated. Structural novelty and excellent pharmacological activities of some representative compounds are highlighted.

## Statistical research of new compounds

**Fig. 1:**
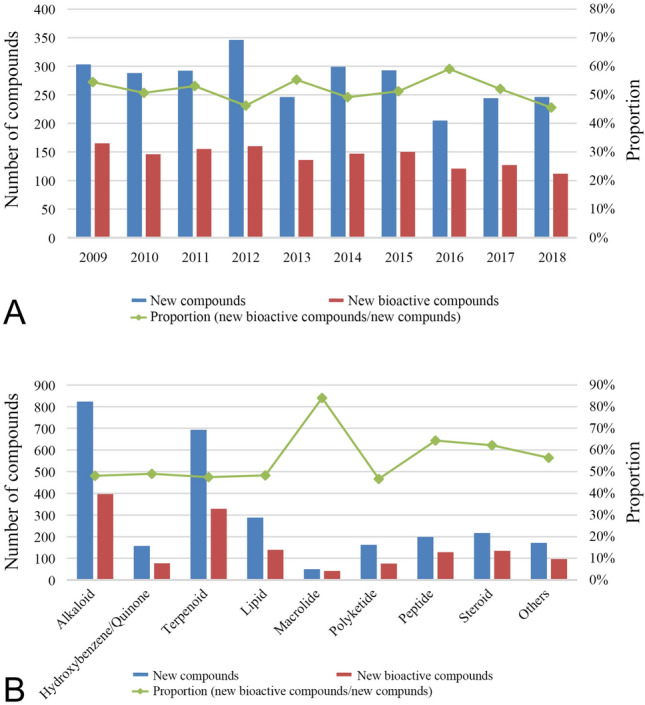
**A** Temporal trends in the number and proportion of new bioactive compounds for 2009–2018. **B** The number and proportion of new bioactive compounds in each chemical class for 2009–2018

The data are based on the literature search in the SciFinder database with marine sponge as the key word, English as the language, and the time limit of 2009–2018. Approximately 2762 new metabolites have been reported from sponges between 2009 and 2018, more than half of which showed pharmacological activity. As shown in Fig. 1A, the number of new compounds gradually decreased in a three or 4-year cycle, probably because research on MNPs from sponges gradually shifted to sponge-derived microorganisms due to increasing evidence that symbiotic microorganisms rather than sponges were likely to be the real producers of bioactive compounds (Liu et al. 2019; Zhang et al. 2017). In addition, microorganisms have the ability to reproduce indefinitely and to easily be mined genomically to obtain target metabolites (Cao and Wang 2020; Meng et al. 2021; Peng et al. 2021; Zhang et al. 2021). The proportion curve of new bioactive compounds compared to total new compounds showed that the proportion fluctuated in a small range each year. This may indicate that the rate of bioactivity screening research and discovery of new natural products was relatively stable. In addition, sampling methods, extraction and separation techniques, structure identification technology, and biological screening methods have reached a relatively mature level.

**Fig. 2:**
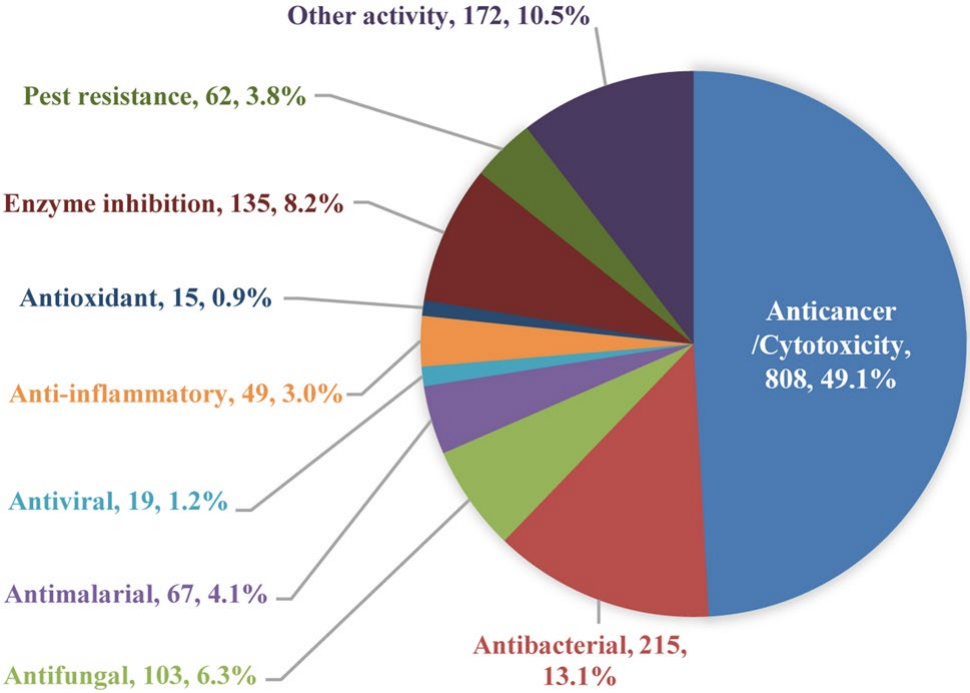
Percentage distribution of new compounds with different bioactivities for 2009–2018

Notably, the compounds are counted only once when they are analyzed by bioactivity or inactivity. However, multi-active compounds are counted multiple times when they are classified according to the following ten bioactivity groups. Figure 2 shows percentage distribution of new compounds with different bioactivities for 2009–2018. Obviously, nearly half of the new bioactive compounds showed anticancer/cytotoxic activity with the number of 808 (49.1% of the total new bioactive compounds). The main reasons of this result are likely the long term and large amount of scientific research funds supporting cancer drug discovery, big programs with the aim to discover anticancer drugs, and rapid development of effective detection technology for cytotoxicity such as MTT, XTT, and SRB assays (Hu et al. 2015). This was followed by antibacterial activity at 215 (13.1%), enzyme inhibition activity at 135 (8.2%), antifungal activity at 103 (6.3%), and antimalarial activity at 67 (4.1%). These results were consistent with the previous reviews where the two major bioactivities reported by compounds from sponges were cytotoxicity followed by antimicrobial (antifungal and antibacterial) activity (Abdelaleem et al. 2020). It is worth noting that this does not mean major bioactivities of sponge-derived compounds are cytotoxicity and antimicrobial activities. The difficulty of the biological screening model may affect this result to a certain extent. For instance, viruses are underrepresented as targets in pharmacological screening efforts due to the requirement of biochemical assay counter screens and inherent complexity of cell-based assays of viruses, making them expensive and time consuming (O’rourke et al. 2018).

The new compounds are divided into nine chemical classes including alkaloids, terpenoids, hydroxybenzene/quinones, lipids, macrolides, polyketides, peptides, steroids, and others. However, it is noteworthy that macrolides and steroids are often classified as polyketides and lipids, respectively. Here we list macrolides and steroids separately because of their significant pharmacological activity and large quantities, respectively. Figure 1B shows the number and proportion of new bioactive compounds in each chemical class. 823 and 693 new compounds belonged to alkaloids and terpenoids, respectively, adding up to more than half of the total. Similarly, these two classes contributed 50% of all new bioactive compounds. Although the number of bioactive alkaloids and terpenoids was the largest, the highest proportion of bioactives belonged to macrolides with 84.0% followed by peptides with 64.3%. Two recent reviews summarized marine-derived macrolides with therapeutic potential, which displayed a wide range of bioactivities including cytotoxic, antifungal, antiviral, antibacterial, antimitotic, and other activities (Wu et al. 2021b; Zhang et al. 2021). Peptides were promising drug candidates due to their reduced size, stability, low immunogenicity, and diversity of bioactivities including anti-proliferative, antiviral, anti-coagulant, antioxidant, antiobesity, antidiabetic, anti-hypertensive, and calcium-binding activities (Gogineni and Hamann 2018; Hu et al. 2015). This was then followed by steroids with 62.6%, hydroxybenzene/quinones with 49.0%, alkaloids with 48.1%, and terpenoids with 47.5%.

**Fig. 3:**
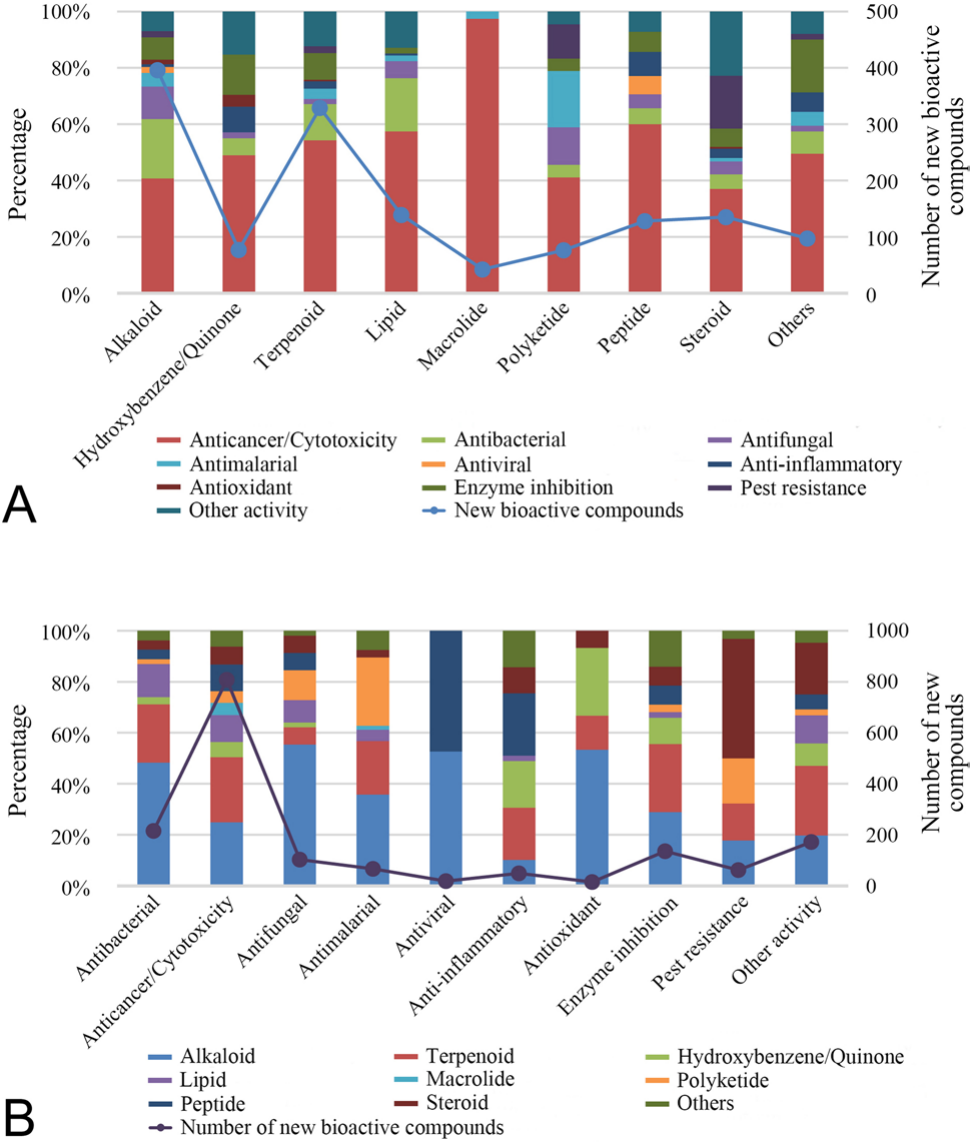
**A** Percentage distribution of new compounds with different bioactivities in each chemical class for 2009–2018. **B** Percentage distribution of new compounds with different chemical classes in each bioactivity for 2009–2018

Figure 3A shows the proportion of different activities in each category of chemical compounds for 2009–2018. The analyzed data shows that bioactivity distribution is slightly affected by chemical structures. All chemical groups displayed cytotoxicity as the dominant activity with the proportion ranging from 37.0% to 97.5%. Especially for macrolides, cytotoxic compounds accounted for 97.5% of the total active compounds, highlighting that they encompass many potential antitumor drug leads. Regardless of cytotoxic property, alkaloids, terpenoids, and lipids mainly showed antibacterial activity, while hydroxybenzene/quinones, polyketides, and steroids displayed enzyme inhibition, antimalarial, and pest resistance property as major activities, respectively. In addition, the distribution of all types of activities but cytotoxicity displayed by peptides was relatively average.

As shown in Fig. 3B, the analyzed data shows that alkaloids, terpenoids, lipids, and peptides were responsible for cytotoxic activity. The major contributors to antibacterial activity were alkaloids, terpenoids, and lipids. The most promising antifungal agents from sponges appear to be alkaloids and polyketides. A certain number of alkaloids, terpenoids, and peptides exhibited antimalarial activity. Only alkaloids and peptides were reported from sponges this decade to possess antiviral activity. The main anti-inflammatory metabolites were terpenoids, hydroxybenzenes/quinones, and peptides. Alkaloids and hydroxybenzenes/quinones were the primary antioxidant constituents of the sponges. Alkaloids and terpenoids were responsible for enzyme inhibition activity while steroids, polyketides, alkaloids, and terpenoids contributed to pest resistance activity. Alkaloids, terpenoids, and steroids displayed the most diverse biological activities.

**Fig. 4:**
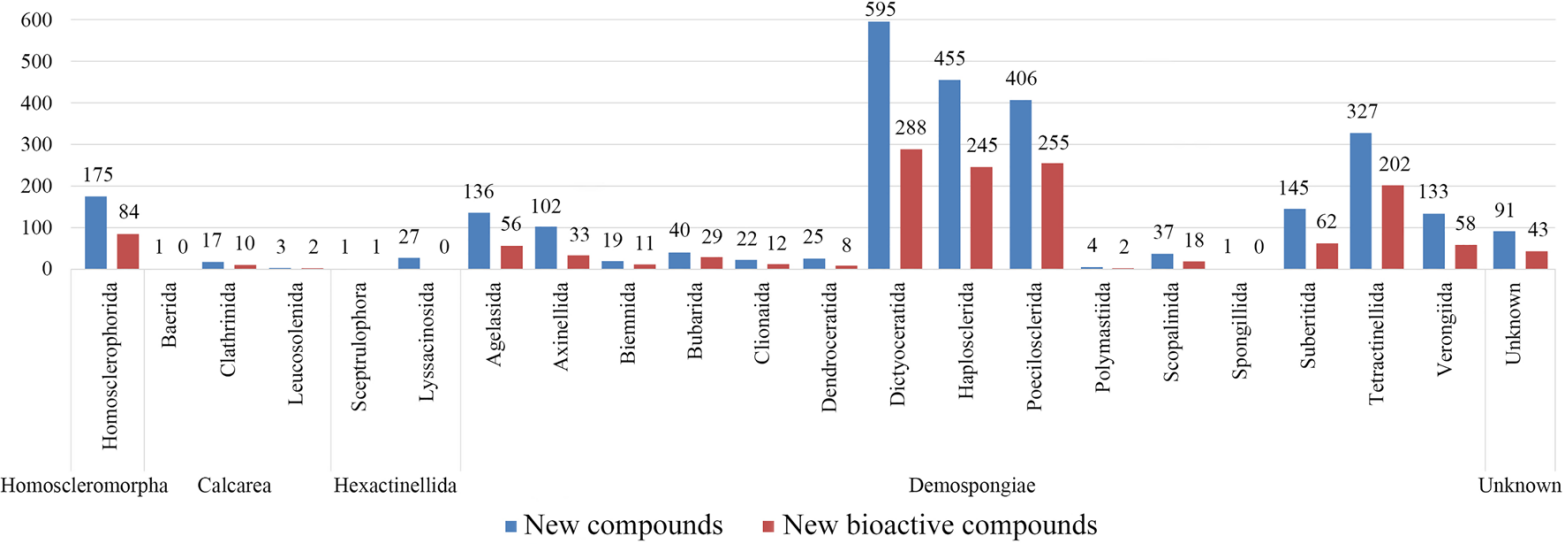
Number of new compounds isolated from different orders for 2009–2018

The World Porifera Database is utilized by the taxonomic classification of the sponges mentioned in the original research papers. According to the world porifera database, sponges are composed of 5 classes and 39 orders. As shown in Fig. 4, during 2009–2018, about 4 classes and 21 orders were studied for discovery of new metabolites, with the class Demospongiae being the most prolific producer with 2447 new compounds reported. Orders Dictyoceratida, Haplosclerida, Poecilosclerida, and Tetractinellida from the class Demospongiae were the most productive orders, giving 595, 455, 406, and 327 new compounds, respectively.

## New bioactive compounds from sponges

**Fig. 5:**
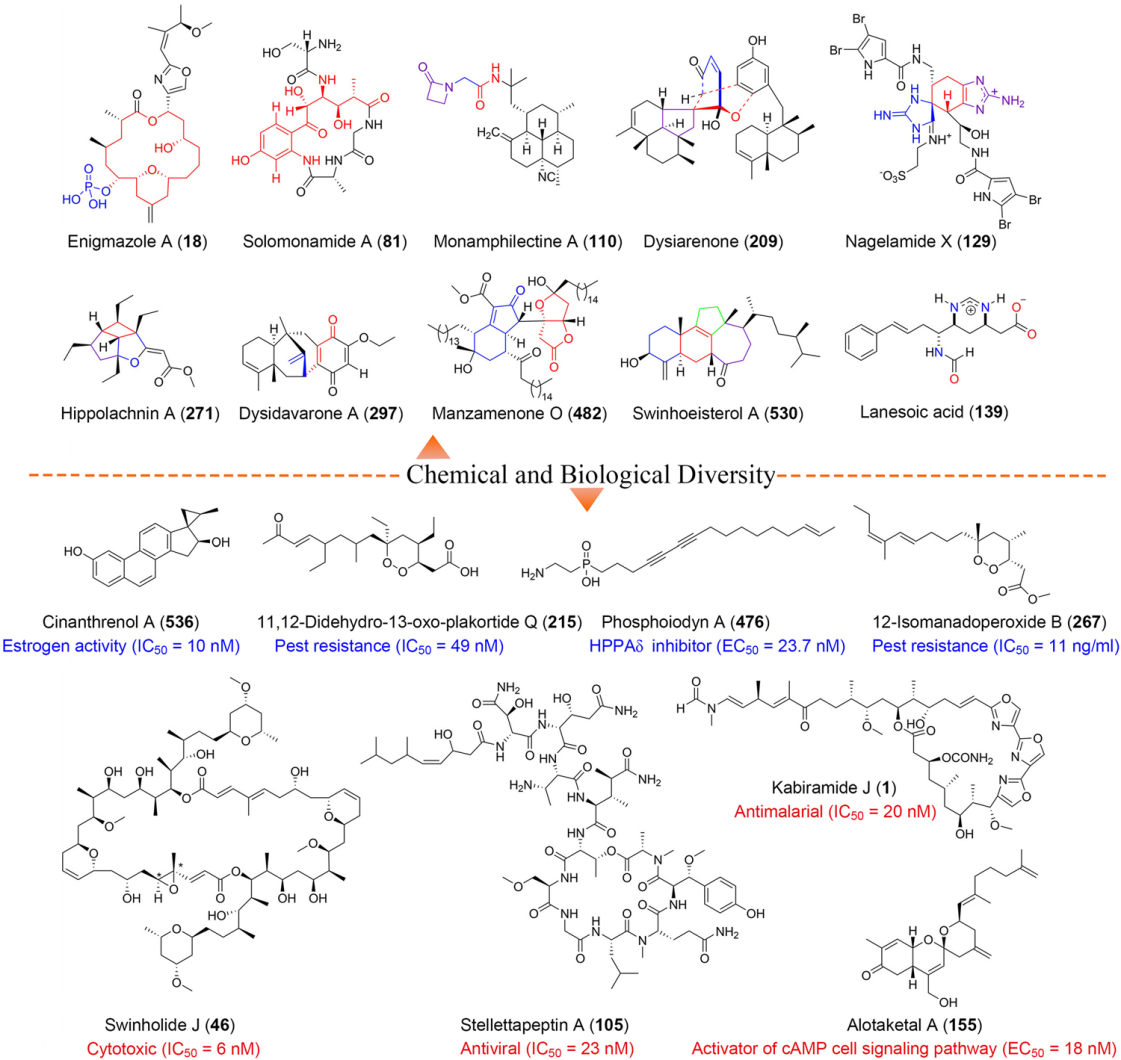
Structures of selected representative compounds isolated from marine sponges for 2009–2018

Approximately 2762 new metabolites have been reported from sponges for 2009–2018, some of which possessed novel skeleton and showed distinguishing pharmacological activity. Herein, structural novelty and excellent bioactivities of 553 representative compounds are highlighted (Fig. 5).

### Macrolides

Kabiramides J and K (**1** and **2**) were trisoxazole macrolides isolated from *Pachastrissa nux*. Both displayed significant antimalarial (IC_50_ = 20 and 70 nmol/L) and cytotoxic (IC_50_ = 0.31 and 0.39 μmol/L) activities (Sirirak et al. 2011). Examination of *P. nux* resulted in the isolation of one further antimalarial trisoxazole macrolide, kabiramide L (**3**) (Sirirak et al. 2013). Two further trisoxazole macrolides, miuramides A (**4**) and B (**5**), were isolated from *Mycale* sp., both with strong cytotoxicity (3Y1 cells, IC_50_ = 7 nmol/L) (Suo et al. 2018b). Zampanolides B–E (**6**–**9**) had been reported from *Cacospongia mycofijiensis*. Zampanolides B–D (**6**–**8**) exhibited strong cytotoxicity against the HL-60 cell line, were antimitotic, and induced tubulin polymerization with zampanolide E (**9**) being much less active due to saturation at C-8/C-9 (Taufa et al. 2018). A novel macrolide, callyspongiolide (**10**), was isolated from the marine sponge *Callyspongia* sp., which featured a conjugated structurally unprecedented diene-ynic side chain ending at a brominated benzene ring. Callyspongiolide (**10**) exhibited strong inhibition of human Jurkat J16 T and Ramos B lymphocytes (IC_50_ = 70 and 60 nmol/L) (Pham et al. 2014). A *Candidaspongia* sp. yielded two inseparable mixture of isomers, precandidaspongiolides A/B (**11**/**12**) and candidaspongiolides A/B (**13**/**14**), which showed nanomolar activity to various cell lines with IC_50_ values ranging from 1.6 to 17.9 nmol/L (Whitson et al. 2011). Additional research on another *Candidaspongia* sp. yielded two new macrolides **15** and **16** that displayed potent cytotoxicity (IC_50_ = 4.7 and 19 ng/ml) (Trianto et al. 2011). The chondropsin-type macrolide poecillastrin H (**17**), obtained from *Characella* sp., was strongly active against 3Y1 cells (IC_50_ = 4.1 nmol/L) (Suo et al. 2018a). Investigation of *Cinachyrella enigmatica* yielded three novel phosphate-containing macrolides, enigmazole A (**18**), 15-O-methylenigmazole A (**19**), and 13-hydroxy-15-O-methylenigmazole A (**20**). The enigmazoles were unprecedented 18-membered macrolide with an embedded 2,6-disubstituted 4-methylenetetrahydropyran moiety and a disubstituted oxazole attachment to the macrocyclic ring. In the NCI 60-cell antitumor assay, enigmazole A (**18**) exhibited significant cytotoxicity with a mean GI_50_ of 1.7 μmol/L (Oku et al. 2010). *Fascaplysinopsis* sp. was the source of a novel cytotoxic nitrogenous bismacrolide, tausalarin C (**21**) (Bishara et al. 2009). *Fascaplysinopsis* sp. gave seven new nitrogenous macrolides, salarins D–J (**22**–**28**), some of which displayed cytotoxicity against K562 and UT-7 human leukemia cells (Bishara et al. 2010). A rare polyketide-derived macrolide, leiodermatolide (**29**), was isolated from a *Leiodermatium* sp. and exhibited potent and selective antimitotic activity (IC_50_ < 10 nmol/L) against a range of human cancer cell lines by inducing G2/M cell cycle arrest (Paterson et al. 2011). Two further analogues, leiodermatolides B (**30**) and C (**31**), were isolated from *Leiodermatium* sp., both of which were cytotoxic to AsPC-1 cells with an IC_50_ of 43 nmol/L and 3.7 μmol/L, respectively (Wright et al. 2017). A *Lissodendoryx* sp. produced four new cyctoxic halichondrins **32**–**35** (Hickford et al. 2009). NMR-directed isolation from *Mycale hentscheli* led to the peloruside B (**36**) with potent antitumor activity, which promoted microtubule polymerization and arrested cells in the G2/M phase of mitosis as does paclitaxel (Singh et al. 2010). Further chemical investigations on *M. hentscheli* yielded pelorusides C (**37**) and D (**38**), both of which were cytotoxic against the HL-60 cell line with IC_50_ values of 221 nmol/L and 2 μmol/L, respectively (Singh et al. 2011). An additional peloruside E (**39**), isolated from *M. hentscheli*, was cytotoxic against HL-6 cells and polymerized purified tubulin (Hong et al. 2018). *Pipestela candelabra* gave pipestelides A–C (**40**–**42**) with pipestelide A (**40**) being more cytotoxic to the KB Cell Line (IC_50_ = 0.1 μmol/L) (Sorres et al. 2012). Poecillastrins E–G (**43**–**45**) were isolated from *Poecillastra* sp. and had potent cytotoxicity against rat embryonic fibroblast 3Y1 cells with the IC_50_ values of 6.7, 1.2, and 5.0 ng/ml, respectively (Irie et al. 2018). *Theonella swinhoei* yielded swinholide J (**46**), strongly cytotoxic to KB cells (IC_50_ = 6 nmol/L) (De Marino et al. 2011a). Additional research on *T. swinhoei* obtained the new dimeric macrolides isoswinholide B (**47**) and swinholide K (**48**). Both compounds showed cytotoxicity to HepG2 cells with IC_50_ values of 1.5 μmol/L and 15 nmol/L, respectively (Sinisi et al. 2013). The structures of compounds **1**–**48** are shown as supplementary Fig. S1.

### Peptides

Chemical investigation of *Citronia astra* gave citronamides A (**49**) and B (**50**) with citronamide A (**49**) being moderately active against *Saccharomyces cerevisiae* (Carroll et al. 2009). Yaku’amides A (**51**) and B (**52**) were obtained from *Ceratopsion* sp., both of which displayed strong cytotoxic activity against P388 cells with IC_50_ values of 14 and 4 ng/ml, respectively (Ueoka et al. 2010). Investigation of *Ecionemia acervus* yielded a novel class of cyclic depsiundecapeptides, stellatolides A–G (**53**–**59**), containing various nonnatural amino acids. All but stellatolide G (**59**) exhibited significant cytotoxicity towards A-549, HT-29, and MDA-MB-231 cell lines with GI_50_ values of 0.08–2.7 μmol/L (Martin et al. 2014). Lipodiscamides A–C (**60**–**62**) from *Discodermia kiiensis* were the first example of lipopeptides bearing 4S-hydroxy-trans-2-enoate and noncanonical amino acids, E-dehydronorvaline (Denor), D-citrulline (Cit), and L-3-ureidoalanine (Uda). All three compounds showed weak to moderate cytotoxicity against P388 and HeLa cells (Tan et al. 2014). Examination of *T. swinhoei* revealed a mixture of nazumazoles A–C (**63**–**65**) as an inhibitor of P388 cells (IC_50_ = 0.83 μmol/L), which featured one residue each of alanine-derived oxazole and α-keto-β-amino acid residue (Fukuhara et al. 2015). Further investigation of *T. swinhoei* yielded nazumazoles D–F (**66**–**68**) that were inhibitors of proteases with IC_50_ values of 2, 3, and 10 μmol/L, respectively (Fukuhara et al. 2016). Three additional protease inhibitors, cyclotheonellazoles A–C (**69**–**71**), were obtained from *Theonella* aff.* Swinhoei* (Issac et al. 2017). Perthamides C–K (**72**–**80**) were also sourced from *T. swinhoei*. Perthamides C (**72**), D (**73**), H (**77**), I (**78**), and K (**80**) reduced carrageenan-induced paw oedema both in the early and in the late phases while perthamides C (**72**) and E (**74**) inhibited TNF-a and IL-8 release (Festa et al. 2009, 2011b, 2012a). Two novel anti-inflammatory cyclopeptides, solomonamides A (**81**) and B (**82**), were isolated from the marine sponge *T. swinhoei* (Festa et al. 2011c). *Characella pachastrelloides* gave characellides A–D (**83**–**86**), four rare lipoglycotripeptides which contained unprecedented structural features including a core tripeptide (O-Me-Tyr-Asp-Thr) and long unusual alkyl chains and sugar units connected to the terminal threonine (Afoullouss et al. 2019). Two different species of *Theonella* sp. yielded a new sulfated cyclic depsipeptide, mutremdamide A (**87**), and six new linear or cyclic highly N-methylated peptides, koshikamides C–H (**88**–**94**), of which only koshikamide H (**94**) displayed cytotoxicity toward HCT-116 cells (IC_50_ = 10 μmol/L). In addition, cyclic koshikamides F (**92**) and H (**94**) inhibited HIV-1 entry with IC_50_ values of 2.3 and 5.5 μmol/L while their linear counterparts were inactive (Plaza et al. 2010). *Siliquariaspongia mirabilis* was the source of six new depsipeptides, celebesides A–C (**95**–**97**) and theopapuamides B–D (**98**–**100**). Celebesides A–C (**95**–**97**) exhibited cytotoxic and antifungal activities, of which celebeside A (**95**) also displayed inhibition of HIV-1 in a neutralization assay (Plaza et al. 2009). *Stelletta clavosa* produced four new depsipeptides, mirabamides E–H (**101**–**104**), which neutralized HIV-1 with IC_50_ values of 121, 62, 68, and 41 nmol/L, respectively (Lu et al. 2011). Investigation of another *Stelletta* sp. gave two cyclic depsipeptides, stellettapeptins A (**105**) and B (**106**), both of which potently inhibited HIV-1_RF_ infection in human T-lymphoblastoid cells with EC_50_ values of 23 and 27 nmol/L, respectively (Shin et al. 2015). A *Petrosia* sp. produced three new structurally related depsipeptides, halicylindramides F–H (**107**–**109**), of which halicylindramide F (**107**) showed antagonistic activities towards hFXR (IC_50_ = 6.0 μmol/L) (Hahn et al. 2016). The structures of compounds **49**–**109** are shown as supplementary Fig. S2.

### Alkaloids

Monamphilectine A (**110**) was a diterpenoid *β*-lactam alkaloid isolated from *Hymeniacidon* sp. and displayed potent antimalarial activity with an IC_50_ value of 0.60 μmol/L (Aviles and Rodriguez 2010). Of the baculiferins A–O (**111**–**115**) isolated from *Iotrochota baculifera*, baculiferins C (**113**), E–H (**115**–**118**), and K–N (**121**–**124**) were potently active against the HIV-1 IIIB virus (Fan et al. 2010). Bioassay-guided fractionation of an antimalarial extract from *Plakortis lita* yielded thiazine-derived alkaloids, thiaplakortones A–D (**125**–**128**). All compounds displayed significant antimalarial activity (IC_50_ < 651 nmol/L) (Davis et al. 2013). Nagelamides X–Z (**129**–**131**) were dimeric bromopyrrole alkaloids from *Agelas* sp., all with some degree of antimicrobial activity. Nagelamides X (**129**) and Y (**130**) possessed a new carbon skeleton including aminoimidazolidine and spiro-bonded tetrahydrobenzaminoimidazole moieties (Tanaka et al. 2013b). Another *Agelas* sp. gave two additional unprecedent dimeric bromopyrrole alkaloids with antibacterial activity, agelamadins A (**132**) and B (**133**), which possessed agelastatin-like tetracyclic and oroidin-like linear moieties (Kusama et al. 2014a). Further investigation of *Agela* sp. yielded additional agelamadins C–E (**134**–**136**), all of which were unusual 3-hydroxykynurenine/oroidin hybrids connected through a dihydro-1,4-oxazine moiety (Kusama et al. 2014b). HPLC-UV-ELSD-MS-directed fractionation of the anti-parasitic extract of *Monanchora arbuscula* gave six new guanidine and pyrimidine alkaloids, of which monalidine A (**137**) was active against against *Trypanosoma cruzi* and *Leishmania infantum* (Santos et al. 2015b). *Fascaplysinopsis reticulata* was the source of a pair of unusual bisheterocyclic quinolineimidazole alkaloids, (+)- and (−)-spiroreticulatine (**138**). The racemate and both enantiomers were significantly active against IL-2 production (Wang et al. 2015a). Lanesoic acid (**139**) was a new zwitterionic alkaloid featuring an unusual 1,4,5,6-tetrahydropyrimidine cation from *Theonella* sp. and displayed selective cytotoxic activity against pancreas tumor cells (Rodríguez et al. 2016). Examination of *Agelas mauritiana* revealed five new diterpene alkaloids with (+)-agelasine B (**140**) exhibiting inhibition of several cancer cell lines (IC_50_ = 4.49–14.07 μmol/L) and antibacterial activities against five MRSA clinical isolates (MIC_90_ = 1–8 μg/ml) (Hong et al. 2017a). Lissodendoric acids A (**141**) and B (**142**) were manzamine-related alkaloids from *Lissodendoryx florida*, both with potent capability to decrease the reactive oxygen production and somewhat increase the survival of these cells upon treatment with 6-hydroxydopamine (Lyakhova et al. 2017). A two-sponge association (*Jaspis* sp. and *Bubaris* sp.) yielded two new bromotyrosine derivatives, anomoian B (**143**) and aplyzanzine B (**144**). Both compounds showed moderate cytotoxic activity against several cancer cell lines via induction of apoptosis, which was mediated neither by the generation of reactive oxygen species nor by the inhibition of histone deacetylases in these cell lines (Tarazona et al. 2017). UPLC-qTOF-MS-based fractionation of *Geodia barretti* led to three new bromoindole alkaloids, geobarrettins A–C (**145**–**147**). Both **146** and **147** reduced IL-12p40 production by DCs and DCs treated with **146** and **147** inhibited IFN-γ secretion by co-cultured T cells, consequently reducing Th1 responses (Di et al. 2018). Two further bromopyrrole alkaloids, dioxysceptrin (**148**) and ageleste C (**149**), came from *Agelas kosrae*, of which dioxysceptrin (**148**) moderately exhibited anti-angiogenic activity as a mixture of α-amido epimers while ageleste C (**149**) inhibited isocitrate lyase activities (Kwon et al. 2018). *Leucetta chagosensis* produced five new imidazole derivatives, among which leuchagodine B (**150**) and bis(pyronaamidine)zinc (**151**) significantly inhibited the LPS-induced production of IL-6 in the human acute monocytic leukemia cell line THP-1 (Tang et al. 2018). The structures of compounds **110**–**151** are shown as supplementary Fig. S3.

### Terpenoids

Phorbaketals A–C (**152**–**154**), three unprecedented sesterterpenoids with a spiroketal of hydrobenzopyran moiety, were isolated from *Phorbas* sp., which exhibited moderate to weak cytotoxicity against HT-29, HepG2, and A549 cell lines (Rho et al. 2009). Chemical investigation of *Hamigera* sp. led to the isolation of alotaketals A (**155**) and B (**156**), two unusual sesterterpenoids containing a spiroketal substructure, both of which activated the cAMP cell signaling pathway with EC_50_ values of 18 and 240 nmol/L, respectively (Forestieri et al. 2009). Nine triterpenoids were isolated from *Callyspongia* (= *Siphonochalina*) *siphonella*, of which compounds **157–162** reversed P-gp-mediated MDR to colchicine in resistant KB-C2 cells over-expressing P-gp (Jain et al. 2009). *Hippospongia lachne* was the source of eight acyclic manoalide-related sesterterpenes, hippolides A–H (**163**–**168**), of which hippolides A (**163**) and B (**164**) exhibited cytotoxic and moderate PTP1B inhibitory activities while hippolides A (**163**) and E (**167**) showed weak anti-inflammatory activity (Piao et al. 2011). Further examination of *H. lachne* gave additional five new hippolide derivatives, of which compounds **169** and **170** moderately inhibited PTP1B with IC_50_ values of 5.2 and 8.7 μmol/L, respectively (Piao et al. 2014). Phorbasones A (**171**) and B (**172**) were isolated from the marine sponge *Phorbas* sp., with phorbasone A displaying an induction of osteoblast differentiation (Rho et al. 2011). Examination of *Stylissa* cf.* massa* yielded two new amphilectane-type diterpenes, 8-isocyanato-15-formamidoamphilect-11(20)-ene (**173**) and 8-isothiocyanato-15-formamidoamphilect-11(20)-ene (**174**), both with antimalarial activity (Chanthathamrongsiri et al. 2012). *Rhabdastrella globostellata* afforded nine new isomalabaricane-type triterpenoids, globostelletins J–R, with globostelletins K (**175**) and L (**176**) moderately and selectively inhibiting ALK, FAK, Aurora-B, IGF-1R, SRC, and VEGF-R2 of 16 human tumor-related protein kinases (Li et al. 2012). Halichonadins K (**177**) and L (**178**) were sesquiterpene homodimers from *Halichondria* sp., with halichonadin K (**177**) displaying moderate cytotoxicity to the KB cell line (Tanaka et al. 2012). A *Phorbas* sp. marine sponge yielded ansellone B (**179**), phorbadione (**180**), secoepoxyansellone A (**181**), and alotaketal C (**182**), with alotaketal C (**182**) activating cAMP signaling in HEK cells. Ansellone B (**179**) possessed an unusual heterocyclic skeleton bearing an oxocane ring while secoepoxyansellone A (**181**) had the first degraded “secoansellane” carbon skeleton (Daoust et al. 2013). Homoscalarane sesterterpenes (**183**–**186**) showed different degrees of cytotoxicity with **183** and **184** being the most potent (IC_50_ = 0.26 and 0.28 μmol/L) (Harinantenaina et al. 2013). *Phorbas gukhulensis* were the source of diterpenoid pseudodimers, gukulenins C–F (**187**–**190**), all of which demonstrated significant cytotoxicity against K562 and A549 cell lines with IC_50_ values in the range of 0.04–0.55 μmol/L (Jeon et al. 2013). *Clathria gombawuiensis* produced three unprecedent tetracyclic sesterterpenes, gombaspiroketals A–C (**191**–**193**), all with moderate cytotoxic and antibacterial activities (Woo et al. 2014). Eleven new scalarane sesterterpenoids, carteriofenones A–K, were isolated from *Carteriospongia foliascens*, of which carteriofenone D (**194**) showed cytotoxicity against the P388 cell line (IC_50_ = 0.96 μmol/L) (Cao et al. 2015). Of eight new 4,9-friedodrimane-type sesquiterpenoids from a mixture of three sponges (*Smenospongia aurea*, *Smenospongia cerebriformis*, and *Verongula rigida*), compounds **195–198** suppressed β-catenin response transcription through degrading *β*-catenin and displayed cytotoxicity against colon cancer cells (Hwang et al. 2015). Niphateolide A (**199**) was isolated as an inseparable stereoisomeric mixture at C-17 from *Niphates olemda*, which was an inhibitor of p53-Hdm2 interaction (Kato et al. 2015). *Spongia ceylonensis* afforded seven new spongian diterpenes, ceylonamides A–F and 15α,16-dimethoxyspongi-13-en-19-oic acid, with ceylonamides A (**200**) and B (**201**) exhibiting RANKL-induced osteoclastogenesis with IC_50_ values of 13 and 18 μmol/L, respectively (El-Desoky et al. 2016). Darwinolide (**202**), an unprecedented rearranged spongian diterpene, was isolated as an inhibitor of MRSA biofilm from *Dendrilla membranosa* (von Salm et al. 2016). Three new furanosesterterpene tetronic acids, sulawesins A–C (**203**–**205**), were isolated from *Psammocinia* sp., all with inhibition of USP7 with IC_50_ values ranging from 2.7 to 4.6 μmol/L (Afifi et al. 2017). Hipposponlachnins A (**206**) and B (**207**), featuring an unusual tetracyclo [9.3.0.02,8.03,7] tetradecane carbon skeleton, were isolated from *H. lachne* and inhibited β-hexosaminidase release in anti-murine DNP-IgE-stimulated RBL-2H3 cells (Hong et al. 2017b). Further examination of *H. lachne* led to the isolation of a pair of unprecedented enantiomeric sesterterpenoids, ( ±)-hippolide J (**208**), both with potent antifungal activity with MIC_50_ values in the range of 0.125–0.25 μg/ml (Jiao et al. 2017). Dysiarenone (**209**), featuring an unusual carbon skeleton, was isolated as an inhibitor of COX-2 expression and prostaglandin E2 production from *Dysidea arenaria* (Jiao et al. 2018). The structures of compounds **152**–**209** are shown as supplementary Fig. S4.

### Polyketides

Franklinolides A–C (**210**–**212**) were unusual polyketide phosphodiesters featuring a rare 3-O-methylglyceric acid phosphodiester moiety from a sponge complex, of which franklinolides A (**210**) and B (**211**) displayed potent cytotoxic activity against five cancer cell lines with IC_50_ ranging from 1.1 to 2.5 μmol/L (Zhang et al. 2010). A two-sponge association of *Plakortis halichondroides* and *Xestospongia deweerdtae* produced two new ω-phenyl polyketide peroxides, plakinic acids K (**213**) and L (**214**), both with potent antifungal activity (MICs ≤ 0.5 μg/ml) (Dalisay et al. 2010). Bioassay (antitrypanosomal) guided fractionation of *Plakortis* sp. identified two new cyclic polyketide peroxides, 11,12-didehydro-13-oxo-plakortide Q (**215**) and 10-carboxy-11,12,13,14-tetranor-plakortide Q (**216**). Both compounds significantly inhibited growth of *Trypanosoma brucei brucei* with IC_50_ values of 49 and 940 nmol/L, respectively (Feng et al. 2010). Four additional polyketide endoperoxides, plakortides R–U (**217**–**220**), came from *Plakinastrella mamillaris*, of which plakortide U (**220**) was strongly active against the chloroquine-resistant FcM29 strain with an IC_50_ value of 0.8 μmol/L (Festa et al. 2013b). Examination of *Plakortis* cfr.* Lita* led to eight new endoperoxyketal polyketides, of which manadoperoxides F–I (**221**–**224**) and manadoperoxide K (**225**) displayed varying levels of antiprotozoal activity against *Trypanosoma brucei rhodesiense* and *Leishmania donovani* with IC_50_ values ranging from 0.062 to 5.73 μmol/L (Chianese et al. 2012). Chemical investigation of *Plakortis simplex* gave six new cyclic peroxides **226**–**231**, all with cytotoxic activity against RAW264.7 cells and antifungal activity against *Candida albicans* (Oh et al. 2013). Of the five new endoperoxide polyketides (**232**–**236**), obtained from *P. simplex*, all but **233** exhibited antimalarial activity against D10 and W2 *Plasmodium falciparum* strains (Chianese et al. 2014). *Plakortis bergquistae* yielded another five endoperoxide polyketides, manadodioxans A–E (**237**–**241**), with manadodioxan E (**241**) being active against *Escherichia coli* (Gushiken et al. 2015). *Plakortis angulospiculatus* was the source of **242**, which suppressed HCT-116 cells growth via inducing G_2_/M phase arrest and accumulating mitotic figures (Santos et al. 2015a). Bioassay-directed fractionation of sponges *Xestospongia testudinaria* and *Xestospongia* sp. led to the isolation of xestosaprol C methylacetal (**243**) and orhalquinone (**244**), both with potent inhibition of yeast farnesyltransferase (IC_50_ = 0.40 and 6.71 μmol/L) (Longeon et al. 2010). Simplextones A (**245**) and B (**246**), identified from *P. simplex*, featured an unprecedented carbon skeleton with the connection of two cyclopentanes through a single carbon–carbon bond, both of which showed weak cytotoxic activity (Liu et al. 2011). *P. mamillaris* gave seven new oxygenated polyketides with plakilactone C (**247**) able to selectively activate PPARγ with an EC_50_ value of 2 μmol/L (Festa et al. 2012b). Further examination of *P. mamillaris* led to the discovery of one additional oxygenated polyketide, gracilioether K (**248**), with potent pregnane-X-receptor (PXR) agonistic activity (Festa et al. 2013a). *P. simplex* yielded a new plakorsin D methyl ester (**249**), plakilactone I (**250**), plakortone Q (**251**), and plakdiepoxide (**252**), of which plakdiepoxide (**252**) was a selective ligand of PPAR-γ (Chianese et al. 2016). Six butyrate-derived polyketides, simplexolides A–E (**253**–**257**) and plakorfuran A (**258**), were identified from *P. simplex*. Simplexolides B (**254**) and E (**257**) showed weak to moderate antifungal activity while simplexolide B (**254**) also displayed moderate cytotoxic and weak antileismanial activities (Liu et al. 2012). Another investigation of *P. simplex* gave further five polyketides, plakortoxides A (**259**) and B (**260**), simplextones C (**261**) and D (**262**), and plakorsin D (**263**), of which compound **3** was significantly active against c-Met kinase (Zhang et al. 2013). Woodylides A–C (**264**–**266**) were sourced from *P. simplex*, of which woodylides A (**264**) and C (**266**) showed moderate cytotoxic and antifungal activity and woodylide C (**266**) displayed moderate PTP1B inhibitory activity (Yu et al. 2012). *Plakortis* cfr.* Lita* yielded two new endoperoxyketal polyketides, 12-isomanadoperoxide B (**267**) and manadoperoxidic acid B (**268**), both with strong antitrypanosomal (IC_50_ = 11 ng/ml and 1.87 μg/ml) and moderate cytotoxic (IC_50_ = 3.80 and 7.12 μg/ml) activities (Chianese et al. 2013). PM050489 (**269**) and PM060184 (**270**) were unusual polyketides from *Lithoplocamia lithistoides* with potent cytotoxic activity (IC_50_ ≤ 0.61 μmol/L), excellent antimitotic properties, and distinct inhibition mechanisms on microtubules (Martín et al. 2013). Examination of *H. lachne* yielded hippolachnin A (**271**), possessing an unprecedented four-membered ring moiety, which showed potent antifungal activity with an MIC value of 0.41 μmol/L (Piao et al. 2013). Plakortinic acids A (**272**) and B (**273**) were inseparable endoperoxide polyketides with a bicyclo[4.2.0]octene unit from a symbiotic association *Plakortis halichondrioides*–*X. deweerdtae*, which was strongly active against DU-145 prostate and versus A2058 melanoma cancer cells with IC_50_ values of 0.5 and 0.3 μmol/L, respectively (Jimenez-Romero et al. 2017). *Petrosaspongia* sp. was the source of biakamides A–D (**274**–**277**). All compounds showed selective cytotoxic activities against PANC-1 cells cultured under glucose-deficient conditions (IC_50_ = 0.5–4.0 μmol/L) via inhibiting complex I in the mitochondrial electron transport chain (Kotoku et al. 2017). The structures of compounds **210**–**277** are shown as supplementary Fig. S5.

### Hydroxybenzenes/Quinones

Examination of *Dysidea* sp. gave a new sesquiterpene aminoquinone, dysideamine (**278**), having neuroprotective effects and inhibiting production of ROS in the IAA-treated HT22 cells (Suna et al. 2009). Nakijiquinones J–R (**279**–**287**) were sesquiterpenoid quinones from an unidentified sponge, some of which exhibited inhibitory activities against EGFR and HER2 tyrosine kinases (Takahashi et al. 2010). Chemical investigation of *Dactylospongia elegans* yielded three new sesquiterpene benzoxazoles/quinones, nakijinol B (**288**) and smenospongines B–C (**289**–**290**), which showed weak to moderate cytotoxicity against a panel of human tumor cell lines (Ovenden et al. 2011). Diplopuupehenone (**291**) was a new unsymmetrical puupehenone-related dimer from *Dysidea* sp. with moderate DPPH radical scavenging activity (Utkina et al. 2011). *Tedania ignis* was the source of two new strained cyclic diarylheptanoids, tedarenes A (**292**) and B (**293**), with tedarene A (**292**) inhibiting LPS-induced NO_2_^−^ production (Costantino et al. 2012). Bioassay-guided fractionation of *Petrosia alfiani* yielded three new xewstoquinones, 14-hydroxymethylxestoquinone (**294**), 15-hydroxymethylxestoquinone (**295**), and 14,15-dihydroxestoquinone (**296**). All compounds showed different degrees of cytotoxicity, of which 14-hydroxymethylxestoquinone (**294**) may act as to uncouple mitochondrial respiration and oxidative phosphorylation (Du et al. 2012). Examination of *Dysidea avara* afforded four new sesquiterpene quinones, dysidavarones A–D (**297**–**300**), with dysidavarones A (**297**) and D (**300**) showing cytotoxicity and inhibitory activity on PTP1B (Jiao et al. 2012). Of five new sesquiterpene quinone/phenols (**301**–**305**) from *D. elegans*, 5,8-diepi-ilimaquinone (**301**) and 4,5-diepi-dactylospongiaquinone (**302**) featuring a 2-hydroxy-5-methoxy-1,4-benzoquinone moiety activated HIF-1 and increased the expression of HIF-1 target gene VEGF in T47D cells (Du et al. 2013). NMR-directed fractionation of *Hamigera tarangaensis* led to the isolation of ten new hamigerans (**306**–**405**), all of which were active against HL-60 cells (Singh et al. 2013). Two merotriterpenoid hydroquinone sulfates, adociasulfates-13 (**406**) and -14 (**407**) were isolated as inhibitor of microtubule-stimulated kinesin ATPase from *Cladocroce aculeata* (Smith et al. 2013). *Sarcotragus spinosulus* yielded one polyprenyl-1′,4′-hydroquinone derivative, hydroxyoctaprenyl-1′,4′-hydroquinone (**408**), which significantly modulated the release of acetylcholine and glutamate in the rat cortex and hippocampus (Bisio et al. 2014). Of unprecedented dysideanones A–C (**409**–**411**) from *D. avara*, dysideanone B (**410**) showed cytotoxicity against HeLa and HepG2 cells (IC_50_ = 7.1 and 9.4 μmol/L) (Jiao et al. 2014a). Of 13 new sesquiterpene aminoquinones from *Dysidea fragilis*, dysidaminones C (**412**), E (**413**), H (**414**), and J (**415**), 18-aminosubstituted sesquiterpene quinones with exocyclic double bond (Δ^4,11^), showed cytotoxicity against several cancer cell lines and exhibited NF-κB inhibitory activity (IC_50_ = 0.11–9.65 μmol/L) (Jiao et al. 2014b). Eight new sesquiterpene quinol/quinones, dysiquinols A–D (**416**–**419**), (5S,8S,9R,10S)-18-ethoxyneoavarone (**420**), (5S,8S,9R,10S)-19-ethoxyneoavarone (**421**), (5R,8R,9S,10R)-18-ethoxyavarone (**422**), and (5R,8R,9S,10R)-19-ethoxyavarone (**423**), were sourced from *D. avara*. All of them were active against NCI-H929 cells, but only dysiquinol D (**419**) displayed NF-κB inhibitory activity (IC_50_ = 0.81 μmol/L) (Jiao et al. 2015a). Three sesquiterpene aminoquinones with an unusual rearranged avarone skeleton, dysifragilones A–C (**424**–**426**), were isolated as inhibitors of NO production from *D. fragilis* (Jiao et al. 2015b). *Spongia* sp. afforded two additional sesquiterpene aminoquinones, langcoquinones A (**427**) and B (**428**), both with antibacterial activities (Nguyen et al. 2016). Three new sesquiterpene hydroquinones, avapyran (**429**), 17-O-acetylavarol (**430**), and 17-O-acetylneoavarol (**431**), were obtained as inhibitors of PTP1B from a *Dysidea* sp. marine sponge (Abdjul et al. 2016b). *S. cerebriformis* afforded one new sesquiterpene quinone, smenohaimien F (**432**), with moderate cytotoxic activities (Huyen et al. 2017). *Spongia pertusa* Esper produce nine new sesquiterpene quinone/hydroquinones (**433**–**441**), of which compound **438** demonstrated CDK-2 affinity (K_d_ = 4.8 μmol/L) in a surface plasmon resonance assay (Li et al. 2017). Of three new sesquiterpene aminoquinones, coquinones D–F (**442**–**444**), from *Spongia* sp., only langcoquinone D (**442**) exhibited cytotoxic and antibacterial activities (Ito et al. 2018). The structures of compounds **278**–**444** are shown as supplementary Fig. S6.

### Lipids

Chemical investigation of *Siliquariaspongia* sp. yielded motualevic acids A–F (**445**–**450**) and (4E)-(R)-antazirine (**451**), of which motualevic acids A–D (**445**–**448**) were unprecedently glycyl conjugates of the ω-brominated lipid (E)-14,14-dibromotetradeca-2,13-dienoic acid, and motualevic acid F (**450**) was a rare long-chain 2H-azirine 2-carboxylic acid. Compounds **445** and **450** showed antibacterial activity against *Staphylococcus aureus* and MRSA (MIC_50_ = 1.2–10.9 μg/ml) (Keffer et al. 2009). *Reniochalina* sp. produced two new acetylenic alcohols (**452**–**453**) and a new dihydrothiopyranone (**454**), with compound **452** displaying significantly cytotoxicity against several human tumor cell lines (Lee et al. 2009). Carteriosulfonic acids A–C (**455**–**457**) were isolated as GSK-3β inhibitors from a *Carteriospongia* sp. marine sponge (Mcculloch et al. 2009). *Penares* sp. were the source of penasins A–E (**458**–**462**), with penasins C–E (**460**–**462**) isolated as an inseparable mixture. All of them displayed moderate cytotoxic activity against HeLa cells (Ando et al. 2010). Bioassay-guided fractionation of *Spongia* (*Heterofibria*) sp. gave fatty acids heterofibrins A1 (**463**) and B1 (**466**), as well as related actyl esters, heterofibrins A2 (**464**), B2 (**467**), A3 (**465**), and B3(**468**), with heterofibrins A1 (**463**) and B1 (**466**) inhibiting lipid droplet formation in A431 fibroblast cells (Salim et al. 2010). Examination of *Petrosia* sp. led to isolation of six linear acetylenes, ( −)-duryne (**469**) and ( −)-durynes B–F (**470**–**474**), all of which showed cytotoxicity against HeLa cells with IC_50_ values ranging from 0.08 to 0.50 μmol/L (Hitora et al. 2011). *Spirastrella mollis* yielded an unprecedent long-chain chlorodibromohydrin amide, mollenyne A (**475**), with significant cytotoxicity against HCT-116 cells (IC_50_ = 1.3 μg/ml) (Morinaka and Molinski 2011). *Placospongia* sp. afforded two unprecedented phosphorus-containing iodinated polyacetylenes, phosphoiodyns A (**476**) and B (**477**), with phosphoiodyn A (**476**) being an inhibitor of hPPARδ (EC_50_ = 23.7 nmol/L) (Kim et al. 2013). ( −)-Petrosynoic acids A–D (**478**–**481**) from *Petrosia* sp. displayed cytotoxicity to three human cancer cell lines and IMR-90 quiescent human fibroblast cells (Mejia et al. 2013). Manzamenone O (**482**) was an antimicrobial trimeric fatty acid derivative from *Plakortis* sp., which possessed a novel skeleton containing C–C bonded octahydroindenone, dioxabicyclo[3.3.0]octane moieties, and three long aliphatic chains (Tanaka et al. 2013a). Placotylenes A (**483**) and B (**484**) were isolated from *Placospongia* sp., with placotylene A (**483**) inhibiting RANKL-induced osteoclast differentiation (Kim et al. 2014). An unidentified sponge led to the isolation of taurospongins B (**1**) and C (**2**), of which taurospongin C (**2**) showed weak activity against *Cryptococcus neoformans* (Kubota et al. 2014). Six new polyacetylenic alcohols, strongylotriols A–B (**485**–**486**), isopellynol A (**487**), and pellynols J–L (**488**–**490**), were sourced from *Petrosia* sp. and *Halichondria* sp., with all but **489** showing cytotoxicity against HeLa and K562 cell lines with IC_50_ values ranging from 0.55 to 18.1 μmol/L (Gabriel et al. 2015). Isopetrosynol (**491**) was isolated as an inhibitor of PTP1B (IC_50_ = 8.2 ± 0.3 μmol/L) from *Halichondria* cf.* panicea* (Abdjul et al. 2016a). Two new prolyl amides of polyoxygenated fatty acids, yakushinamides A (**492**) and B (**493**), were isolated as inhibitors of HDACs and SIRTs from *T. swinhoei* (Takada et al. 2016). Monanchoramides A–D (**494**–**497**) were isolated from the sponge *Monanchora clathrata*, of which monanchoramide A (**494**) showed weak to moderate cytotoxicity against MES-SA, MCF-7, and HK-2 cell lines (Raslan et al. 2018). Bioassay-guided fractionation of *Niphates* sp. led to the isolation of pellynols M–O (**498**–**500**), each of which inhibited PC9 and HepG2 human cancer cell lines growth with IC_50_ values of 2.9–7.6 μmol/L (Wang et al. 2018). The structures of compounds **445**–**500** are shown as supplementary Fig. S7.

### Steroids

Bioassay-guided fractionation of an antifungal extract from *Topsentia* sp. yielded two new sulfated sterols, geodisterol-3-O-sulfite (**501**) and 29-demethylgeodisterol-3-O-sulfite (**502**), both of which reversed efflux pump-mediated fluconazole resistance in an *S. cerevisiae* strain overexpressing the *C. albicans* efflux pump *MDR1* and in a fluconazole-resistant *C. albicans* clinical isolate (Di Girolamo et al. 2009). *Phorbas amaranthus* produced two new sulfated dimeric sterols, amaroxocanes A (**503**) and B (**504**), with amaroxocane B (**504**) being an effective antifeedant (Morinaka et al. 2009). Three additional sterol dimers, fibrosterol sulfates A–C (**505**–**507**), were identified from *Lissodendoryx* (*Acanthodoryx*) *fibrosa*. Fibrosterol sulfates A (**505**) and B (**506**) were inhibitors of PKCζ (IC_50_ = 16.4 and 5.6 μmol/L) (Whitson et al. 2009). *Topsentia* sp. yielded 24-isopropyl steroids, topsentinols K (**508**), L (**509**), and K trisulfate (**510**), of which topsentinol K trisulfate (**510**) was a potent inhibitor of the aspartic protease BACE1 with an IC_50_ value of 1.2 μmol/L (Dai et al. 2010). Four new polyhydroxy sterols **511**–**514** came from *Callyspongia fibrosa* with all but **512** displaying moderate antimalarial activity against *Plasmodium falciparum* (Rao et al. 2010). Solomonsterols A (**515**) and B (**516**), unprecedented C-24 and C-23 sulfated sterols, were the first marine natural PXR agonists isolated from *T. swinhoei* (Festa et al. 2011a). Further examination of *T. swinhoei* gave one additional potent agonist of PXR, malaitasterol A (**517**), and one dual FXR and PXR agonist, conicasterol E (**518**) (De Marino et al. 2011b; Sepe et al. 2012). *Crella* (*Yvesia*) *spinulata* was the source of two dimeric steroid derivatives, shishicrellastatins A (**519**) and B (**520**), both with moderate inhibition of cathepsin B (IC_50_ = 8 μg/ml) (Murayama et al. 2011). Two new sulfonated sterol dimers, manadosterols A (**521**) and B (**522**), were isolated as inhibitors of Ubc13-Uev1A interaction (IC_50_ = 0.09 and 0.13 μmol/L) from *Lissodendryx fibrosa* (Ushiyama et al. 2012). *Haliclona crassiloba* produced halicrasterols A–D (**523**–**526**) with halicrasterol D (**526**) displaying antibacterial activity against *Escherichia faecalis* ATCC 29212 with an MIC value of 4 μg/ml (Cheng et al. 2013). Chemical investigation of *X. testudinaria* led to the isolation of three new 26,27-cyclosterols, aragusterol I (**527**), 21-O-octadecanoyl-xestokerol A (**528**), and 7β-hydroxype-trosterol (**529**), of which 21-O-octadecanoyl-xestokerol A (**528**) was a potent antifouling substance (Nguyen et al. 2013). Swinhoeisterols A (**530**) and B (**531**), new steroids with a rearranged skeleton featuring an unusual 6/6/5/7-tetracyclic ring system, were isolated as (h)P300 inhibitors from *T. swinhoei* (Gong et al. 2014). Further examination of *T. swinhoei* led to additional swinhoeisterols C–F (**532**–**535**) with swinhoeisterol C (**532**) inhibiting (h)p300 with an IC_50_ value of 8.8 μmol/L (Li et al. 2018). *Cinachyrella* sp. yielded cinanthrenol A (**536**), a new steroid containing a phenanthrene and a spiro[2,4]heptane system, which demonstrated cytotoxicity against P-388 (IC_50_ = 4.5 μg/ml) and HeLa cells (IC_50_ = 0.4 μg/ml) and estrogen activity (IC_50_ = 10 nmol/L) (Machida et al. 2014). 24-vinyl-cholest-9-ene-3β,24-diol (**537**) and 20-methyl-pregn-6-en-3β-ol,5α,8α-epidioxy (**538**) were isolated from *Haliclona simulans*, both with antitrypanosomal and anti-mycobacterial activities (Viegelmann et al. 2014). Bioassay-guided fractionation of the extract of *Polymastia boletiformis* gave two new sulfated steroid-amino acid conjugates (**539** and **540**), both with moderate antifungal activity (Smyrniotopoulos et al. 2015). Examination of *Monanchora* sp. led to identification of monanchosterols A (**541**) and B (**542**), representing the first examples of steroids featuring the bicyclo[4.3.1] A/B ring system, as well as compound **543**. Compounds **542** and **543** significantly inhibited mRNA expression of IL-6 with IC_50_ values of 5.0 ± 0.17 and 5.2 ± 0.30 μmol/L, respectively (Wang et al. 2015b). Six new polyoxygenated steroids, gombasterols A–F (**544**–**549**), were sourced from *C. gombawuiensis*, of which **544**–**545** and **548**–**549** moderately enhanced 2-NBDG uptake in differentiated 3T3-L1 adipocytes and phosphorylation of AMPK and ACC in differentiated mouse C2C12 skeletal myoblasts (Woo et al. 2017). *Inflatella* sp. yielded four new oxysterols **550**–**553** with compound **553** displaying essential neuroprotective activity in a 6-OHDA-induced model of Parkinson’s disease, probably via a ROS scavenging effect (Kolesnikova et al. 2018). The structures of compounds **501**–**553** are shown as supplementary Fig. S8.

## Conclusions and outlooks

Marine sponges continue to be prolific producers of structurally diverse compounds with valuable therapeutic potential. In this review, we summarize sponge-derived new compounds over the years 2009–2018 in terms of published year, chemical class, sponge taxonomy, and biological activity. The number of new compounds gradually decreased probably because natural product chemists turned their research focus to sponge symbiotic microorganisms which may be the real producers of bioactive compounds. More than half of new metabolites reported during this period showed biological activity. The major reported bioactivities were anticancer/cytotoxic activity (49.1%), antibacterial activity (13.1%), enzyme inhibition activity (8.2%), antifungal activity (6.3%), and antimalarial activity (4.1%). All chemical groups displayed cytotoxicity as a dominant activity. Alkaloids (823) and terpenoids (693) represented two main structural types of new compounds, adding up to more than half of the total. Within the most prolific class Demospongiae, Orders Dictyoceratida, Haplosclerida, Poecilosclerida, and Tetractinellida contributed the largest quantities, producing 595, 455, 406, and 327 new compounds, respectively. Structural novelty and excellent pharmacological activities of some representative compounds are highlighted.

It should be noted that the statistical results of new bioactive compounds are not comprehensive and influenced by many factors. First, not all new metabolites isolated from sponges were tested for biological activity because of scarcity of quantity. Second, many bioactive compounds were only studied for one or two types of bioassays due to lack of effective biological activity screening models. Third, bioactivity screening of new compounds from marine sponges probably depends on research funding, government policy, research facilities, industrial investment, the professional knowledge of scientists, and so on. On the basis of the foregoing, more sponge-derived new natural products should be screened on a wider variety of bioassays, suggesting that effective enrichment of trace compounds and enhanced methods in bioactivity screening technologies are important.

Based on the summary above, the potential of marine sponges as prolific sources of novel bioactive compounds in marine drugs research and development is undisputed. There are still plenty of molecules with therapeutic potential to be discovered from sponges. It is worth mentioning that sponges as animal hosts are important microbial fermenters. The discovery of huge microbial diversity in sponges, the true producers of secondary metabolites, the mass production of trace amounts of compounds by symbiotic microorganisms, and the symbiotic relationship between sponge host and microorganisms make marine sponges very important and provide many interesting research opportunities.

## Supplementary Information

Below is the link to the electronic supplementary material.

Supplementary file1 (DOCX 4207 KB)

Supplementary file2 (DOCX 139 KB)

## Data Availability

All data generated or analyzed during this study are included in the manuscript and supporting files.
