# Supplementary material for: Chemical and biological diversity of new natural products from marine sponges: a review (2009–2018)
Source: Mar Life Sci Technol. 2022 Aug 1;4(3):356–72. doi: 10.1007/s42995-022-00132-3 (PMC10077299; doi:10.1007/s42995-022-00132-3)
Supplement: Supplementary file 1 — Supplementary file1 (DOCX 4207 KB) [file 42995_2022_132_MOESM1_ESM.docx]

Supporting Information for

Chemical and biological diversity of new natural products from marine sponges: A review (2009−2018)

Li-Li Hong^1^, Ya-Fang Ding^1,2^, Wei Zhang^3, *^, Hou-Wen Lin^1, *^

^1^Research Center for Marine Drugs, State Key Laboratory of Oncogenes and Related Genes, Department of Pharmacy, Ren Ji Hospital, School of Medicine, Shanghai Jiao Tong University, Shanghai 200127, China

^2^School of Food and Pharmacy, Zhejiang Ocean University, Zhoushan 316000, China

^3^Centre for Marine Bioproducts Development, Flinders University, Adelaide SA 5042, Australia

*Corresponding authors

*Email address*: wei.zhang@flinders.edu.au (Wei Zhang), franklin67@126.com (Hou-Wen Lin).

Contents

[Fig. S1. Structures of representative macrolides isolated from marine sponges for 2009−2018 2](#_Toc87626726)

[Fig. S2. Structures of representative peptides isolated from marine sponges for 2009−2018 3](#_Toc87626727)

[Fig. S3. Structures of representative alkaloids isolated from marine sponges for 2009−2018 4](#_Toc87626728)

[Fig. S4. Structures of representative terpenoids isolated from marine sponges for 2009−2018 5](#_Toc87626729)

[Fig. S5. Structures of representative polyketides isolated from marine sponges for 2009−2018 6](#_Toc87626730)

[Fig. S6. Structures of representative hydroxybenzene/quinone isolated from marine sponges for 2009−2018 7](#_Toc87626731)

[Fig. S7. Structures of representative lipids isolated from marine sponges for 2009−2018 8](#_Toc87626732)

[Fig. S8. Structures of representative steroids isolated from marine sponges for 2009−2018 9](#_Toc87626733)

Fig. S1. Structures of representative macrolides isolated from marine sponges for 2009−2018

Fig. S2. Structures of representative peptides isolated from marine sponges for 2009−2018

Fig. S3. Structures of representative alkaloids isolated from marine sponges for 2009−2018

Fig. S4. Structures of representative terpenoids isolated from marine sponges for 2009−2018

Fig. S5. Structures of representative polyketides isolated from marine sponges for 2009−2018

Fig. S6. Structures of representative hydroxybenzene/quinone isolated from marine sponges for 2009−2018

Fig. S7. Structures of representative lipids isolated from marine sponges for 2009−2018

Fig. S8. Structures of representative steroids isolated from marine sponges for 2009−2018
