# Supplementary material for: Chemical and biological diversity of new natural products from marine sponges: a review (2009–2018)
Source: Mar Life Sci Technol. 2022 Aug 1;4(3):356–72. doi: 10.1007/s42995-022-00132-3 (PMC10077299; doi:10.1007/s42995-022-00132-3)
Supplement: Supplementary file 2 — Supplementary file2 (DOCX 139 KB) [file 42995_2022_132_MOESM2_ESM.docx]

Supporting Information for

Chemical and biological diversity of new natural products from marine sponges: A review (2009−2018)

Li-Li Hong^1^, Ya-Fang Ding^1,2^, Wei Zhang^3, *^, Hou-Wen Lin^1, *^

^1^Research Center for Marine Drugs, State Key Laboratory of Oncogenes and Related Genes, Department of Pharmacy, Ren Ji Hospital, School of Medicine, Shanghai Jiao Tong University, Shanghai 200127, China

^2^School of Food and Pharmacy, Zhejiang Ocean University, Zhoushan 316000, China

^3^Centre for Marine Bioproducts Development, Flinders University, Adelaide SA 5042, Australia

*Corresponding authors

*Email address*: wei.zhang@flinders.edu.au (Wei Zhang), franklin67@126.com (Hou-Wen Lin).

Table S1. The list of articles about new compounds isolated from marine sponges published during 2009−2018.

| Article | Published Year | Journal |
| --- | --- | --- |
| Amphiceramide A and B, novel glycosphingolipids from the marine sponge *Amphimedon compressa* | 2009 | European Journal of Organic Chemistry |
| (-)-Calcaridine B, a new chiral aminoimidazole-containing alkaloid from the marine sponge *Leucetta chagosensis* | 2018 | Journal of Asian Natural Products Research |
| (-)-Duryne and its homologues, cytotoxic acetylenes from a marine sponge *Petrosia* sp | 2011 | Journal of Natural Products |
| (+)- And (-)-spiroreticulatine, a pair of unusual spiro bisheterocyclic quinoline-imidazole alkaloids from the South China Sea sponge *Fascaplysinopsis reticulata* | 2015 | Organic Letters |
| (+)-7-Bromotrypargine: an antimalarial β-carboline from the Australian marine sponge *Ancorina* sp | 2010 | Tetrahedron Letters |
| (±)-Hippolide J - a pair of unusual antifungal enantiomeric sesterterpenoids from the marine sponge *Hippospongia lachne* | 2017 | European Journal of Organic Chemistry |
| 1,5-Diazacyclohenicosane, a new cytotoxic metabolite from the marine sponge *Mycale* sp | 2009 | Marine Drugs |
| ^1^Hand ^13^C NMR assignments of sesquiterpenes from *Dysidea fragilis* | 2015 | Magnetic Resonance in Chemistry |
| 1-Hydroxyethylhalenaquinone: a new proteasome inhibitor from the marine sponge *Xestospongia* sp | 2014 | Heterocycles |
| 2-Debromonagelamide U, 2-debromomukanadin G, and 2-debromonagelamide P from marine sponge *Agelas* sp | 2015 | Heterocycles |
| 2-Palmitamidoethanesulfonic acid, a taurine derivative from the marine sponge *Haliclona* sp | 2009 | Chemistry of Natural Compounds |
| 3-Alkylpyridinium alkaloids from the Pacific sponge *Haliclona* sp | 2009 | Journal of Natural Products |
| 3-Alkylpyridinium salts from *Haplosclerida* marine sponges: isolation, structure elucidations, and biosynthetic considerations | 2009 | Pure and Applied Chemistry |
| 3-Oxoabolene and 1-oxocurcuphenol, aromatic bisabolanes from the sponge *Myrmekioderma* sp | 2013 | Natural Product Communications |
| 4-Methylenesterols from a sponge *Theonella swinhoei* | 2012 | Marine Drugs |
| 4-Methylenesterols from *Theonella swinhoei* sponge are natural pregnane-X-receptor agonists and farnesoid-X-receptor antagonists that modulate innate immunity | 2012 | Steroids |
| 5-Alkylpyrrole-2-carboxaldehyde derivatives from the Chinese sponge *Mycale lissochela* and their PTP1B inhibitory activities | 2017 | Chinese Chemical Letters |
| 5-Hydroxyindole-type alkaloids, as Candida albicans isocitrate lyase inhibitors, from the tropical sponge *Hyrtios* sp | 2009 | Bioorganic & Medicinal Chemistry Letters |
| 5α,8α-epidioxysterols from a formosan sponge, *Axinyssa* sp | 2013 | Natural Product Communications |
| 6-Bromoindole derivatives from the Icelandic marine sponge *Geodia barretti*: isolation and anti- inflammatory activity | 2018 | Marine Drugs |
| 8-Isocyanoamphilecta-11(20),15-diene, a new antimalarial isonitrile diterpene from the sponge *Ciocalapata* sp | 2009 | Canadian Journal of Chemistry |
| 9'-Epi-3β,3'β-dimethylxestospongin C, a new macrocyclic diamine alkaloid from the Hainan sponge *Neopetrosia exigua* | 2011 | Planta Medica |
| A bastadin with potent and selective δ-opioid receptor binding affinity from the Australian sponge *Ianthella flabelliformis* | 2010 | Journal of Natural Products |
| A chemical investigation of the Antarctic sponge *Lyssodendoryx flabellata* | 2012 | Natural Product Research |
| A grand challenge: unbiased phenotypic function of metabolites from *Jaspis splendens* against Parkinson’s disease | 2016 | Journal of Natural Products |
| A new 1,4-diazepine from South China Sea marine sponge *Callyspongia* species | 2010 | Molecules |
| A new 3-alkylpyridine alkaloid from the marine sponge *Haliclona* sp. and its cytotoxic activity | 2016 | Natural Product Research |
| A new 9,11-secosterol with a 1,4-quinone from a Korean marine sponge *Ircinia* sp | 2015 | Archives of Pharmacal Research |
| A new acyclic thiophene sesterterpene from the Sikao Bay sponge, *Xestospongia* sp | 2011 | Journal of Asian Natural Products Research |
| A new antimicrobial fatty acid from the Calcareous sponge *Paragrantia cf. waguensis* | 2009 | Chemistry & Biodiversity |
| A new antitrypanosomal alkaloid from the Red Sea marine sponge *Hyrtios* sp | 2018 | The Journal of Antibiotics |
| A new bioactive metabolite isolated from the Red Sea marine sponge *Hyrtios erectus* | 2016 | Molecules |
| A new bioactive sesquiterpenoid quinone from the Mediterranean sea marine sponge *Dysidea avara* | 2013 | Natural Product Communications |
| A new bioactive steroidal ketone from the South China Sea sponge *Xestospongiatestudinaria* | 2016 | Journal of Asian Natural Products Research |
| A new C29-sterol with a cyclopropane ring at C-25 and 26 from the Vietnamese marine sponge *Ianthella* sp | 2009 | Archives of Pharmacal Research |
| A new ceramide from a new species of *Spongia* sponge | 2010 | Chemistry of Natural Compounds |
| A new cytotoxic brominated acetylenic hydrocarbon from the marine sponge *Haliclona* sp. with a selective effect against human breast cancer | 2013 | Zeitschrift fur Naturforschung Section C-A Journal of Biosciences |
| A new cytotoxic polyacetylenic alcohol from a sponge *Callyspongia* sp. | 2017 | Natural Product Communications |
| A new diketopiperazine from South China Sea marine sponge *Callyspongia* sp | 2014 | Natural Product Research |
| A new diketopiperazine from the marine sponge *Callyspongia* species | 2016 | Records of Natural Products |
| A new diketopiperazine, cyclo-(4-S-hydroxy-R-proline-R-isoleucine), from an Australian specimen of the sponge *Stelletta* sp | 2011 | Marine Drugs |
| A new hydroxylated nonaprenylhydroquinone from the Mediterranean marine sponge *Sarcotragus spinosulus* | 2011 | Marine Drugs |
| A new imidazole from the sponge *Dercitus (Halinastra) japonensis* | 2017 | Natural Product Communications |
| A new kalihinol diterpene from the Hainan sponge *Acanthella* sp | 2009 | Archives of Pharmacal Research |
| A new N,N-dimethyl purine from an Australian *dictyoceratid* sponge | 2011 | Journal of Chemical Crystallography |
| A new N-acyl taurine from the South China Sea marine sponge *Callyspongia* sp | 2015 | Chemistry of Natural Compounds |
| A new norsesterterpenoid from the sponge species *Sarcotragus* | 2012 | Chemistry of Natural Compounds |
| A new polyunsaturated brominated fatty acid from a *Haliclona* sponge | 2009 | Marine Drugs |
| A new sarasinoside congener, sarasinoside M2, from a marine sponge collected in the Solomon Islands | 2017 | Bioscience, Biotechnology, and Biochemistry |
| A new sesterterpene from the Korean *Sarcotragus* sp. sponge | 2015 | Natural Product Sciences |
| A new sesterterpenoid showing anti-inflammatory effect from the Marine sponge *Haliclona* species | 2015 | Journal of the Korean Magnetic Resonance Society |
| A new spongilipid from the freshwater sponge *Spongilla lacustris* | 2009 | Bulletin of the Korean Chemical Society |
| A new sterol from the Vietnamese marine sponge *Xestospongia testudinaria* and its biological activities | 2018 | Natural Product Research |
| A new taurine derivative from South China Sea marine sponge *Axinella* sp | 2013 | Natural Product Research |
| A norsesterterpene peroxide from a marine sponge *Hippospongia* sp | 2016 | Natural Product Communications |
| A novel natural phenyl alkene with cytotoxic activity | 2013 | Tetrahedron Letters |
| A novel sesquiterpene quinone from Hainan sponge *Dysidea villosa* | 2009 | Bioorganic & Medicinal Chemistry Letters |
| A search for BACE inhibitors reveals new biosynthetically related pyrrolidones, furanones and pyrroles from a southern Australian marine sponge, *Ianthella* sp | 2012 | Organic & Biomolecular Chemistry |
| A search for kinase inhibitors and antibacterial agents: bromopyrrolo-2-aminoimidazoles from a deep-water Great Australian Bight sponge, *Axinella* sp | 2012 | Tetrahedron Letters |
| A tetrachloro polyketide hexahydro-1h-isoindolone, muironolide a, from the marine sponge *Phorbas* sp. natural products at the nanomole scale | 2009 | Journal of the American Chemical Society |
| A tetramic acid derivative with protein tyrosine phosphatase 1B inhibitory activity and a new nortriterpene glycoside from the Indonesian marine sponge *Petrosia* sp | 2017 | Bioorganic & Medicinal Chemistry Letters |
| A β-carboline alkaloid from the Papua New Guinea marine sponge *Hyrtios reticulatus* | 2010 | Journal of Natural Products |
| Aaptamine alkaloids from the Vietnamese sponge *Aaptos* sp | 2009 | Natural Product Communications |
| Aaptamine derivatives from the Indonesian sponge *Aaptos suberitoides* | 2013 | Journal of Natural Products |
| Aaptamine derivatives with antifungal and anti-HIV-1 activities from the South China Sea sponge *Aaptos* *aaptos* | 2014 | Marine Drugs |
| Aaptamines, marine spongean alkaloids, as anti-dormant mycobacterial substances | 2014 | Journal of Natural Medicines |
| Aaptanone, a novel zwitterionic metabolite of the aaptamine class with an oxygenated 1,6-naphthyridine core from the Vietnamese marine sponge *Aaptos aaptos* | 2009 | Tetrahedron Letters |
| Aaptoline A, a new quinoline alkaloid from the marine sponge *Aaptos suberitoides* | 2014 | Heterocycles |
| Absolute configuration of the new 3-epi-cladocroic acid from the Mediterranean sponge *Haliclona fulva* | 2013 | Metabolites |
| Absolute structures and bioactivities of euryspongins and eurydiene obtained from the marine sponge *Euryspongia* sp. collected at Iriomote Island | 2015 | Bioorganic & Medicinal Chemistry |
| Acanthifoliosides, minor steroidal saponins from the Caribbean sponge *Pandaros acanthifolium* | 2011 | Tetrahedron |
| Acanthocyclamine A from the Indonesian marine sponge *Acanthostrongylophora ingens* | 2014 | Australian Journal of Chemistry |
| Acantholactam and pre-neo-kauluamine, manzamine-related alkaloids from the Indonesian marine sponge *Acanthostrongylophora ingens* | 2014 | Journal of Natural Products |
| Acantholactone, a new manzamine related alkaloid with an unprecedented δ-lactone and ε-lactam ring system | 2012 | Tetrahedron Letters |
| Acanthomanzamines A-E with new manzamine frameworks from the marine sponge *Acanthostrongylophora ingens* | 2014 | Organic Letters |
| Additional bioactive guanidine alkaloids from the Mediterranean sponge *Crambe crambe* | 2012 | RSC Advances |
| Additional insights on the bastadins: isolation of analogues from the sponge *Ianthella cf. reticulata* and exploration of the oxime configurations | 2010 | Journal of Natural Products |
| Additional sesterterpenes and a nortriterpene saponin from the sponge *Clathria gombawuiensis* | 2015 | Journal of Natural Products |
| Agelamadin F and tauroacidin E, bromopyrrole alkaloids from an Okinawan marine sponge *Agelas* sp | 2015 | Tetrahedron Letters |
| Agelamadins A and B, dimeric bromopyrrole alkaloids from a marine sponge *Agelas* sp | 2014 | Organic Letters |
| Agelamadins C-E, bromopyrrole alkaloids comprising oroidin and 3-hydroxykynurenine from a marine sponge *Agelas* sp | 2014 | Organic Letters |
| Agelamasines A and B, diterpene alkaloids from an Okinawan marine sponge *Agelas* sp. | 2018 | Journal of Natural Medicines |
| Agelasines O-U, new diterpene alkaloids with a 9-N-methyladenine unit from a marine sponge *Agelas* sp | 2012 | Tetrahedron |
| Agelastatin E, agelastatin F, and benzosceptrin C from the marine sponge *Agelas dendromorpha* | 2010 | Journal of Natural Products |
| Alkaloids and polyketides from the South China Sea sponge *Agelas* aff. *Nemoechinata* | 2017 | RSC Advances |
| Allos-hemicalyculin A, a photochemically converted calyculin from the marine sponge *Discodermia calyx* | 2013 | Tetrahedron Letters |
| Alotaketals A and B, sesterterpenoids from the marine sponge *Hamigera* species that activate the cAMP cell signaling pathway | 2009 | Organic Letters |
| Amaranzoles B-F, imidazole-2-carboxy steroids from the marine sponge *Phorbas amaranthus*. C24-N- and C24-O-analogues from a divergent oxidative biosynthesis | 2010 | Journal of Organic Chemistry |
| Amaroxocanes A and B: sulfated dimeric sterols defend the Caribbean coral reef sponge *Phorbas amaranthus* from fish predators | 2009 | Journal of Natural Products |
| Amitorines A and B, nitrogenous diterpene metabolites of *Theonella swinhoei*: isolation, structure elucidation, and asymmetric synthesis | 2016 | Journal of Natural Products |
| Amphimedonoic acid and psammaplysene E, novel brominated alkaloids from *Amphimedon* sp | 2017 | Tetrahedron Letters |
| An acetylenic alkaloid from the calcareous sponge *Leucetta* sp | 2011 | Marine Drugs |
| An antibacterial 9,11-secosterol from a marine sponge *Ircinia* sp | 2014 | Bulletin of the Korean Chemical Society |
| An anti-mycobacterial bisfunctionalized sphingolipid and new bromopyrrole alkaloid from the Indonesian marine sponge *Agelas* sp. | 2017 | Journal of Natural Medicines |
| An unprecedented blue chromophore foundin nature using a “chemistry first” and molecular networking approach: discovery of dactylocyanines A–H | 2017 | Chemistry-A European Journal |
| Analogues of the potent antitumor compound leiodermatolide from a deep-water sponge of the genus *Leiodermatium* | 2017 | Journal of Natural Products |
| Ansellone A, a sesterterpenoid isolated from the nudibranch *Cadlina luteromarginata* and the sponge *Phorbas* sp., activates the cAMP signaling pathway | 2010 | Organic Letters |
| Antibacterial meroterpenoids from the South China Sea sponge *Dysidea* sp | 2016 | Chemical & Pharmaceutical Bulletin |
| Antibacterial secondary metabolites from the cave sponge *Xestospongia* sp | 2012 | Marine Drugs |
| Antichlamydial sterol from the Red Sea sponge *Callyspongia* aff. *implexachlamydial* | 2015 | Planta Medica |
| Antifouling 26,27-cyclosterols from the Vietnamese marine sponge *Xestospongia testudinaria* | 2013 | Journal of Natural Products |
| Antifouling and cytotoxic constituents from the South China Sea sponge *Acanthella cavernosa* | 2012 | Tetrahedron |
| Antifungal bromopyrrole alkaloids from the South China Sea sponge *Agelas* sp | 2016 | Tetrahedron |
| Antifungal cyclic peptides from the marine sponge *Microscleroderma herdmani* | 2012 | Research and Reports in Medicinal Chemistry |
| Antifungal diterpene alkaloids from the Caribbean sponge *Agelas citrina*: unified configurational assignments of agelasidines and agelasines | 2012 | European Journal of Organic Chemistry |
| Anti-helicobacter, antitubercular and cytotoxic activities of scalaranes from the red sea sponge *Hyrtios erectus* | 2018 | Molecules |
| Anti-infective discorhabdins from a deep-water Alaskan sponge of the genus *Latrunculia* | 2010 | Journal of Natural Products |
| Anti-inflammatory cyclopeptides from the marine sponge *Theonella swinhoei* | 2012 | Tetrahedron |
| Antileukemic scalarane sesterterpenoids and meroditerpenoid from *Carteriospongia (Phyllospongia)* sp., induce apoptosis via dual inhibitory effects on topoisomerase II and Hsp90 | 2016 | Scientific Reports |
| Antimalarial activity of axidjiferosides, new β-galactosylceramides from the African sponge *Axinyssa djiferi* | 2013 | Marine Drugs |
| Antimalarial activity of pyrroloiminoquinones from the Australian marine sponge *Zyzzya* sp | 2012 | Journal of Medicinal Chemistry |
| Antimalarial bromotyrosine derivatives from the Australian marine sponge *Hyattella* sp | 2010 | Journal of Natural Products |
| Antimicrobial metabolites from the Paracel Islands sponge *Agelas mauritiana* | 2012 | Journal of Natural Products |
| Anti-mycobacterial alkaloids, cyclic 3-alkyl pyridinium dimers, from the Indonesian marine sponge *Haliclona* sp | 2017 | Bioorganic & Medicinal Chemistry Letters |
| Anti-mycobacterial haliclonadiamine alkaloids from the Okinawan marine sponge *Haliclona* sp. collected at Iriomote Island | 2018 | Phytochemistry Letters |
| Anti-mycobacterium metabolite from marine sponge *Iricinia fusca* | 2017 | British Journal of Pharmaceutical Research |
| Antioxidant benzylidene 2-aminoimidazolones from the Mediterranean sponge *Phorbas topsenti* | 2012 | Tetrahedron |
| Anti-parasitic guanidine and pyrimidine alkaloids from the marine sponge *Monanchora arbuscula* | 2015 | Journal of Natural Products |
| Antiplasmodial activities of homogentisic acid derivative protein kinase inhibitors isolated from a Vanuatu marine sponge *Pseudoceratina* sp | 2009 | Marine Drugs |
| Antipodal crambescin A2 homologues from the marine sponge *Pseudaxinella reticulata*. antifungal structure-activity relationships | 2015 | Journal of Natural Products |
| Antiproliferative activity against human non-small cell lung cancer of two O-alkyl-diglycosylglycerols from the marine sponges *Myrmekioderma dendyi* and *Trikentrion laeve* | 2012 | European Journal of Medicinal Chemistry |
| Antiproliferative homoscalarane sesterterpenes from two Madagascan sponges | 2013 | Bioorganic & Medicinal Chemistry |
| Antiproliferative scalarane-based metabolites from the Red Sea sponge *Hyrtios erectus* | 2016 | Marine Drugs |
| Antiprotozoal linear furanosesterterpenoids from the marine sponge *Ircinia oros* | 2017 | Journal of Natural Products |
| Antiprotozoal steroidal saponins from the marine sponge *Pandaros acanthifolium* | 2010 | Journal of Natural Products |
| Antitrypanosomal cyclic polyketide peroxides from the Australian marine sponge *Plakortis* sp | 2010 | Journal of Natural Products |
| Antitumour polyether macrolides: four new halichondrins from the New Zealand deep-water marine sponge *Lissodendoryx* sp | 2009 | Bioorganic & Medicinal Chemistry |
| Aplysfistularine: a novel dibromotyrosine derivative isolated from *Aplysina fistularis* | 2012 | Quimica Nova |
| Aplysinellamides A-C, bromotyrosine-derived metabolites from an Australian *Aplysinella* sp. marine sponge | 2014 | Journal of Natural Products |
| ApoE secretion modulating bromotyrosine derivative from the Australian marine sponge *Callyspongia* sp | 2014 | Bioorganic & Medicinal Chemistry Letters |
| ApoE secretion modulatingbromotyrosinederivative from the Australian marine sponge *Callyspongia* sp | 2014 | Bioorganic & Medicinal Chemistry Letters |
| Araiosamines A-D: tris-bromoindole cyclic guanidine alkaloids from the marine sponge *Clathria (Thalysias) araiosa* | 2011 | Journal of Organic Chemistry |
| Aromatic cyclic peroxides and related keto-compounds from the *Plakortis* sp. component of a sponge consortium | 2009 | Journal of Natural Products |
| Asteropsin A: an unusual cystine-crosslinked peptide from porifera enhances neuronal Ca2+ influx | 2013 | Biochimica et Biophysica ACTA |
| Asteropsins B-D, sponge-derived knottins with potential utility as a novel scaffold for oral peptide drugs | 2014 | Biochimica et Biophysica ACTA |
| Atkamine: a new pyrroloiminoquinone scaffold from the cold water Aleutian Islands *Latrunculia* sponge | 2013 | Organic Letters |
| Aurantoside J: a new tetramic acid glycoside from *Theonella swinhoei*. Insights into the antifungal potential of aurantosides | 2011 | Marine Drugs |
| Aurantoside K, a new antifungal tetramic acid glycoside from a Fijian marine sponge of the genus *Melophlus* | 2012 | Marine Drugs |
| Australian marine sponge alkaloids as a new class of glycine-gated chloride channel receptor modulator | 2013 | Bioorganic & Medicinal Chemistry Letters |
| Axinellin A and B: two new pyrrolactam alkaloids from *Axinella* sp | 2017 | Chemistry of Natural Compounds |
| Axiphenylalaninium and axityrosinium, modified amino acids from the Mediterranean marine sponge *Axinella polypoides* | 2009 | Journal of Natural Products |
| Axiriabilines A-D, uncommon nitrogenous eudesmane-type sesquiterpenes from the Hainan sponge *Axinyssa variabilis* | 2017 | Tetrahedron |
| Baculiferins A-O, O-sulfated pyrrole alkaloids with anti-HIV-1 activity, from the Chinese marine sponge *Iotrochota baculifera* | 2010 | Bioorganic & Medicinal Chemistry |
| Balibalosides, an original family of glycosylated sesterterpenes produced by the Mediterranean sponge *Oscarella balibaloi* | 2013 | Marine Drugs |
| Benzosceptrin C, a new dimeric bromopyrrole alkaloid from sponge *Agelas* sp | 2009 | Tetrahedron Letters |
| Benzosceptrins A and B with a unique benzocyclobutane skeleton and nagelamide S and T from Pacific sponges | 2009 | Organic Letters |
| Biakamides A-D, unique polyketides from a marine sponge, act as selective growth inhibitors of tumor cells adapted to nutrient starvation | 2017 | Journal of Organic Chemistry |
| Bicyclic C21 terpenoids from the marine sponge *Clathria compressa* | 2012 | Journal of Natural Products |
| Bifunctionalized amphilectane diterpenes from the sponge *Stylissa cf. massa* | 2012 | Journal of Natural Products |
| Bioactive alkaloids from the Red Sea marine Verongid sponge *Pseudoceratina arabica* | 2015 | Tetrahedron |
| Bioactive bromotyrosine-derived alkaloids from the Polynesian sponge *Suberea ianthelliformis* | 2018 | Marine Drugs |
| Bioactive compounds from the red sea marine sponge *Hyrtios* species | 2013 | Marine Drugs |
| Bioactive cycloperoxides isolated from the Puerto Rican sponge *Plakortis halichondrioides* | 2010 | Journal of Natural Products |
| Bioactive diterpene derivatives from the marine sponge *Spongionella* sp | 2009 | Journal of Natural Products |
| Bioactive guanidine alkaloids from two Caribbean marine sponges | 2009 | Journal of Natural Products |
| Bioactive hydantoin alkaloids from the red sea marine sponge *Hemimycale arabica* | 2015 | Marine Drugs |
| Bioactive indole derivatives from the South Pacific marine sponges *Rhopaloeides odorabile* and *Hyrtios* sp | 2011 | Marine Drugs |
| Bioactive isoquinolinequinone alkaloids from the South China Sea nudibranch *Jorunna funebris* and its sponge-prey *Xestospongia* sp | 2016 | Future Medicinal Chemistry |
| Bioactive lipids from the sponge *Spirastrella abata* | 2012 | Bioorganic & Medicinal Chemistry Letters |
| Bioactive metabolites from the sponge *Suberea* sp | 2010 | Chemistry & Biodiversity |
| Bioactive polyhydroxylated sterols from the marine sponge *Haliclona crassiloba* | 2013 | Steroids |
| Bioactive scalaranes from the Thai sponge *Hyrtios gumminae* | 2009 | Journal of Natural Products |
| Bioactive secondary metabolites from the Red Sea marine Verongid sponge *Suberea* species | 2015 | Marine Drugs |
| Bioactive sesquiterpene quinols and quinones from the marine sponge *Dysidea avara* | 2015 | RSC Advances |
| Bioactive sesterterpenoids from a Korean sponge *Monanchora* sp | 2013 | Journal of Natural Products |
| Bioactive sulfated sesterterpene alkaloids and sesterterpene sulfates from the marine sponge *Fasciospongia* sp | 2009 | Journal of Natural Products |
| Bioactive terpenes from *Spongia officinalis* | 2011 | Journal of Natural Products |
| Biostructural features of additional jasplakinolide (jaspamide) analogues | 2011 | Journal of Natural Products |
| Biscembranoids from the marine sponge *Petrosia nigricans* | 2013 | Natural Product Communications |
| Bis-sesquiterpene from the marine sponge *Dysidea fragilis* | 2016 | Natural Product Communications |
| Brominated aliphatic hydrocarbons and sterols from the sponge *Xestospongia testudinaria* with their bioactivities | 2011 | Chemistry and Physics of Lipids |
| Brominated arginine-derived alkaloids from the Red Sea sponge *Suberea mollis* | 2011 | Journal of Natural Products |
| Brominated polyunsaturated lipids and their stereochemistry from the Chinese marine sponge *Xestospongia testudinaria* | 2011 | Tetrahedron |
| Brominated polyunsaturated lipids from the Chinese sponge *Xestospongia testudinaria* as a new class of pancreatic lipase inhibitors | 2014 | European Journal of Medicinal Chemistry |
| Brominated polyunsaturated lipids with protein tyrosine phosphatase-1B inhibitory activity from Chinese marine sponge *Xestospongia testudinaria* | 2015 | Journal of Asian Natural Products Research |
| Bromine-containing alkaloids from the marine sponge *Penares* sp | 2012 | Tetrahedron Letters |
| Bromopyrrole alkaloid inhibitors of the proteasome isolated from a *Dictyonella* sp. marine sponge collected at the Amazon River mouth | 2018 | Journal of Natural Products |
| Bromopyrrole alkaloids from a marine sponge *Agelas* sp | 2014 | Chemical & Pharmaceutical Bulletin |
| Bromopyrrole alkaloids from marine sponges of the genus *Agelas* | 2009 | Journal of Natural Products |
| Bromopyrrole alkaloids from the sponge *Agelas kosrae* | 2018 | Marine Drugs |
| Bromopyrrole alkaloids with the inhibitory effects against the biofilm formation of gram negative bacteria | 2018 | Marine Drugs |
| Bromotheoynic acid, a brominated acetylenic acid from the marine sponge *Theonella swinhoei* | 2013 | Natural Product Research |
| Bromotyrosine alkaloids from the Australian marine sponge *Pseudoceratina verrucosa* | 2013 | Journal of Natural Products |
| Bromotyrosine alkaloids with acetylcholinesterase inhibitory activity from the Thai sponge *Acanthodendrilla* sp | 2015 | Natural Product Communications |
| Bromotyrosine-derived alkaloids from the Caribbean sponge *Aplysina lacunosa* | 2015 | Beilstein Journal of Organic Chemistry |
| Bromotyrosine-derived metabolites from an Indonesian marine sponge in the family Aplysinellidae (Order Verongiida) | 2016 | Bioorganic & Medicinal Chemistry Letters |
| C29 sterols with a cyclopropane ring at C-25 and 26 from the Vietnamese marine sponge *Ianthella* sp. and their anticancer properties | 2009 | Bioorganic & Medicinal Chemistry Letters |
| Cacolides: sesterterpene butenolides from a southern Australian marine sponge, *Cacospongia* sp | 2018 | Marine Drugs |
| Callyaerins A-F and H, new cytotoxic cyclic peptides from the Indonesian marine sponge *Callyspongia aerizusa* | 2010 | Bioorganic & Medicinal Chemistry |
| Callyaerins from the marine sponge *Callyspongia aerizusa*: cyclic peptides with antitubercular activity | 2015 | Journal of Natural Products |
| Callyazepin and (3R)-methylazacyclodecane, nitrogenous macrocycles from a *Callyspongia* sp. sponge | 2016 | Journal of Natural Products |
| Callyptide A, a new cytotoxic peptide from the Red Sea marine sponge *Callyspongia* species | 2016 | Natural Product Research |
| Callyspongiamides A and B, sterol O-acyltransferase inhibitors, from the Indonesian marine sponge *Callyspongia* sp | 2018 | Bioorganic & Medicinal Chemistry Letters |
| Callyspongidic acids: amphiphilic diacids from the tropical eastern Pacific sponge *Callyspongia* cf. *californica* | 2018 | Journal of Natural Products |
| Callyspongiolide, a cytotoxic macrolide from the marine sponge *Callyspongia* sp | 2014 | Organic Letters |
| Callyspongisines A-D: bromopyrrole alkaloids from an Australian marine sponge, *Callyspongia* sp | 2014 | Organic & Biomolecular Chemistry |
| Calyxamides A and B, cytotoxic cyclic peptides from the marine sponge *Discodermia calyx* | 2012 | Journal of Natural Products |
| Can stereoclusters separated by two methylene groups be related by dft studies? the case of the cytotoxic meroditerpenes halioxepines | 2018 | Journal of Natural Products |
| Carteriosulfonic acids A-C, GSK-3β inhibitors from a *Carteriospongia* sp | 2009 | Journal of Natural Products |
| Carteritins A and B, cyclic heptapeptides from the marine sponge *Stylissa carteri* | 2016 | Tetrahedron Letters |
| Celebesides A-C and theopapuamides B-D, depsipeptides from an Indonesian sponge that inhibit HIV-1 entry | 2009 | Journal of Organic Chemistry |
| Cell differentiation inducers from a marine sponge *Biemna* sp | 2011 | Tetrahedron |
| Cellular localization of clathridimine, an antimicrobial 2-aminoimidazole alkaloid produced by the Mediterranean calcareous sponge *Clathrina clathrus* | 2010 | Journal of Natural Products |
| Ceratinadins A-C, new bromotyrosine alkaloids from an Okinawan marine sponge *Pseudoceratina* sp | 2010 | Bioorganic & Medicinal Chemistry Letters |
| Ceratinadins E and F, new bromotyrosine alkaloids from an Okinawan marine sponge *Pseudoceratina* sp | 2018 | Marine Drugs |
| Ceratodictyols, 1-glyceryl ethers from the red alga-sponge association *Ceratodictyon spongiosum*/*Haliclona cymaeformis* | 2009 | Journal of Natural Products |
| Cerebrosides from a Far-Eastern glass sponge *Aulosaccus* sp | 2015 | Lipids |
| Ceylonamides A-F, nitrogenous spongian diterpenes that inhibit RANKL-induced osteoclastogenesis, from the marine sponge *Spongia ceylonensis* | 2016 | Journal of Natural Products |
| Ceylonins A-F, spongian diterpene derivatives that inhibit RANKL-induced formation of multinuclear osteoclasts, from the marine sponge *Spongia ceylonensis* | 2017 | Journal of Natural Products |
| Ceylonins G–I: spongian diterpenes from the marine sponge *Spongia ceylonensis* | 2017 | Journal of Natural Medicines |
| Chagosendines A - C, new metal complexes of imidazole alkaloids from the calcareous sponge *Leucetta chagosensis* | 2017 | Chemistry & Biodiversity |
| Chalinulasterol, a chlorinated steroid disulfate from the Caribbean sponge *Chalinula molitba*. Evaluation of its role as PXR receptor modulator | 2012 | Marine Drugs |
| Characterization and anti-inflammatory effects of iodinated acetylenic acids isolated from the marine sponges *Suberites mammilaris* and *Suberites japonicus* | 2013 | Journal of Natural Products |
| Chemical constituents of the deep reef Caribbean sponges *Plakortis angulospiculatus* and *Plakortis halichondrioides* and their anti-inflammatory activities | 2010 | Journal of Natural Products |
| Chemical examination of the sponge *Phycopsis* sp | 2009 | Chemical & Pharmaceutical Bulletin |
| Chemistry and selective tumor cell growth inhibitory activity of polyketides from the South China Sea sponge *Plakortis* sp | 2017 | Marine Drugs |
| Chemistry of renieramycins, part 10: structure of renieramycin V, a novel renieramycin marine natural product having a sterol ether at C-14 position | 2012 | Heterocycles |
| Chemistry of renieramycins. 16. structure of 7-demethylrenieramycin o (= 14α-hydroxyrenieramycin S) from blue sponge, *Xestospongia* sp | 2017 | Heterocycles |
| Chemistry of renieramycins. Part 13: isolation and structure of stabilized renieramycin type derivatives, renieramycins W-Y, from Philippine blue sponge *Xestospongia* sp., pretreated with potassium cyanide | 2012 | Tetrahedron |
| Chemistry of renieramycins. Part 7: renieramycins T and U, novel renieramycin-ecteinascidin hybrid marine natural products from Thai sponge *Xestospongia* sp | 2009 | Tetrahedron Letters |
| Chlorinated thiazole-containing polyketide-peptides from the Caribbean sponge *Smenospongia conulosa*: structure elucidation on microgram scale | 2016 | European Journal of Organic Chemistry |
| Chloromethylhalicyclamine B, a marine-derived protein kinase CK1δ/ ε inhibitor | 2016 | Journal of Natural Products |
| Cinanthrenol A, an estrogenic steroid containing phenanthrene nucleus, from a marine sponge *Cinachyrella* sp | 2014 | Organic Letters |
| Citronamides A and B, tetrapeptides from the Australian sponge *Citronia astra* | 2009 | Journal of Natural Products |
| Clavatadines C-E, guanidine alkaloids from the Australian sponge *Suberea clavata* | 2009 | Journal of Natural Products |
| Colony-wise analysis of a *Theonella swinhoei* marine sponge with a yellow interior permitted the isolation of theonellamide I | 2018 | Journal of Natural Products |
| Configurational assignment of cyclic peroxy metabolites provides an insight into their biosynthesis: isolation of plakortolides, seco-plakortolides, and plakortones from the Australian Marine sponge *Plakinastrella clathrata* | 2011 | Journal of Natural Products |
| Conicasterol E, a small heterodimer partner sparing farnesoid X receptor modulator endowed with a pregnane X receptor agonistic activity, from the marine sponge *Theonella swinhoei* | 2012 | Journal of Medicinal Chemistry |
| Constituents from *Ircinia echinata* and their antiproliferative effect on six human cancer cell strains | 2017 | Letters in Organic Chemistry |
| Coscinolactams A and B: new nitrogen-containing sesterterpenoids from the marine sponge *Coscinoderma mathewsi* exerting anti-inflammatory properties | 2009 | Tetrahedron |
| Crellasterones A and B: a-norsterol derivatives from the New Caledonian sponge *Crella incrustans* | 2017 | Marine Drugs |
| Cyclic 3-alkyl pyridinium alkaloid monomers from a New Zealand *Haliclona* sp. marine sponge | 2013 | Journal of Natural Products |
| Cyclic bis-1,3-dialkylpyridiniums from the sponge *Haliclona* sp | 2012 | Marine Drugs |
| Cyclic cystine-bridged peptides from the marine sponge *Clathria basilana* induce apoptosis in tumor cells and depolarize the bacterial cytoplasmic membrane | 2017 | Journal of Natural Products |
| Cyclic peroxides from a two-sponge association of *Plakortis communis*-*Agelas mauritiana* | 2013 | Natural Product Communications |
| Cyclopeptides from the sponge *Stylissa flabelliformis* | 2018 | Journal of Natural Products |
| Cyclotheonellazoles A-C, potent protease inhibitors from the marine sponge *Theonella aff. swinhoei* | 2017 | Journal of Natural Products |
| Cytotoxic 5-hydroxyindole alkaloids from the marine sponge *Scalarispongia* sp | 2013 | Journal of Heterocyclic Chemistry |
| Cytotoxic 5α,8α-epidioxy sterols from the marine sponge *Monanchora* sp | 2015 | Archives of Pharmacal Research |
| Cytotoxic aaptamine derivatives from the South China Sea sponge *Aaptos aaptos* | 2014 | Journal of Natural Products |
| Cytotoxic aaptamines from Malaysian *Aaptos aaptos* | 2009 | Marine Drugs |
| Cytotoxic activity of alkyl benzoate and fatty acids from the red sea sponge *Hyrtios erectus* | 2018 | Natural Product Research |
| Cytotoxic activity of rearranged drimane meroterpenoids against colon cancer cells via down-regulation of β-catenin expression | 2015 | Journal of Natural Products |
| Cytotoxic and haemolytic steroidal glycosides from the Caribbean sponge *Pandaros acanthifolium* | 2011 | Steroids |
| Cytotoxic and protein kinase inhibiting nakijiquinones and nakijiquinols from the sponge *Dactylospongia metachromia* | 2014 | Journal of Natural Products |
| Cytotoxic anomoian b and aplyzanzine b, new bromotyrosine alkaloids from Indonesian sponges | 2017 | ACS Omega |
| Cytotoxic C21 and C22 Terpenoid-derived metabolites from the sponge *Ircinia* sp | 2011 | Journal of Natural Products |
| Cytotoxic ceramides from the Red Sea sponge *Spheciospongia vagabunda* | 2015 | Medicinal Chemistry Research |
| Cytotoxic components from the Xisha sponge *Fascaplysinopsis reticulata* | 2018 | Natural Product Research |
| Cytotoxic compounds from the Saudi Red Sea sponge *Xestospongia testudinaria* | 2016 | Marine Drugs |
| Cytotoxic cyclic depsipeptides from the Australian marine sponge *Neamphius huxleyi* | 2012 | Journal of Natural Products |
| Cytotoxic diterpenoid pseudodimers from the Korean sponge *Phorbas gukulensis* | 2013 | Journal of Natural Products |
| Cytotoxic drimane meroterpenoids from the Indonesian marine sponge *Dactylospongia elegans* | 2017 | Phytochemistry Letters |
| Cytotoxic effects of three new metabolites from Red Sea marine sponge, *Petrosia* sp | 2014 | Environmental Toxicology and Pharmacology |
| Cytotoxic glycosylated fatty acid amides from a *Stelletta* sp. marine sponge | 2015 | Journal of Natural Products |
| Cytotoxic guanidine alkaloids from a French Polynesian *Monanchora* n. sp. sponge | 2016 | Journal of Natural Products |
| Cytotoxic isomalabaricane derivatives and a monocyclic triterpene glycoside from the sponge *Rhabdastrella globostellata* | 2010 | Journal of Natural Products |
| Cytotoxic linear acetylenes from a marine sponge *Pleroma* sp | 2015 | Tetrahedron |
| Cytotoxic monocarbocyclic sesterterpenoids from a marine sponge *luffariella* sp | 2015 | Bulletin of the Chemical Society of Japan |
| Cytotoxic neviotane triterpene‑type from the red sea sponge *Siphonochalina siphonella* | 2014 | Pharmacognosy Magazine |
| Cytotoxic petrosiacetylenes from the marine sponge *Petrosia* sp | 2013 | Lipids |
| Cytotoxic phyllactone analogs from the marine sponge *Phyllospongia papyrecea* | 2017 | Medicinal Chemistry Research |
| Cytotoxic plakortides from the Brazilian Marine sponge *Plakortis angulospiculatus* | 2015 | Journal of Natural Products |
| Cytotoxic polyacetylenes from a Formosan marine sponge *Callyspongia* sp | 2014 | Bulletin of the Chemical Society of Japan |
| Cytotoxic polyacetylenes from the Red Sea sponge *Siphonochalina siphonella* | 2014 | Zeitschrift fur Naturforschung Section C-A Journal of Biosciences |
| Cytotoxic polyacetylenes related to petroformyne-1 from the marine sponge *Petrosia* sp | 2009 | Tetrahedron |
| Cytotoxic polyketide derivatives from the South China Sea sponge *Plakortis simplex* | 2013 | Journal of Natural Products |
| Cytotoxic psammaplysin analogues from a *Suberea* sp. marine sponge and the role of the spirooxepinisoxazoline in their activity | 2013 | Journal of Natural Products |
| Cytotoxic scalarane sesterterpenes from a Korean marine sponge *Psammocinia* sp | 2013 | Bioorganic & Medicinal Chemistry Letters |
| Cytotoxic scalarane sesterterpenoids from a marine sponge *Hippospongia* sp | 2013 | Natural Product Communications |
| Cytotoxic scalarane sesterterpenoids from the South China Sea sponge *Carteriospongia foliascens* | 2015 | Organic & Biomolecular Chemistry |
| Cytotoxic scalarane-type sesterterpenes from the Saudi Red Sea sponge *Hyrtios erectus* | 2016 | Journal of Asian Natural Products Research |
| Cytotoxic sesterterpenes from Thai marine sponge *Hyrtios erectus* | 2018 | Marine Drugs |
| Cytotoxic sesterterpenoids from a sponge *Hippospongia* sp | 2012 | Marine Drugs |
| Cytotoxic sesterterpenoids isolated from the marine sponge *Scalarispongia* sp | 2014 | International Journal of Molecular Sciences |
| Daedophamide, a cytotoxic cyclodepsipeptide from a *Daedalopelta* sp. sponge collected in Indonesia | 2017 | Journal of Natural Products |
| Darwinolide, a new diterpene scaffold that inhibits methicillin-resistant Staphylococcus aureus biofilm from the Antarctic sponge *Dendrilla membranosa* | 2016 | Organic Letters |
| Debromokeramadine from the marine sponge *Agelas cf. mauritiana*: isolation and short regioselective and flexible synthesis | 2015 | Tetrahedron |
| Dehydroconicasterol and aurantoic acid, a chlorinated polyene derivative, from the Indonesian sponge *Theonella swinhoei* | 2009 | Journal of Natural Products |
| Denigrins A-C: new antitubercular 3,4-diarylpyrrole alkaloids from *Dendrilla nigra* | 2014 | Natural Product Research |
| Densanins A and B, new macrocyclic pyrrole alkaloids isolated from the marine sponge *Haliclona densaspicula* | 2012 | Organic Letters |
| Desulfohaplosamate, a new phosphate-containing steroid from Dasychalina sp., is a selective cannabinoid CB2 receptor ligand | 2011 | Steroids |
| Diacarperoxide S, new norterpene cyclic peroxide from the sponge *Diacarnus megaspinorhabdosa* | 2012 | Natural Product Communications |
| Dibromopyrrole alkaloids from the marine sponge *Acanthostylotella* sp | 2009 | Natural Product Communications |
| Dibromotyrosine and histamine derivatives from the tropical marine sponge *Aplysina* sp | 2010 | Natural Product Communications |
| Dictazoles: potential vinyl cyclobutane biosynthetic precursors to the dictazolines | 2010 | Journal of Organic Chemistry |
| Dictyoneolone, a B/C ring juncture-defused steroid from a *Dictyonella* sp. sponge | 2018 | Tetrahedron Letters |
| Dihydrohymenialdisines, new pyrrole-2-aminoimidazole alkaloids from the marine sponge *Cymbastela cantharella* | 2011 | Tetrahedron Letters |
| Diplopuupehenone, a new unsymmetrical puupehenone-related dimer from the marine sponge *Dysidea* sp | 2011 | Tetrahedron Letters |
| Discorhabdins from the Korean Marine sponge *Sceptrella* sp | 2010 | Journal of Natural Products |
| Discorhabdins revisited: cytotoxic alkaloids from southern Australian marine sponges of the genera *Higginsia* and *Spongosorites* | 2009 | Journal of Natural Products |
| Discovery and synthesis of namalide reveals a new anabaenopeptin scaffold and peptidase inhibitor | 2012 | Journal of Medicinal Chemistry |
| Dispacamide E and other bioactive bromopyrrole alkaloids from two Indonesian marine sponges of the genus *Stylissa* | 2015 | Natural Product Research |
| Diterpene formamides from the tropical marine sponge *Cymbastela hooperi* and their antimalarial activity in vitro | 2009 | Journal of Natural Products |
| Donnazoles A and B from *Axinella donnani* sponge: very close derivatives from the postulated intermediate ‘pre-axinellamine’ | 2012 | Tetrahedron Letters |
| Dragmacidins G and H, bisindole alkaloids tethered by a guanidino ethylthiopyrazine moiety, from a *Lipastrotethya* sp. marine sponge | 2016 | Journal of Natural Products |
| Dragmacidol A and dragmacidolide A from the Australian marine sponge *Dragmacidon australe* | 2015 | Tetrahedron |
| Dragmacidoside: a new nucleoside from the Red Sea sponge *Dragmacidon coccinea* | 2014 | Natural Product Research |
| Dysiarenone, a dimeric c21 meroterpenoid with inhibition of cox-2 expression from the marine sponge *Dysidea arenaria* | 2018 | Organic Letters |
| Dysidaminones A–M, cytotoxic and NF-kB inhibitory sesquiterpene aminoquinones from the South China Sea sponge *Dysidea fragilis* | 2014 | RSC Advances |
| Dysidavarones A-D, new sesquiterpene quinones from the marine sponge *Dysidea avara* | 2012 | Organic Letters |
| Dysideamine, a new sesquiterpene aminoquinone, protects hippocampal neuronal cells against iodoacetic acid-induced cell death | 2009 | Bioorganic & Medicinal Chemistry |
| Dysideanones A-C, unusual sesquiterpene quinones from the South China Sea sponge *Dysidea avara* | 2014 | Journal of Natural Products |
| Dysidinoid A, an unusual meroterpenoid with anti-MRSA activity from the South China sea sponge *Dysidea* sp | 2014 | Molecules |
| Dysifragilones A-C, unusual sesquiterpene aminoquinones and inhibitors of NO production from the South China Sea sponge *Dysidea fragilis* | 2015 | European Journal of Organic Chemistry |
| Dysiherbols A-C and dysideanone e, cytotoxic and NF-κB Inhibitory tetracyclic meroterpenes from a *Dysidea* sp. marine sponge | 2016 | Journal of Natural Products |
| Dysivillosins A-D, unusual anti-allergic meroterpenoids from the marine sponge *Dysidea villosa* | 2017 | Scientific Reports |
| Ecionines A and B, two new cytotoxic pyridoacridine alkaloids from the Australian marine sponge, *Ecionemia geodides* | 2010 | Tetrahedron |
| Ehrenasterol and biemnic acid; new bioactive compounds from the Red Sea sponge *Biemna ehrenbergi* | 2015 | Phytochemistry Letters |
| Enantiodivergence in the biosynthesis of bromotyrosine alkaloids from sponges? | 2017 | Journal of Natural Products |
| Endoperoxide polyketides from a Chinese *Plakortis simplex*: further evidence of the impact of stereochemistry on antimalarial activity of simple 1,2-dioxanes | 2014 | Bioorganic & Medicinal Chemistry |
| Epi-leptosphaerin: a new l-isoascorbic acid derivative from marine sponges | 2015 | Natural Product Sciences |
| Eryloside W, a triterpenoid saponin from the sponge *Dictyonella marsilii* | 2015 | Phytochemistry Letters |
| Erylosides F8,V1-V3, and W-W2-New triterpene oligoglycosides from the Carribean sponge *Erylus goffrilleri* | 2017 | Carbohydrate Research |
| Erylusamides: novel atypical glycolipids from *Erylus cf. deficiens* | 2016 | Marine Drugs |
| Euryjanicin A: a new cycloheptapeptide from the Caribbean marine sponge *Prosuberites laughlini* | 2009 | Tetrahedron Letters |
| Euryjanicins E-G, poly-phenylalanine, and poly-proline cyclic heptapeptides from the Caribbean sponge *Prosuberites laughlini* | 2013 | Tetrahedron |
| Euryspongins A-C, three new unique sesquiterpenes from a marine sponge *Euryspongia* sp | 2013 | Bioorganic & Medicinal Chemistry Letters |
| Evaluation of pyridoacridine alkaloids in a zebrafish phenotypic assay | 2010 | Marine Drugs |
| Evaluation of the antiproliferative activity of diterpene isonitriles from the sponge *Pseudoaxinella flava* in apoptosis-sensitive and apoptosis-resistant cancer cell lines | 2011 | Journal of Natural Products |
| Exploring the sponge consortium *Plakortis symbiotica*-*Xestospongia deweerdtae* as a potential source of antimicrobial compounds and probing the pharmacophore for antituberculosis activity of smenothiazole A by diverted total synthesis | 2017 | Journal of Natural Products |
| Fascioquinols A-F: bioactive meroterpenes from a deep-water southern Australian marine sponge, *Fasciospongia* sp | 2011 | Tetrahedron |
| Fasciospyrinadine, a novel sesquiterpene pyridine alkaloid from a Guangxi sponge *Fasciospongia* sp | 2013 | Journal of Asian Natural Products Research |
| Fatty acids from a glass sponge *Aulosaccus* sp. Occurrence of new cyclopropane-containing and methyl-branched acids | 2017 | Lipids |
| Faulknerynes A-C from a Bahamian sponge *Diplastrella* sp.: stereoassignment by critical application of two exciton coupled CD methods | 2011 | Journal of Organic Chemistry |
| Fibrosterol sulfates from the Philippine sponge *Lissodendoryx (Acanthodoryx) fibrosa*: sterol dimers that inhibit PKCζ | 2009 | Journal of Organic Chemistry |
| Flabelliferins A and B, sesterterpenoids from the south Pacific sponge *Carteriospongia flabellifera* | 2012 | Journal of Natural Products |
| Formamidobisabolene-based derivatives from a sponge *Axinyssa* sp | 2014 | Tetrahedron |
| Formamido-diterpenes from the South China Sea sponge *Acanthella cavernosa* | 2012 | Marine Drugs |
| Four cytotoxic spongian diterpenes from the sponge *Dysidea cf. arenaria* | 2016 | Chemical & Pharmaceutical Bulletin |
| Four new 6-oxy purine alkaloids from the South China Sea sponge, *Haliclona cymaeformis* | 2017 | Journal of Ocean University of China |
| Four sesquiterpenes isolated from a marine sponge *Topsentia* species | 2014 | Journal of the Korean Magnetic Resonance Society |
| Franklinolides A-C from an Australian marine sponge complex: phosphodiesters strongly enhance polyketide cytotoxicity | 2010 | Angewandte Chemie-International Editio |
| From anti-fouling to biofilm inhibition: new cytotoxic secondary metabolites from two Indonesian *Agelas* sponges | 2010 | Bioorganic & Medicinal Chemistry |
| FTICR-MS and LC-UV/MS-SPE-NMR applications for the rapid dereplication of a crude extract from the sponge *Ianthella flabelliformis* | 2009 | Journal of Natural Products |
| Fulvynes, antimicrobial polyoxygenated acetylenes from the Mediterranean sponge *Haliclona fulva* | 2012 | Tetrahedron |
| Furanosesterterpenes from the Guangxi sponge *Biemna fortis Topsent* | 2018 | Biochemical Systematics and Ecology |
| Furanoterpene diversity and variability in the marine sponge *Spongia officinalis*, from untargeted LC-MS/MS metabolomic profiling to furanolactam derivatives | 2017 | Metabolites |
| Further brominated polyacetylenes with pancreatic lipase inhibitory activity from Chinese marine sponge *Xestospongia testudinaria* | 2017 | Journal of Asian Natural Products Research |
| Further investigation of the Mediterranean sponge *Axinella polypoides*: isolation of a new cyclonucleoside and a new betaine | 2012 | Marine Drugs |
| Further study on Penares sp. from Vietnamese waters: minor lanostane and nor-lanostane triterpenes | 2015 | Steroids |
| Globostelletins A-I, cytotoxic isomalabaricane derivatives from the marine sponge *Rhabdastrella globostellata* | 2010 | Bioorganic & Medicinal Chemistry |
| Globostelletins J-S (sic), isomalabaricanes with unusual cyclopentane sidechains from the marine sponge *Rhabdastrella globostellata* | 2012 | Tetrahedron |
| Gombamide A, a cyclic thiopeptide from the sponge *Clathria gombawuiensis* | 2013 | Journal of Natural Products |
| Gombaspiroketals A-C, sesterterpenes from the sponge *Clathria gombawuiensis* | 2014 | Organic Letters |
| Gracilioethers A-C, antimalarial metabolites from the marine sponge *Agelas gracilis* | 2009 | Journal of Organic Chemistry |
| Gracilioethers E-J, new oxygenated polyketides from the marine sponge *Plakinastrella mamillaris* | 2012 | Tetrahedron |
| Gukulenins A and B, cytotoxic tetraterpenoids from the marine sponge *Phorbas gukulensis* | 2010 | Journal of Natural Products |
| Hainanerectamines A-C, alkaloids from the Hainan sponge *Hyrtios erecta* | 2014 | Marine Drugs |
| Halenaquinone derivatives from tropical marine sponge *xestospongia* sp | 2012 | Heterocycles |
| Halichonadins G-J, new sesquiterpenoids from a sponge *Halichondria* sp | 2011 | Tetrahedron Letters |
| Halichonadins K and L, new dimeric sesquiterpenoids from a sponge *Halichondria* sp | 2012 | Organic Letters |
| Halichonadins M-Q, sesquiterpenes from an Okinawan marine sponge *Halichondria* sp | 2015 | Heterocycles |
| Halichonines A, B, and C, novel sesquiterpene alkaloids from the marine sponge *Halichondria okadai Kadota* | 2011 | Chemical Communications |
| Haliclocyclin C, a new monomeric 3-alkyl pyridinium alkaloid from the arctic marine sponge *Haliclona viscosa* | 2011 | Zeitschrift fur Naturforschung Section B-A Journal of Chemical Sciences |
| Halicloic acids A and B isolated from the marine sponge *Haliclona* sp. collected in the Philippines inhibit indoleamine 2,3-dioxygenase | 2012 | Journal of Natural Products |
| Haliclonacyclamines, tetracyclic alkylpiperidine alkaloids, as anti-dormant mycobacterial substances from a marine sponge of *Haliclona* sp | 2009 | Chemical & Pharmaceutical Bulletin |
| Haliclonadiamine derivatives and 6-epi-monanchorin from the marine sponge *Halichondria panicea* collected at Iriomote Island | 2016 | Journal of Natural Products |
| Haliclonin A, a new macrocyclic diamide from the sponge *Haliclona* sp | 2009 | Organic Letters |
| Halioxepine, a new meroditerpene from an Indonesian sponge *Haliclona* sp | 2011 | Chemical & Pharmaceutical Bulletin |
| Haliscosamine: a new antifungal sphingosine derivative from the Moroccan marine sponge *Haliclona viscosa* | 2013 | SpringerPlus |
| Halisphingosines A and B, modified sphingoid bases from Haliclona tubifera. Assignment of configuration by circular dichroism and van't Hoff's principle of optical superposition | 2013 | Journal of Natural Products |
| Halistanol sulfates I and J, new SIRT1-3 inhibitory steroid sulfates from a marine sponge of the genus *Halichondria* | 2018 | The Journal of Antibiotics |
| Hamigerans R and S: nitrogenous diterpenoids from the New Zealand marine sponge *Hamigera tarangaensis* | 2018 | Journal of Natural Products |
| Heteroaromatic alkaloids, nakijinamines, from a sponge *Suberites* sp | 2012 | Tetrahedron |
| Heterofibrins: inhibitors of lipid droplet formation from a deep-water southern Australian marine sponge, *Spongia (Heterofibria)* sp | 2010 | Organic & Biomolecular Chemistry |
| Hippolachnin A, a new antifungal polyketide from the South China Sea sponge *Hippospongia lachne* | 2013 | Organic Letters |
| Hippolides A-H, acyclic manoalide derivatives from the marine sponge *Hippospongia lachne* | 2011 | Journal of Natural Products |
| Homophymines B-E and A1-E1, a family of bioactive cyclodepsipeptides from the sponge *Homophymia* sp | 2009 | Organic & Biomolecular Chemistry |
| Hybrid pyrrole-imidazole alkaloids from the sponge *Agelas sceptrum* | 2016 | Journal of Natural Products |
| Hymerhabdrin A, a novel diterpenoid with antifouling activity from the intertidal sponge *Hymerhabdia* sp | 2017 | The Journal of Antibiotics |
| Hyrtimomines A-C, new heteroaromatic alkaloids from a sponge *Hyrtios* sp | 2013 | Organic Letters |
| Hyrtimomines D and E, bisindole alkaloids from a marine sponge *Hyrtios* sp | 2013 | Tetrahedron Letters |
| Hyrtimomines, indole alkaloids from Okinawan marine sponges *Hyrtios* spp | 2014 | Tetrahedron |
| Hyrtinadines C and D, new azepinoindole-type alkaloids from a marine sponge *Hyrtios* sp | 2016 | Chemical & Pharmaceutical Bulletin |
| Hyrtioreticulins A-E, indole alkaloids inhibiting the ubiquitin-activating enzyme, from the marine sponge *Hyrtios reticulatus* | 2012 | Bioorganic & Medicinal Chemistry |
| Hyrtioseragamines A and B, new alkaloids from the sponge *Hyrtios* Species | 2011 | Organic Letters |
| Ianthellamide A, a selective kynurenine-3-hydroxylase inhibitor from the Australian marine sponge *Ianthella quadrangulata* | 2012 | Bioorganic & Medicinal Chemistry Letters |
| Ianthelliformisamines A-C, antibacterial bromotyrosine-derived metabolites from the marine sponge *Suberea ianthelliformis* | 2012 | Journal of Natural Products |
| Identification of a 3-alkylpyridinium compound from the red sea sponge *Amphimedon chloros* with in vitro inhibitory activity against the West Nile virus NS3 protease | 2018 | Molecules |
| Identification of new polyprenyl hydroquinone derivatives from tropical marine sponge *Ircinia* sp | 2012 | Heterocycles |
| Identification of novel acetylenic alcohols and a new dihydrothiopyranone from the tropical sponge *Reniochalina* sp | 2009 | Lipids |
| Imidazole alkaloids and their zinc complexes from the calcareous marine sponge *Leucetta chagosensis* | 2018 | Journal of Natural Products |
| Imidazole alkaloids from the South China Sea sponge *Pericharax heteroraphis* and their cytotoxic and antiviral activities | 2016 | Molecules |
| Immunomodulatory N-acyl dopamine glycosides from the Icelandic marine sponge *Myxilla incrustans* collected at a hydrothermal vent site | 2016 | Planta Medica |
| In Vitro pharmacological and toxicological effects of norterpene peroxides isolated from the Red Sea sponge *Diacarnus erythraeanus* on normal and cancer cells | 2013 | Journal of Natural Products |
| Incisterols, highly degraded marine sterols, are a new chemotype of PXR agonists | 2014 | Steroids |
| Indole derivatives produced by the metagenome genes of the escherichia coli-harboring marine sponge *Discodermia calyx* | 2017 | Molecules |
| Indoleamine 2,3-dioxygenase inhibitors isolated from the sponge *Xestospongia vansoesti*: structure elucidation, analogue synthesis, and biological activity | 2014 | Organic Letters |
| Indolo[3,2-a]carbazoles from a deep-water sponge of the genus *Asteropus* | 2013 | Journal of Natural Products |
| Inducers of hypoxic response: marine sesquiterpene quinones activate HIF-1 | 2013 | Journal of Natural Products |
| Ingenine E, a new cytotoxic β-carboline alkaloid from the Indonesian sponge *Acanthostrongylophora ingens* | 2017 | Journal of Asian Natural Products Research |
| Ingenine F: a new cytotoxic tetrahydro carboline alkaloid from the Indonesian marine sponge *Acanthostrongylophora ingens* | 2018 | Pharmacognosy Magazine |
| Ingenines A and B, two new alkaloids from the Indonesian sponge *Acanthostrongylophora ingens* | 2015 | Drug Res. (Stuttgart, Ger.) |
| Ingenines C and D, new cytotoxic pyrimidine-β-carboline alkaloids from the Indonesian sponge *Acanthostrongylophora ingenines* | 2016 | Phytochemistry Letters |
| Innovative approach to sustainable marine invertebrate chemistry and a scale-up technology for open marine ecosystems | 2018 | Marine Drugs |
| Investigation of the physical and bioactive properties of bromo- and iodo-containing sponge-derived compounds possessing an oxyphenylethanamine core | 2017 | Journal of Natural Products |
| Iodo-sesquiterpene hydroquinone and brominated indole alkaloids from the Thai sponge *Smenospongia* sp | 2012 | Tetrahedron |
| Iotrochamides A and B, antitrypanosomal compounds from the Australian marine sponge *Iotrochota* sp | 2012 | Bioorganic & Medicinal Chemistry Letters |
| Iotrochotamides I and II: new ceramides from the Indonesian sponge *Iotrochota purpurea* | 2009 | Natural Product Research |
| Irciformonins E - K, C22-Trinorsesterterpenoids from the sponge *Ircinia formosana* | 2009 | Helvetica Chimica acta |
| Ircinal E, a new manzamine derivative from the Indonesian marine sponge *Acanthostrongylophora ingens* | 2015 | Natural Product Communications |
| Ircinialactams: subunit-selective glycine receptor modulators from Australian sponges of the family Irciniidae | 2010 | Bioorganic & Medicinal Chemistry |
| Ishigadine A, a new canthin-6-one alkaloid from an Okinawan marine sponge *Hyrtios* sp. | 2018 | Tetrahedron Letters |
| Isoguanosine derivatives from the Northeastern Atlantic sponge *Clathria (Microciona) strepsitoxa* | 2017 | Tetrahedron Letters |
| Isohalitulin and Haliclorensins B and C, three marine alkaloids from *Haliclona tulearensis* | 2010 | Journal of Natural Products |
| Isolation and absolute configurations of diversiform C17, C21 and C25 terpenoids from the marine sponge *Cacospongia* sp | 2018 | Marine Drugs |
| Isolation and assessment of the in vitro anti-tumor activity of smenothiazole A and B, chlorinated thiazole-containing peptide/polyketides from the caribbean sponge, *Smenospongia aurea* | 2015 | Marine Drugs |
| Isolation and characterization of bioactive benzofuran sesquiterpene from marine sponge *Ircinia fusca* (Carter, 1880) | 2016 | Journal of Chemical, Biological and Physical Sciences |
| Isolation and characterization of diastereomers of discorhabdins H and K and assignment of absolute configuration to discorhabdins D, N, Q, S, T, and U | 2010 | Journal of Natural Products |
| Isolation and first total synthesis of PM050489 and PM060184, two new marine anticancer compounds | 2013 | Journal of the American Chemical Society |
| Isolation and identification of antitrypanosomal and antimycobacterial active steroids from the sponge *Haliclona simulans* | 2014 | Marine Drugs |
| Isolation and Structural elucidation of euryjanicins B-D, proline-containing cycloheptapeptides from the Caribbean marine sponge *Prosuberites laughlini* | 2009 | Journal of Natural Products |
| Isolation and structure determination of the biologically active sphingolipids from marine sponge *Haliclona* species | 2009 | Natural Product Research |
| Isolation and structure of a novel biindole pigment substituted with an ethyl group from a metagenomiclibrary derived from the marine sponge *Halichondria okadai* | 2012 | Chemistry letters |
| Isolation and structures of axistatins 1-3 from the Republic of Palau marine sponge *Agelas axifera Hentschel* | 2013 | Journal of Natural Products |
| Isolation and structures of pipecolidepsins A and B, cytotoxic cyclic depsipeptides from the Madagascan sponge *Homophymia lamellosa* | 2014 | Journal of Natural Products |
| Isolation and synthesis of N-acyladenine and adenosine alkaloids from a southern Australian marine sponge, *Phoriospongia* sp | 2014 | Tetrahedron Letters |
| Isolation and X-ray structure of deoxycholic acid from the sponge *Ircinia* sp | 2011 | Natural Product Communications |
| Isolation of acetylated bile acids from the sponge *Siphonochalina fortis* and DNA damage evaluation by the comet assay | 2013 | Steroids |
| Isolation of bastadin-6-O-sulfate and expedient purifications of bastadins-4, -5 and -6 from extracts of *Ianthella basta* | 2018 | Fitoterapia |
| Isolation of ciliatamide d from a marine sponge *Stelletta* sp. and a reinvestigation of the configuration of ciliatamide a | 2013 | Journal of Natural Products |
| Isolation of hydroxyoctaprenyl-1',4'-hydroquinone, a new octaprenylhydroquinone from the marine sponge *Sarcotragus spinosulus* and evaluation of its pharmacological activity on acetylcholine and glutamate release in the rat central nervous system | 2014 | Natural Product Communications |
| Isolation of smenopyrone, a bis-γ-pyrone polypropionate from the Caribbean sponge *Smenospongia aurea* | 2018 | Marine Drugs |
| Isolation of spirastrellolides A and B from a marine sponge *Epipolasis* sp. and their cytotoxic activities | 2012 | Journal of Natural Products |
| Isolation of steroidal glycosides from the Caribbean sponge *Pandaros acanthifolium* | 2012 | Journal of Natural Products |
| Isolation, characterization, and synthesis of the barrettides: disulfide-containing peptides from the marine sponge *Geodia barretti* | 2015 | Journal of Natural Products |
| Isolation, derivative synthesis, and structure-activity relationships of antiparasitic bromopyrrole alkaloids from the marine sponge *Tedania brasiliensis* | 2018 | Journal of Natural Products |
| Isolation, structural elucidation, and absolute stereochemistry of enigmazole a, a cytotoxic phosphomacrolide from the Papua New Guinea marine sponge *Cinachyrella enigmatica* | 2010 | Journal of the American Chemical Society |
| Isolation, structure determination and cytotoxicity studies of tryptophan alkaloids from an Australian marine sponge *Hyrtios* sp | 2014 | Bioorganic & Medicinal Chemistry Letters |
| Isolation, structures, and biological activities of triterpenoids from a *Penares* sp. marine sponge | 2013 | Journal of Natural Products |
| Isolation, synthesis, and biological activity of aphrocallistin, an adenine-substituted bromotyramine metabolite from the Hexactinellida sponge *Aphrocallistes beatrix* | 2009 | Journal of Natural Products |
| Isomalabaricane triterpenes with potent protein-tyrosine phosphatase 1B (PTP1B) inhibition from the Hainan sponge *Stelletta* sp | 2013 | Biochemical Systematics and Ecology |
| Isopetrosynol, a new protein tyrosine phosphatase lB Inhibitor, from the marine sponge *Halichondria cf. panicea* collected at iriomote island | 2016 | Chemical & Pharmaceutical Bulletin |
| Isorhizochalin: a minor unprecedented bipolar sphingolipid of stereodivergent biogenesis from the *Rhizochalina incrustata* | 2009 | Lipids |
| Isoswinholide B and swinholide K, potently cytotoxic dimeric macrolides from *Theonella swinhoei* | 2013 | Bioorganic & Medicinal Chemistry Letters |
| Jamaicensamide A, a peptide containing β-amino-α-keto and thiazole-homologated η-amino acid residues from the sponge *Plakina jamaicensis* | 2016 | Journal of Natural Products |
| Jasisoquinolines A and B, architecturally new isoquinolines, from a marine sponge *Jaspis* sp | 2011 | Organic Letters |
| Jaspamides M-P: new tryptophan modified jaspamide derivatives from the sponge *Jaspis splendans* | 2009 | Tetrahedron |
| Jaspiferin A and B: two new secondary metabolites from the South China Sea sponge *Jaspis stellifera* | 2012 | Records of Natural Products |
| Jaspiferin G, a new isomalabaricane-type triterpenoid from the sponge *Jaspis stellifera* | 2016 | Zeitschrift fur Naturforschung Section C-A Journal of Biosciences |
| Jaspiferins C-F, four new isomalabaricane-type triterpenoids from the South China Sea sponge *Jaspis stellifera* | 2014 | Journal of Asian Natural Products Research |
| Jaspiferins H-J, new isomalabaricane-type terpenoids from the South China Sea marine sponge *Jaspis stellifera* | 2018 | Chemistry of Natural Compounds |
| JBIR-44, a new bromotyrosine compound from a marine sponge *Psammaplysilla purpurea* | 2009 | The Journal of Antibiotics |
| Kabiramide L, a new antiplasmodial trisoxazole macrolide from the sponge *Pachastrissa nux* | 2013 | Natural Product Research |
| Kabiramides J and K, trisoxazole macrolides from the sponge *Pachastrissa nux* | 2011 | Journal of Natural Products |
| Lamellodysidines A and B, sesquiterpenes isolated from the marine sponge *Lamellodysidea herbacea* | 2017 | Journal of Natural Products |
| Lanesoic acid: a cytotoxic zwitterion from *Theonella* sp. | 2016 | Organic Letters |
| Lehualides E-K, cytotoxic metabolites from the Tongan marine sponge *Plakortis* sp | 2011 | Journal of Natural Products |
| Leiodermatolide, a potent antimitotic macrolide from the marine sponge *Leiodermatium* sp | 2011 | Angewandte Chemie-International Editio |
| Leucanone A and naamine J, glycerol ether lipid and imidazole alkaloid from the marine sponge *Leucandra* sp | 2017 | Journal of Asian Natural Products Research |
| Lingshuine, an unexpected passerini product from the Hainan sponge *Axinyssa variabilis* | 2009 | Helvetica Chimica acta |
| Lipodiscamides A-C, new cytotoxic lipopeptides from *Discodermia kiiensis* | 2014 | Organic Letters |
| Lipophilic 2,5-disubstituted pyrroles from the marine sponge *Mycale* sp. inhibit mitochondrial respiration and HIF-1 activation | 2009 | Journal of Natural Products |
| Lipophilic fractions from the marine sponge *Halichondria sitiens* decrease secretion of pro-inflammatory cytokines by dendritic cells and decrease their ability to induce a Th1 type response by allogeneic CD4+ T cells | 2017 | Pharmaceutical Biology |
| Liposomal circular dichroism. assignment of remote stereocenters in plakinic acids K and L from a *Plakortis-Xestospongia* sponge association | 2010 | Organic Letters |
| Lissodendoric acids A and B, manzamine-related alkaloids from the Far Eastern sponge *Lissodendoryx florida* | 2017 | Organic Letters |
| Lissodendrins A and B: 2-amino-imidazole alkaloids from the marine sponge *Lissodendoryx (Acanthodoryx) fibrosa* | 2016 | European Journal of Organic Chemistry |
| Luakuliides A-C, cytotoxic labdane diterpenes from a Tongan dictyoceratid sponge | 2015 | Tetrahedron Letters |
| Lysophospholipids from the Guangxi sponge *Spirastrella purpurea* | 2015 | Lipids |
| Macrocyclic bis-quinolizidine alkaloids from *Xestospongia muta* | 2018 | Natural Product Research |
| Manadodioxans A-E: polyketide endoperoxides from the marine sponge *Plakortis bergquistae* | 2015 | Journal of Natural Medicines |
| Manadoperoxides A-D from the Indonesian sponge *Plakortis cfr. simplex*. further insights on the structure-activity relationships of simple 1,2-dioxane antimalarials | 2010 | Journal of Natural Products |
| Manadoperoxides, a new class of potent antitrypanosomal agents of marine origin | 2012 | Organic & Biomolecular Chemistry |
| Manadosterols A and B, sulfonated sterol dimers inhibiting the Ubc13-Uev1A interaction, isolated from the marine sponge *Lissodendryx fibrosa* | 2012 | Journal of Natural Products |
| Manoalide-related sesterterpene from the marine sponge *Luffariella variabilis* | 2015 | Natural Product Communications |
| Manzamenone O, new trimeric fatty acid derivative from a marine sponge *Plakortis* sp | 2013 | Organic Letters |
| Manzamenones L-N, new dimeric fatty-acid derivatives from an Okinawan marine sponge *Plakortis* sp | 2013 | Bioorganic & Medicinal Chemistry Letters |
| Manzamine alkaloids from an *Acanthostrongylophora* sp. sponge | 2017 | Journal of Natural Products |
| Marine AChE inhibitors isolated from *Geodia barretti*: natural compounds and their synthetic analogs | 2016 | Organic & Biomolecular Chemistry |
| Megaspinoxide A: new norterpene cyclic peroxide from the sponge *Diacarnus megaspinorhabdosa* | 2014 | Natural Products Journal |
| Melonoside A: an ω-glycosylated fatty acid amide from the Far Eastern marine sponge *Melonanchora* *kobjakovae* | 2016 | Organic Letters |
| Melonoside B and melonosins a and b, lipids containing multifunctionalized ω‑hydroxy fatty acid amides from the Far Eastern marine sponge *Melonanchora kobjakovae* | 2018 | Journal of Natural Products |
| Merobatzelladines A and B, anti-infective tricyclic guanidines from a marine sponge *Monanchora* sp | 2009 | Organic Letters |
| Merosesquiterpene congeners from the Australian sponge Hyrtios digitatus as potential drug leads for atherosclerosis disease | 2017 | Marine Drugs |
| Meroterpenoids from a Tropical *Dysidea* sp. sponge | 2015 | Journal of Natural Products |
| Meroterpenoids with protein tyrosine phosphatase 1B inhibitory activity from a *Hyrtios* sp. marine sponge | 2017 | Journal of Natural Products |
| Metabolomic profiling reveals the N-acyl-taurine geodiataurine in extracts from the marine sponge *Geodia macandrewii (Bowerbank)* | 2016 | Journal of Natural Products |
| Metabolomics approach to chemical diversity of the Mediterranean marine sponge *Agelas oroides* | 2017 | Natural Product Research |
| Metachromins U-W: cytotoxic merosesquiterpenoids from an Australian specimen of the sponge *Thorecta reticulata* | 2011 | Journal of Natural Products |
| Minor brominated compounds from extract of the sponge *Aplysina* sp | 2013 | Chemistry of Natural Compounds |
| Mirabamides E-H, HIV-inhibitory depsipeptides from the sponge *Stelletta clavosa* | 2011 | Journal of Natural Products |
| Mirabilins revisited: polyketide alkaloids from a southern Australian marine sponge, *Clathria* sp | 2010 | Organic & Biomolecular Chemistry |
| Mirabolides A and B; new cytotoxic glycerides from the red sea sponge *Theonella mirabilis* | 2016 | Marine Drugs |
| Miuramides A and B, trisoxazole macrolides from a *Mycale* sp. marine sponge that induce a protrusion phenotype in cultured mammalian cells | 2018 | Journal of Natural Products |
| Miyakosynes A-F, cytotoxic methyl branched acetylenes from a marine sponge *Petrosia* sp | 2011 | Tetrahedron |
| Mollenyne A, a long-chain chlorodibromohydrin amide from the sponge *Spirastrella mollis* | 2011 | Organic Letters |
| Mollenynes B-E from the marine sponge *Spirastrella mollis*. Band-selective heteronuclear single quantum coherence for discrimination of bromo-chloro regioisomerism in natural products | 2015 | Journal of the American Chemical Society |
| Monamphilectine A, a potent antimalarial β-lactam from marine sponge *Hymeniacidon* sp: isolation, structure, semisynthesis, and bioactivity | 2010 | Organic Letters |
| Monanchocidin: a new apoptosis-inducing polycyclic guanidine alkaloid from the marine sponge *Monanchora pulchra* | 2010 | Organic Letters |
| Monanchocidins B-E: polycyclic guanidine alkaloids with potent antileukemic activities from the sponge *Monanchora pulchra* | 2011 | Journal of Natural Products |
| Monanchomycalin C, a new pentacyclic guanidine alkaloid from the Far-Eastern marine sponge *Monanchora pulchra* | 2013 | Natural Product Communications |
| Monanchomycalins A and B, unusual guanidine alkaloids from the sponge *Monanchora pulchra* | 2012 | Tetrahedron Letters |
| Monanchoramides A-D, ceramides from the marine sponge *Monanchora clathrata* with cytotoxic activity | 2018 | Phytochemistry Letters |
| Monanchosterols A and B, bioactive bicyclo[4.3.1]steroids from a Korean sponge *Monanchora* sp | 2015 | Journal of Natural Products |
| Monanchoxymycalin C with anticancer properties, new analogue of crambescidin 800 from the marine sponge *Monanchora pulchra* | 2017 | Natural Product Research |
| Monanchoxymycalins A and B, new hybrid pentacyclic guanidine alkaloids from the Far-Eastern marine sponge *Monanchora pulchra* | 2016 | Natural Product Communications |
| Monoindole alkaloids from a marine sponge *Mycale fibrexilis* | 2012 | Biochemical Systematics and Ecology |
| Motualevic acids A-F, antimicrobial acids from the sponge *Siliquariaspongia* sp | 2009 | Organic Letters |
| Mutremdamide A and koshikamides C-H, peptide inhibitors of HIV-1 entry from different *Theonella* species | 2010 | Journal of Organic Chemistry |
| Muurolane-type sesquiterpenes from marine sponge *Dysidea cinerea* | 2014 | Magnetic Resonance in Chemistry |
| Nagelamide I and 2,2'-didebromonagelamide B, new dimeric bromopyrrole-imidazole alkaloids from a marine sponge *Agelas* sp | 2014 | Chemical & Pharmaceutical Bulletin |
| Nagelamides Q and R, novel dimeric bromopyrrole alkaloids from sponges *Agelas* sp | 2009 | Organic Letters |
| Nagelamides U-W, bromopyrrole alkaloids from a marine sponge *Agelas* sp | 2013 | Tetrahedron Letters |
| Nagelamides X-Z, dimeric bromopyrrole alkaloids from a marine sponge *Agelas* sp | 2013 | Organic Letters |
| Nakijinamines C-E, new heteroaromatic alkaloids from the sponge *Suberites* Species | 2011 | Organic Letters |
| Nakijiquinone S and nakijinol C, new meroterpenoids from a marine sponge of the family Spongiidae | 2014 | Chemical & Pharmaceutical Bulletin |
| Nakijiquinones E and F, new dimeric sesquiterpenoid quinones from marine sponge | 2009 | Bioorganic & Medicinal Chemistry |
| Nakijiquinones J-R, sesquiterpenoid quinones with an amine residue from Okinawan marine sponges | 2010 | Journal of Natural Products |
| Naphtoquinones and sesquiterpene cyclopentenones from the sponge *Smenospongia cerebriformis* with their cytotoxic activity | 2017 | Chemical & Pharmaceutical Bulletin |
| Natural and semisynthetic analogues of manadoperoxide B reveal new structural requirements for trypanocidal activity | 2013 | Marine Drugs |
| Natural bioactive compounds from *Hemimycale aff arabica*: antimicrobial, antiglycation, cytotoxicity, and molecular docking studies | 2018 | Medicinal Chemistry Research |
| Nazumazoles A-C, cyclic pentapeptides dimerized through a disulfide bond from the marine sponge *Theonella swinhoei* | 2015 | Organic Letters |
| Nazumazoles D-F, cyclic pentapeptides that inhibit chymotrypsin, from the marine sponge *Theonella swinhoei* | 2016 | Journal of Natural Products |
| N-containing metabolites from the marine sponge *Agelas clathrodes* | 2013 | Natural Product Communications |
| N-Demethylaaptanone, A new congener of aaptamine alkaloids from the Vietnamese marine sponge *Aaptos aaptos* | 2016 | Natural Product Communications |
| N-Didehydrotyrosine identified from the Northeastern Atlantic marine sponge *Hymeniacidon perlevis* after chemical screening | 2017 | Natural Product Communications |
| Neamphamide B, new cyclic depsipeptide, as an anti-dormant mycobacterial substance from a Japanese marine sponge of *Neamphius* sp | 2012 | Bioorganic & Medicinal Chemistry Letters |
| Neopetrocyclamines A and B, polycyclic diamine alkaloids from the sponge *Neopetrosia cf exigua* | 2015 | Journal of Natural Products |
| Neopetrosiamine A, biologically active bis-piperidine alkaloid from the Caribbeansea sponge *Neopetrosia* *proxima* | 2010 | Bioorganic & Medicinal Chemistry Letters |
| Neopetrosiquinones A and B, sesquiterpene benzoquinones isolated from the deep-water sponge *Neopetrosia cf. proxima* | 2011 | Bioorganic & Medicinal Chemistry |
| Nepheliosyne B, a new polyacetylenic acid from the new caledonian marine sponge *Niphates* sp | 2013 | Marine Drugs |
| Netamines H-N, tricyclic alkaloids from the marine sponge *Biemna laboutei* and their antimalarial activity | 2014 | Journal of Natural Products |
| Netamines O-S, five new tricyclic guanidine alkaloids from the Madagascar sponge *Biemna laboutei*, and their antimalarial activities | 2015 | Chemistry & Biodiversity |
| New 17-methyl-13-octadecenoic and 3,16-docosadienoic acids from the sponge *Polymastia penicillus* | 2009 | Lipids |
| New 2-methoxy acetylenic acids and pyrazole alkaloids from the marine sponge *Cinachyrella* sp | 2017 | Marine Drugs |
| New 2-methyl-13-icosenoic acid from the temperate calcisponge *Leuconia johnstoni* | 2012 | Lipids |
| New 4-methylidene sterols from the marine sponge *Theonella swinhoei* | 2018 | Fitoterapia |
| New 9α-hydroxy-5α,6α-epoxysterols from the Vietnamese marine sponge *Ircinia echinata* | 2018 | Marine Drugs |
| New antibacterial sesquiterpene aminoquinones from a Vietnamese marine sponge of *Spongia* sp | 2016 | Phytochemistry Letters |
| New anti-inflammatory sterols from the Red Sea sponges *Scalarispongia aqabaensis* and *Callyspongia siphonella* | 2010 | Natural Product Communications |
| New antimalarial norterpene cyclic peroxides from Xisha Islands sponge *Diacarnus megaspinorhabdosa* | 2016 | Bioorganic & Medicinal Chemistry Letters |
| New antimalarial polyketide endoperoxides from the marine sponge *Plakinastrella mamillaris* collected at Fiji Islands | 2013 | Tetrahedron |
| New antimicrobial bromotyrosine analogues from the sponge *Pseudoceratina purpurea* and its predator Tylodina corticalis | 2015 | Marine Drugs |
| New antiplasmodial bromotyrosine derivatives from *Suberea ianthelliformis* Lendenfeld, 1888 | 2012 | Chemistry & Biodiversity |
| New bioactive alkaloids from the marine sponge *Stylissa* sp | 2012 | Tetrahedron |
| New bioactive halenaquinone derivatives from South Pacific marine sponges of the genus *Xestospongia* | 2010 | Bioorganic & Medicinal Chemistry |
| New bromoindole alkaloid isolated from the marine sponge *Hyrtios erectus* | 2018 | Heterocycles |
| New bromoindole alkaloid isolated from the marine sponge Hyrtios erectus | 2018 | Heterocycles |
| New bromopyrrole alkaloids from the marine sponge *Agelas* sp | 2017 | Tetrahedron |
| New butenolide and pentenolide from *Dysidea cinerea* | 2013 | Natural Product Communications |
| New candidaspongiolides, tedanolide analogues that selectively inhibit melanoma cell growth | 2011 | Organic Letters |
| New cerebrosides from a marine sponge *Haliclona* (*Reniera*) sp | 2009 | Chemical & Pharmaceutical Bulletin |
| New compounds from the Red Sea marine sponge *Echinoclathria gibbosa* | 2014 | Phytochemistry Letters |
| New constituents from the Korean sponge *Plakortis simplex* | 2013 | Marine Drugs |
| New crambescidin-type alkaloids from the Indonesian marine sponge *Clathria bulbotoxa* | 2018 | Marine Drugs |
| New cyclic cystine bridged peptides from the sponge *Suberites waedoensis* | 2014 | Marine Drugs |
| New cyclitol derivative from a sponge *Sarcotragus* species | 2011 | Natural Product Research |
| New cytotoxic 24-homoscalarane sesterterpenoids from the sponge *Ircinia felix* | 2015 | International Journal of Molecular Sciences |
| New cytotoxic callipeltins from the Solomon Island marine sponge *Asteropus* sp | 2016 | Tetrahedron |
| New cytotoxic cyclic peroxide acids from *Plakortis* sp. marine sponge | 2015 | ARKIVOC (Gainesville, FL, U. S.) |
| New cytotoxic spongian diterpenes from the sponge *Dysidea cf. arenaria* | 2009 | Tetrahedron |
| New cytotoxic spongian-class rearranged diterpenes from a marine sponge | 2012 | Chemistry of Natural Compounds |
| New dictyodendrins as BACE inhibitors from a southern Australian marine sponge, *Ianthella* sp | 2012 | RSC Advances |
| New diterpene alkaloids from the marine sponge *Agelas mauritiana* | 2017 | RSC Advances |
| New diterpenoids from the marine sponge *Dactylospongia elegans* | 2017 | Tetrahedron |
| New epoxy-substituted nitrogenous bisabolene-type sesquiterpenes from a Hainan sponge *Axinyssa* sp | 2010 | Helvetica Chimica acta |
| New fatty acids from Colombian Caribbean Sea sponges | 2010 | Biochemical Systematics and Ecology |
| New fatty acids from the Red Sea sponge *Mycale euplectelloides* | 2014 | Natural Product Research |
| New haliclamines E and F from the Arctic sponge *Haliclona viscosa* | 2009 | Organic & Biomolecular Chemistry |
| New highly oxidized formamidobisabolene-derived sesquiterpenes from a Hainan sponge *Axinyssa variabilis* | 2016 | Helvetica Chimica acta |
| New hippolide derivatives with protein tyrosine phosphatase 1B inhibitory activity from the marine sponge *Hippospongia lachne* | 2014 | Marine Drugs |
| New indole alkaloids from the sponge *Plakortis* sp | 2015 | Chemistry of Natural Compounds |
| New inhibitors of RANKL-induced osteoclastogenesis from the marine sponge *Siphonochalina siphonella* | 2018 | Fitoterapia |
| New isomalabaricane analogues from the sponge *Rhabdastrella providentiae* and their cytotoxic activities | 2018 | Phytochemistry Letters |
| New isoquinolinequinone alkaloids from the South China Sea nudibranch *Jorunna funebris* and its possible sponge-prey *Xestospongia* sp | 2014 | Fitoterapia |
| New merosesquiterpenes from a Vietnamese marine sponge of *Spongia* sp. and their biological activities | 2017 | Bioorganic & Medicinal Chemistry Letters |
| New meroterpenoids from the marine sponge *Aka coralliphaga* | 2012 | Natural Product Communications |
| New metabolites from the South China sea sponge *Diacarnus megaspinorhabdosa* | 2015 | Chemical & Pharmaceutical Bulletin |
| New natural products in the discorhabdin A- and B-series from New Zealand-sourced *Latrunculia* spp. sponges | 2009 | Tetrahedron |
| New nitrogenous bisabolene-type sesquiterpenes from a Formosan sponge *Axinyssa* sp | 2014 | Chemical & Pharmaceutical Bulletin |
| New nitrogenous compounds from a Red Sea sponge from the Gulf of Aqaba | 2015 | Zeitschrift fur Naturforschung Section C-A Journal of Biosciences |
| New polyhydroxy sterols from the marine sponge *Callyspongia fibrosa* (Ridley & Dendly) | 2010 | Tetrahedron Letters |
| New polyunsaturated amino ketones from a Guangxi sponge *Haliclona* sp | 2010 | Helvetica Chimica acta |
| New pregnane steroids from Formosan red alga Ceratodictyon spongiosum and symbiotic sponge *Sigmadocia symbiotica* | 2011 | Bulletin of the Chemical Society of Japan |
| New scalarane sesterterpenoids from the formosan sponge *Ircinia felix* | 2015 | Marine Drugs |
| New sesquiterpene hydroquinones from the Caribbean sponge *Aka coralliphagum* | 2014 | Beilstein Journal of Organic Chemistry |
| New steroids with a rearranged skeleton as (h)P300 inhibitors from the sponge *Theonella swinhoei* | 2014 | Organic Letters |
| New sterol derivatives from the marine sponge *Xestospongia* sp | 2016 | Helvetica Chimica acta |
| New structures and bioactivity properties of jasplakinolide (jaspamide) analogues from marine sponges | 2010 | Journal of Medicinal Chemistry |
| New sulfur-containing polyarsenicals from the New Caledonian sponge *Echinochalina bargibanti* | 2018 | Marine Drugs |
| New tridecapeptides of the theonellapeptolide family from the Indonesian sponge *Theonella swinhoei* | 2013 | Beilstein Journal of Organic Chemistry |
| New triterpene oligoglycosides from the Caribbean sponge *Erylus formosus* | 2011 | Carbohydrate Research |
| New β-carboline alkaloid from marine sponge *Hyrtios reticulatus* | 2012 | Biosciences Biotechnology Research Asia |
| Niphatenones, glycerol ethers from the sponge *Niphates digitalis* block androgen receptor transcriptional activity in prostate cancer cells: structure elucidation, synthesis, and biological activity | 2012 | Journal of Medicinal Chemistry |
| Niphateolide A: isolation from the marine sponge *Niphates olemda* and determination of its absolute configuration by an ECD analysis | 2015 | Tetrahedron |
| Nitrogenous sesquiterpenes from the Thai marine sponge *Halichondria* sp | 2011 | Tetrahedron |
| Njaoamine I, a cytotoxic polycyclic alkaloid from the Haplosclerida sponge *Haliclona* (*Reniera*) sp | 2018 | Tetrahedron Letters |
| Njaoaminiums A, B, and C: cyclic 3-alkylpyridinium salts from the marine sponge *Reniera* sp | 2009 | Molecules |
| N-Methylniphatync A, a new 3-alkylpyridine alkaloid as an inhibitor of the cancer cells adapted to nutrient starvation, from an indonesian marine sponge of *Xestospongia* sp | 2016 | Chemical & Pharmaceutical Bulletin |
| NMR fingerprints of the drug-like natural-product space identify iotrochotazine a: a chemical probe to study Parkinson's disease | 2014 | Angewandte Chemie-International Editio |
| NMR strategy for unraveling structures of bioactive sponge-derived oxy-polyhalogenated diphenyl ethers | 2009 | Journal of Natural Products |
| Norisoprenoids from the marine sponge *Spheciospongia* sp | 2009 | Journal of Asian Natural Products Research |
| Normonanchocidins A, B and D, new pentacyclic guanidine alkaloids from the Far-Eastern marine sponge *Monanchora pulchra* | 2015 | Natural Product Communications |
| Normonanchocidins G and H, new pentacyclic guanidine alkaloids from the Far-Eastern marine sponge *Monanchora pulchra* | 2017 | Natural Product Communications |
| Norselic acids A-E, highly oxidized anti-infective steroids that deter mesograzer predation, from the Antarctic sponge *Crella* sp | 2009 | Journal of Natural Products |
| Norterpenoids and related peroxides from the Formosan marine sponge *Negombata corticata* | 2010 | Journal of Natural Products |
| Nortriterpene glycosides of the Sarasinoside class from the sponge *Lipastrotethya* sp | 2012 | Journal of Natural Products |
| Novel adociaquinone derivatives from the Indonesian sponge *Xestospongia* sp | 2015 | Marine Drugs |
| Novel cytotoxic polyoxygenated steroids from an okinawan sponge *Dysidea* sp | 2012 | Bioscience, Biotechnology, and Biochemistry |
| Novel N-methylated 8-oxoisoguanines from Pacific sponges with diverse neuroactivities | 2010 | Journal of Medicinal Chemistry |
| Novel very long-chain α-methoxylated Δ5,9 fatty acids from the sponge *Asteropus niger* are effective inhibitors of topoisomerases IB | 2016 | Lipids |
| Occurrence of a taurine derivative in an antarctic glass sponge | 2014 | Natural Product Communications |
| On the mechanism of action of dragmacidins I and J, two new representatives of a new class of protein phosphatase 1 and 2A inhibitors | 2018 | ACS Omega |
| Oscarellin, an anthranilic acid derivative from a Philippine sponge, *Oscarella stillans*, as an inhibitor of inflammatory cytokines in macrophages | 2017 | Journal of Natural Products |
| Oxidative processes in the Australian marine sponge *Plakinastrella clathrata*: isolation of plakortolides with oxidatively modified side chains | 2012 | Journal of Natural Products |
| Oxygenated 4-methylidene sterols from the South China Sea sponge *Theonella swinhoei* | 2010 | Helvetica Chimica acta |
| Oxygenated polyketides from *Plakinastrella mamillaris* as a new chemotype of PXR agonists | 2013 | Marine Drugs |
| Oxygenated terpenes from Indo-Pacific nudibranchs: scalarane sesterterpenes from *Glossodoris hikuerensis* and 12-acetoxy dendrillolide A from *Goniobranchus albonares* | 2015 | Natural Product Communications |
| Oxygenated terpenoids from the Australian sponges *Coscinoderma matthewsi* and *Dysidea* sp., and the Nudibranch *Chromodoris albopunctata* | 2012 | Australian Journal of Chemistry |
| Oxysterols from a marine sponge *Inflatella* sp. and their action in 6-hydroxydopamine-induced cell model of Parkinson's disease | 2018 | Marine Drugs |
| Paltolides A-C, anabaenopeptin-type peptides from the Palau sponge *Theonella swinhoei* | 2010 | Journal of Natural Products |
| Papuamides E and F, cytotoxic depsipeptides from the marine sponge *Melophlus* sp | 2011 | Tetrahedron |
| Pellynols M-O, cytotoxic polyacetylenic alcohols from a *Niphates* sp. marine sponge | 2018 | Tetrahedron |
| Peloruside B, a potent antitumor macrolide from the New Zealand marine sponge *Mycale hentscheli*: isolation, structure, total synthesis, and bioactivity | 2010 | Journal of Organic Chemistry |
| Peloruside E (22-Norpeloruside A), a pelorusane macrolide from the New Zealand marine sponge *Mycale hentscheli*, retains microtubule-stabilizing properties | 2018 | Journal of Natural Products |
| Pembamide, a N-methylated linear peptide from a sponge *Cribrochalina* sp | 2016 | Tetrahedron Letters |
| Penasins A-E, long-chain cytotoxic sphingoid bases, from a marine sponge *Penares* sp | 2010 | Journal of Natural Products |
| Pentacyclic ingamine alkaloids, a new antiplasmodial pharmacophore from the marine sponge *Petrosid* Ng5 Sp5 | 2012 | Planta Medica |
| Peroxide natural products from *Plakortis zyggompha* and the sponge association *Plakortis halichondrioides*-*Xestospongia deweerdtae*: antifungal activity against Cryptococcus gattii | 2016 | Journal of Natural Products |
| Perthamides C and D, two new potent anti-inflammatory cyclopeptides from a Solomon Lithistid sponge *Theonella swinhoei* | 2009 | Tetrahedron |
| Perthamides C-F, potent human antipsoriatic cyclopeptides | 2011 | Tetrahedron |
| Petroacetylene, a new polyacetylene from the marine sponge *Petrosiasolida* that inhibits blastulation of starfish embryos | 2013 | Natural Product Research |
| Petroquinones: trimeric and dimeric xestoquinone derivatives isolated from the marine sponge *Petrosia alfiani* | 2016 | Tetrahedron |
| Petrosiols A-E, neurotrophic diyne tetraols isolated from the Okinawan sponge *Petrosia strongylata* | 2013 | Tetrahedron |
| Phorbaketals A, B, and C, sesterterpenoids with a spiroketal of hydrobenzopyran moiety isolated from the marine sponge *Phorbas* sp | 2009 | Organic Letters |
| Phorbaketals L-N, cytotoxic sesterterpenoids isolated from the marine sponge of the genus *Phorbas* | 2014 | Bioorganic & Medicinal Chemistry Letters |
| Phorbasones A and B, sesterterpenoids isolated from the marine sponge *Phorbas* sp. and induction of osteoblast differentiation | 2011 | Organic Letters |
| Phormidolides B and C, cytotoxic agents from the sea: enantioselective synthesis of the macrocyclic core | 2015 | Chemistry-A European Journal |
| Phorone A and isophorbasone a, sesterterpenoids isolated from the marine sponge *Phorbas* sp | 2012 | Organic Letters |
| Phosphocalyculin C as a pyrophosphate protoxin of calyculin C in the marine sponge *Discodermia calyx* | 2014 | Bioorganic & Medicinal Chemistry Letters |
| Phosphoiodyns A and B, unique phosphorus-containing iodinated polyacetylenes from a Korean sponge *Placospongia* sp | 2013 | Organic Letters |
| Pinnarine, another member of the Halichlorine family. isolation and preparation from pinnaic acid | 2011 | Journal of Natural Products |
| Pipestelides A-C: cyclodepsipeptides from the Pacific marine sponge *Pipestela candelabra* | 2012 | Journal of Natural Products |
| Placotylene A, an inhibitor of the receptor activator of nuclear factor-κB ligand-induced osteoclast differentiation, from a Korean sponge *Placospongia* sp | 2014 | Marine Drugs |
| Plakilactones from the marine sponge *Plakinastrella mamillaris*. discovery of a new class of marine ligands of peroxisome proliferator-activated receptor γ | 2012 | Journal of Medicinal Chemistry |
| Plakilactones G and H from a marine sponge. Stereochemical determination of highly flexible systems by quantitative NMR-derived interproton distances combined with quantum mechanical calculations of 13C chemical shifts | 2013 | Beilstein Journal of Organic Chemistry |
| Plakinamine L: a new steroidal alkaloid from the marine sponge *Corticium* sp | 2010 | Natural Product Communications |
| Plakinamine M, a steroidal alkaloid from the marine sponge *Corticium* sp | 2013 | Journal of Natural Products |
| Plakofuranolactone as a quorum quenching agent from the Indonesian sponge *Plakortis cf. lita* | 2017 | Marine Drugs |
| Plakoridine C, a novel piperidine alkaloid from an Okinawan marine sponge *Plakortis* sp | 2009 | Tetrahedron Letters |
| Plakortinic acids A and B: cytotoxic cycloperoxides with a bicyclo[4.2.0]octene unit from sponges of the genera *Plakortis* and *Xestospongia* | 2017 | Organic Letters |
| Plastisidines A-C, N-methylpyridinium alkaloids from an Okinawan marine sponge of *Plakortis* species | 2010 | Heterocycles |
| Poecillastrin E, F, and G, cytotoxic chondropsin-type macrolides from a marine sponge *Poecillastra* sp | 2018 | Tetrahedron |
| Poecillastrin H, a chondropsin-type macrolide with a conjugated pentaene moiety, from a *Characella* sp. marine sponge | 2018 | Journal of Natural Products |
| Poecillastrosides, steroidal saponins from the Mediterranean Deep-Sea sponge *Poecillastra compressa* (Bowerbank, 1866) | 2017 | Marine Drugs |
| Polyaxibetaine, an amino acid derivative from the marine sponge *Axinella polypoides* | 2010 | Journal of Natural Products |
| Polybrominated diphenyl ethers with potent and broad spectrum antimicrobial activity from the marine sponge *Dysidea* | 2015 | Bioorganic & Medicinal Chemistry Letters |
| Polybrominated diphenyl ethers: structure determination and trends in antibacterial activity | 2016 | Journal of Natural Products |
| Polyketide-peroxides from a species of *Jamaican Plakortis* (Porifera: Demospongiae) | 2010 | Australian Journal of Chemistry |
| Polyoxygenated steroids from the sponge *Clathria gombawuiensis* | 2017 | Journal of Natural Products |
| Popolohuanones G-I, dimeric sesquiterpene quinones with IL-6 inhibitory activity from the marine sponge *Dactylospongia elegans* | 2018 | Chemistry & Biodiversity |
| Potent cytotoxic peptides from the Australian marine sponge *Pipestela candelabra* | 2014 | Marine Drugs |
| PPAR modulating polyketides from a Chinese Plakortis simplex and clues on the origin of their chemodiversity | 2016 | Journal of Organic Chemistry |
| Pregnane-10,2-carbolactones from a Hawaiian marine sponge in the genus *Myrmekioderma* | 2016 | Journal of Natural Products |
| Proline-containing cyclopeptides from the marine sponge *Phakellia fusca* | 2010 | Journal of Natural Products |
| Proline-containing dipeptides from a marine sponge of a *Callyspongia* Species | 2009 | Helvetica Chimica acta |
| Protein tyrosine phosphatase 1B inhibitory polybromobiphenyl ethers and monocyclofarnesol-type sesquiterpenes from the Indonesian marine sponge *Lamellodysidea cf. herbacea* | 2018 | Phytochemistry Letters |
| Protoaculeine B, a putative N-terminal residue for the novel peptide toxin aculeines | 2014 | Organic Letters |
| Psammaplin metabolites new and old: an nmr study involving chiral sulfur chemistry | 2010 | Australian Journal of Chemistry |
| Psammaplysin derivatives from the Balinese marine sponge *Aplysinella strongylata* | 2012 | Journal of Natural Products |
| Psammaplysin H, a new antimalarial bromotyrosine alkaloid from a marine sponge of the genus *Pseudoceratina* | 2011 | Bioorganic & Medicinal Chemistry Letters |
| Pseudoceramines A-D, new antibacterial bromotyrosine alkaloids from the marine sponge *Pseudoceratina* sp | 2011 | Organic & Biomolecular Chemistry |
| Pseudoceratinazole A: a novel bromotyrosine alkaloid from the Australian sponge *Pseudoceratina* sp | 2010 | Tetrahedron Letters |
| Pulchranin A, isolated from the Far-Eastern marine sponge, *Monanchora pulchra*: the first marine non-peptide inhibitor of TRPV-1 channels | 2013 | Tetrahedron Letters |
| Pulchranins B and C, new acyclic guanidine alkaloids from the far-eastern marine sponge *Monanchora pulchra* | 2013 | Natural Product Communications |
| Purpuroines A-J, halogenated alkaloids from the sponge *Iotrochota purpurea* with antibiotic activity and regulation of tyrosine kinases | 2012 | Bioorganic & Medicinal Chemistry |
| Pursuing sesterterpene lactams in Australian Irciniidae sponges | 2018 | Fitoterapia |
| Puupehanol, a sesquiterpene-dihydroquinone derivative from the marine sponge *Hyrtios* sp | 2009 | Bioorganic & Medicinal Chemistry Letters |
| Puupehenol, a potent antioxidant antimicrobial meroterpenoid from a Hawaiian deep-water *Dactylospongia* sp. sponge | 2015 | Journal of Natural Products |
| Pyridine nucleosides neopetrosides a and b from a marine *Neopetrosia* sp. sponge. synthesis of neopetroside A and its β-riboside analogue | 2015 | Journal of Natural Products |
| Pyrinodemins E and F, new 3-alkylpyridine alkaloids from sponge *Amphimedon* sp | 2011 | Bioorganic & Medicinal Chemistry Letters |
| Pyrinodemins G-I, new bis-3-alkylpyridine alkaloids from a marine sponge *Amphimedon* sp | 2013 | Tetrahedron |
| Pyrodysinoic acid derivatives from the marine sponge *Dysidea robusta* | 2009 | Journal of Natural Products |
| Pyroglutamyl dipeptides and tetrahydro-β-carboline alkaloids from a marine sponge *Asteropus* sp | 2010 | Biochemical Systematics and Ecology |
| Pyrrole derivatives and diterpene alkaloids from the South China Sea sponge *Agelas nakamurai* | 2017 | Chemistry & Biodiversity |
| Quantitative determination of phakellistatin 13, a new cyclic heptapeptide, in rat plasma by liquid chromatography/tandem mass spectrometry: application to a pharmacokinetic study | 2009 | Analytical and Bioanalytical Chemistry |
| Quantitative nmr-derived interproton distances combined with quantum mechanical calculations of ^13^C chemical shifts in the stereochemical determination of conicasterol F, a nuclear receptor ligand from *Theonella swinhoei* | 2012 | Journal of Organic Chemistry |
| Quinolizidine alkaloids. Petrosin and xestospongins from the sponge *Oceanapia* sp | 2011 | Journal of Chemical Sciences |
| Racemic trinorsesquiterpenoids from the Beihai sponge *Spongia officinalis*: structure and biomimetic total synthesis | 2018 | Organic Chemistry Frontiers |
| Rearranged terpenoids from the marine sponge *Darwinella cf. oxeata* and its predator, the nudibranch *Felimida grahami* | 2017 | Journal of Natural Products |
| Relative and absolute stereochemistry of diacarperoxides: antimalarial norditerpene endoperoxides from marine sponge *Diacarnus megaspinorhabdosa* | 2014 | Marine Drugs |
| Reniochalistatins A-E, cyclic peptides from the marine sponge *Reniochalina stalagmitis* | 2014 | Journal of Natural Products |
| Reticulatins A and B and hyrtioreticulin F from the marine sponge *Hyrtios reticulatus* | 2013 | Tetrahedron |
| Reversal of fluconazole resistance by sulfated sterols from the marine sponge *Topsentia* sp | 2009 | Journal of Natural Products |
| Revised structure of cyclolithistide a, a cyclic depsipeptide from the marine sponge *Discodermia japonica* | 2014 | Journal of Natural Products |
| Rhodocomatulin-type anthraquinones from the Australian marine invertebrates *Clathria hirsuta* and *Comatula rotalaria* | 2016 | Journal of Natural Products |
| Rolloamides A and B, cytotoxic cyclic heptapeptides isolated from the Caribbean marine sponge *Eurypon laughlini* | 2009 | Journal of Natural Products |
| Salaramides A and B; two α-oxoamides isolated from the marine sponge *Hippospongia* sp. (Porifera, Dictyoceratida) | 2010 | Natural Product Communications |
| Salarins D-J, seven new nitrogenous macrolides from the madagascar sponge *Fascaplysinopsis* sp | 2010 | Tetrahedron |
| Scalalactams A-D, scalarane sesterterpenes with a g-lactam moiety from a Korean *Spongia* sp. marine sponge | 2018 | Molecules |
| Scalarane sesterterpenes from the Chinese sponge *Phyllospongia foliascens* | 2009 | Helvetica Chimica acta |
| Scalarane sesterterpenes from the Egyptian Red Sea sponge *Phyllospongia lamellosa* | 2015 | Tetrahedron |
| Scalarane sesterterpenes from the Paracel Islands marine sponge *Hyrtios* sp | 2014 | Natural Product Communications |
| Scalarane sesterterpenes from the sponge *Hyatella* sp | 2011 | Journal of Natural Products |
| Scalarane sesterterpenes from Thorectidae sponges as inhibitors of TDP-43 nuclear factor | 2014 | Organic & Biomolecular Chemistry |
| Scalarane-based sesterterpenoid RCE-protease inhibitors isolated from the Indonesian marine sponge *Carteriospongia foliascens* | 2009 | Journal of Natural Products |
| Sequestered fulvinol-related polyacetylenes in Peltodoris atromaculata | 2014 | Journal of Natural Products |
| Sesquiterpene benzoxazoles and sesquiterpene quinones from the marine sponge *Dactylospongia elegans* | 2011 | Journal of Natural Products |
| Sesquiterpene derivatives from marine sponge Smenospongia cerebriformis and their anti-inflammatory activity | 2017 | Bioorganic & Medicinal Chemistry Letters |
| Sesquiterpene hydroquinones with protein tyrosine phosphatase 1B inhibitory activities from a *Dysidea* sp. marine sponge collected in Okinawa | 2016 | Journal of Natural Products |
| Sesquiterpene isocyanides, isothiocyanates, thiocyanates, and formamides from the Thai sponge *Halichondria* sp | 2016 | Tetrahedron |
| Sesquiterpene quinones and diterpenes from *Smenospongia cerebriformis* and their cytotoxic activity | 2017 | Natural Product Communications |
| Sesquiterpene quinones from a Viet Nam sea sponge *Spongia* Sp | 2011 | Chemistry of Natural Compounds |
| Sesquiterpene quinones/hydroquinones from the marine sponge *Spongia pertusa* Esper | 2017 | Journal of Natural Products |
| Sesquiterpenes from the Vietnamese marine sponge *Dysidea fragilis* | 2015 | Natural Product Communications |
| Sesquiterpenoid aminoquinones from the marine sponge *Dysidea* sp | 2010 | Journal of Natural Products |
| Sesquiterpenoids with PPARδ agonistic effect from a Korean marine sponge *Ircinia* sp | 2014 | Tetrahedron Letters |
| Sesterterpene glycinyl-lactams: a new class of glycine receptor modulator from Australian marine sponges of the genus *Psammocinia* | 2013 | Organic & Biomolecular Chemistry |
| Sesterterpenes and a new sterol from the marine sponge *Phyllospongia foliascens* | 2010 | Molecules |
| Sesterterpenes and phenolic alkenes from the Thai sponge *Hyrtios erectus* | 2018 | Tetrahedron |
| Sesterterpenes from the sponge *Dysidea* sp | 2010 | Zeitschrift fur Naturforschung Section B-A Journal of Chemical Sciences |
| Sesterterpenes from the tropical sponge *Coscinoderma* sp | 2011 | Journal of Natural Products |
| Sesterterpenoid and steroid metabolites from a deep-water alaska sponge inhibit Wnt/β-catenin signaling in colon cancer cells | 2018 | Marine Drugs |
| Sesterterpenoids isolated from a northeastern Pacific *Phorbas* sp | 2013 | Journal of Organic Chemistry |
| Sesterterpenoids isolated from the sponge *Phorbas* sp. activate latent HIV-1 provirus expression | 2016 | Journal of Organic Chemistry |
| Shishicrellastatins, inhibitors of cathepsin B, from the marine sponge *Crella (Yvesia) spinulata* | 2011 | Bioorganic & Medicinal Chemistry |
| Simplexolides A-E and plakorfuran A, six butyrate derived polyketides from the marine sponge *Plakortis simplex* | 2012 | Tetrahedron |
| Simplextones A and B, unusual polyketides from the marine sponge *Plakortis simplex* | 2011 | Organic Letters |
| Single-molecule inhibition of human kinesin by adociasulfate-13 and -14 from the sponge *Cladocroce aculeata* | 2013 | Proceedings of the National Academy of Sciences of the United States of America |
| Sipholane triterpenoids: chemistry, reversal of ABCB1/P-glycoprotein-mediated multidrug resistance, and pharmacophore modeling | 2009 | Journal of Natural Products |
| Six new polyacetylenic alcohols from the marine sponges *Petrosia* sp. and *Halichondria* sp | 2015 | Chemical & Pharmaceutical Bulletin |
| Six trikentrin-like cyclopentanoindoles from *Trikentrion flabelliforme*. absolute structural assignment by NMR and ECD | 2018 | Journal of Organic Chemistry |
| Smenamides A and B, chlorinated peptide/polyketide hybrids containing a dolapyrrolidinone unit from the Caribbean sponge *Smenospongia aurea*. Evaluation of their role as leads in antitumor drug research | 2013 | Marine Drugs |
| Solomonamides A and B, new anti-inflammatory peptides from *Theonella swinhoei* | 2011 | Organic Letters |
| Solomonsterols A and B from *Theonella swinhoei*. The first example of C-24 and C-23 sulfated sterols from a marine source endowed with a PXR agonistic activity | 2011 | Journal of Medicinal Chemistry |
| Solution structure of a sponge-derived cystine knot peptide and its notable stability | 2014 | Journal of Natural Products |
| Spironaamidine, a new spiroquinone-containing alkaloid from the marine sponge *Leucetta microraphis* | 2011 | Tetrahedron Letters |
| Spiroplakortone, an unprecedented spiroketal lactone from the Chinese sponge *Plakortis simplex* | 2015 | RSC Advances |
| Spongiacidin C, a pyrrole alkaloid from the marine sponge *Stylissa massa*, functions as a USP7 inhibitor | 2013 | Bioorganic & Medicinal Chemistry Letters |
| Spongian diterpenes from Chinese marine sponge *Spongia officinalis* | 2018 | Fitoterapia |
| Spongian diterpenes from the sponge *Hyattella aff. intestinalis* | 2017 | Chemical & Pharmaceutical Bulletin |
| Spongiapyridine and related spongians isolated from an Indonesian *Spongia* sp | 2014 | Journal of Natural Products |
| Stable and biocompatible cystine knot peptides from the marine sponge *Asteropus* sp. | 2016 | Bioorganic & Medicinal Chemistry Letters |
| Stellatolide H, a cytotoxic peptide lactone from a deep-sea sponge *Discodermia* sp | 2018 | Tetrahedron Letters |
| Stellatolides, a new cyclodepsipeptide family from the sponge *Ecionemia acervus*: isolation, solid-phase total synthesis, and full structural assignment of stellatolide A | 2014 | Journal of the American Chemical Society |
| Stellettapeptins A and B, HIV-inhibitory cyclic depsipeptides from the marine sponge *Stelletta* sp | 2015 | Tetrahedron Letters |
| Stellettazole D, a cytotoxic imidazole alkaloid from the marine sponge *Jaspis duoaster* | 2011 | Chemistry letters |
| Stelliferins J-N, isomalabaricane-type triterpenoids from Okinawan marine sponge *Rhabdastrella cf. globostellata* | 2011 | Tetrahedron |
| Steroidal alkaloids from the marine sponge *Corticium niger* that inhibit growth of human colon carcinoma cells | 2014 | Journal of Natural Products |
| Steroidal glycosides from the marine sponge *Pandaros acanthifolium* | 2009 | Steroids |
| Steroids and alkaloids from the South China Sea sponge *Axinella* sp | 2009 | Journal of Asian Natural Products Research |
| Steroids from an Australian sponge *Psammoclema* sp | 2009 | Journal of Natural Products |
| Sterols from Thai marine sponge *Petrosia* (*Strongylophora*) sp. and their cytotoxicity | 2017 | Marine Drugs |
| Stimulators of adipogenesis from the marine sponge *Xestospongia testudinaria* | 2013 | Tetrahedron |
| Strongylophorines, meroditerpenoids from the marine sponge *Petrosia corticata*, function as proteasome inhibitors | 2015 | Bioorganic & Medicinal Chemistry Letters |
| Strongylophorines, new protein tyrosine phosphatase 1B inhibitors, from the marine sponge *Strongylophora strongilata* collected at Iriomote Island | 2015 | Bioorganic & Medicinal Chemistry Letters |
| Structural analysis of the minor cerebrosides from a glass sponge *Aulosaccus* sp | 2015 | Lipids |
| Structural and stereochemical investigations into bromotyrosine-derived metabolites from southern Australian marine sponges, *Pseudoceratina* spp | 2012 | Tetrahedron |
| Structural variations to the 9-N-methyladeninium diterpenoid hybrid commonly isolated from *Agelas* sponges | 2010 | Australian Journal of Chemistry |
| Structurally diverse hamigerans from the New Zealand marine sponge *Hamigera tarangaensis*: NMR-directed isolation, structure elucidation and antifungal activity | 2013 | Organic & Biomolecular Chemistry |
| Structure and absolute configuration of 3-alkylpiperidine alkaloids from an Indonesian sponge of the genus *Halichondria* | 2010 | Tetrahedron |
| Structure and stereochemistry of an anti-inflammatory anhydrosugar from the Australian marine sponge *Plakinastrella clathrata* and the synthesis of two analogues | 2013 | Tetrahedron |
| Structure determination of pentacyclic pyridoacridine alkaloids from the Australian marine organisms *Ancorina geodides* and *Cnemidocarpa stolonifera* | 2014 | European Journal of Organic Chemistry |
| Structure elucidation and cytotoxic evaluation of new polyacetylenes from a marine sponge *Petrosia* sp | 2014 | International Journal of Molecular Sciences |
| Structure elucidation at the nanomole scale. 3. phorbasides G-I from *Phorbas* sp | 2010 | Journal of Natural Products |
| Structure elucidation of submilligram quantities of natural products — application to haliclamines G and H from the Arctic marine sponge *Haliclona viscosa* | 2012 | Zeitschrift fur Naturforschung Section B-A Journal of Chemical Sciences |
| Structure elucidation of the new citharoxazole from the Mediterranean deep-sea sponge *Latrunculia* (*Biannulata*) *citharistae* | 2011 | Magnetic Resonance in Chemistry |
| Structure of debromo-carteramine A, a novel bromopyrrole alkaloid from the Mediterranean sponge *Axinella verrucosa* | 2010 | ARKIVOC (Gainesville, FL, U. S.) |
| Structure, synthesis, and biological activity of a C-20 bisacetylenic alcohol from a marine sponge *Callyspongia* sp | 2014 | Journal of Natural Products |
| Structure–activity studies of the pelorusides: new congeners and semi-synthetic analogues | 2011 | Organic & Biomolecular Chemistry |
| Structures and biological evaluations of agelasines isolated from the Okinawan marine sponge *Agelas nakamurai* | 2015 | Journal of Natural Products |
| Structures and cytotoxic evaluation of new and known acyclic ene-ynes from an American Samoa *Petrosia* sp. sponge | 2013 | Journal of Natural Products |
| Structures and mechanisms of antitumor agents: xestoquinones uncouple cellular respiration and disrupt HIF signaling in human breast tumor cells | 2012 | Journal of Natural Products |
| Structures and potential antitumor activity of sesterterpenes from the marine sponge *Hyrtios communis* | 2013 | Journal of Natural Products |
| Structures and solution conformational dynamics of stylissamides G and H from the Bahamian sponge *Stylissa caribica* | 2014 | Journal of Natural Products |
| Structures, semisyntheses, and absolute configurations of the antiplasmodial a-substituted b-lactam monamphilectines B and C from the sponge *Svenzea flava* | 2015 | Tetrahedron |
| Studies on the red sea sponge *Haliclona* sp. for its chemical and cytotoxic properties | 2016 | Pharmacognosy Magazine |
| Stylissamide I, a new cyclic heptapeptide from an Okinawan marine sponge *Stylissa* sp | 2017 | Heterocycles |
| Stylissamide X, a new proline-rich cyclic octapeptide as an inhibitor of cell migration, from an Indonesian marine sponge of *Stylissa* sp | 2012 | Bioorganic & Medicinal Chemistry Letters |
| Stylissamides E and F, cyclic heptapeptides from the Caribbean sponge *Stylissa caribica* | 2010 | Journal of Natural Products |
| Stylissatin A, a cyclic peptide that inhibits nitric oxide production from the marine sponge *Stylissa massa* | 2013 | Tetrahedron Letters |
| Stylissatins B-D, cycloheptapeptides from the marine sponge *Stylissa massa* | 2016 | Tetrahedron Letters |
| Subereamolline A as a potent breast cancer migration, invasion and proliferation inhibitor and bioactive dibrominated alkaloids from the Red Sea sponge *Pseudoceratina arabica* | 2012 | Marine Drugs |
| Suberitane sesterterpenoids from the Antarctic sponge *Phorbas areolatus* (Thiele, 1905) | 2018 | Tetrahedron Letters |
| Suberitine A-D, four new cytotoxic dimeric aaptamine alkaloids from the marine sponge *Aaptos suberitoides* | 2012 | Organic Letters |
| Sulawesins A-C, furanosesterterpene tetronic acids that inhibit USP7, from a *Psammocinia* sp. marine sponge | 2017 | Journal of Natural Products |
| Sulfated steroid-amino acid conjugates from the irish marine sponge *Polymastia boletiformis* | 2015 | Marine Drugs |
| Sulfated steroids: ptilosteroids A-C and ptilosaponosides A and B from the Solomon Islands marine sponge *Ptilocaulis spiculifer* | 2009 | Journal of Natural Products |
| Sulfoureido lipopeptides from the marine sponge *Discodermia kiiensis* | 2016 | Journal of Natural Products |
| Sunabedine, a novel toxic bromotyrosine-derivative alkaloid from okinawan sponge, order Verongida | 2010 | Heterocycles |
| Suvanine analogs from a *Coscinoderma* sp. marine sponge and their cytotoxicities against human cancer cell lines | 2015 | Archives of Pharmacal Research |
| Suvanine sesterterpenes and deacyl irciniasulfonic acids from a Tropical *Coscinoderma* sp. sponge | 2014 | Journal of Natural Products |
| Swinhoeisterols from the South China Sea sponge *Theonella swinhoei* | 2018 | Journal of Natural Products |
| Swinholide J, a potent cytotoxin from the marine sponge *Theonella swinhoei* | 2011 | Marine Drugs |
| Synthesis and absolute conﬁguration of acanthodendrilline, a new cytotoxic bromotyrosine alkaloid from the Thai marine sponge *Acanthodendrilla* sp. | 2016 | Chemical & Pharmaceutical Bulletin |
| Targeted isolation of tsitsikammamines from the Antarctic deep-sea sponge *Latrunculia biformis* by molecular networking and anticancer activity | 2018 | Marine Drugs |
| Tauroarenarones A and B, new taurine-containing meroterpenoids from the marine sponge *Dysidea* sp | 2014 | Natural Product Communications |
| Taurospongins B and C, new acetylenic fatty acid derivatives possessing a taurine amide residue from a marine sponge of the family Spongiidae | 2014 | RSC Advances |
| Tausalarin C: a new bioactive marine sponge-derived nitrogenous bismacrolide | 2009 | Organic Letters |
| Tedanol: A potent anti-inflammatory ent-pimarane diterpene from the Caribbean Sponge *Tedania ignis* | 2009 | Bioorganic & Medicinal Chemistry |
| Tedarenes A and B: structural and stereochemical analysis of two new strained cyclic diarylheptanoids from the marine sponge *Tedania ignis* | 2012 | Journal of Organic Chemistry |
| Terpioside B, a difucosyl GSL from the marine sponge *Terpios* sp. is a potent inhibitor of NO release | 2010 | Bioorganic & Medicinal Chemistry |
| The agminosides: naturally acetylated glycolipids from the New Zealand marine sponge *Raspailia agminata* | 2011 | Journal of Natural Products |
| The aignopsanes, a new class of sesquiterpenes from selected chemotypes of the sponge *Cacospongia mycofijiensis* | 2009 | Organic Letters |
| The halicylindramides, farnesoid X receptor antagonizing depsipeptides from a *Petrosia* sp. marine sponge collected in Korea | 2016 | Journal of Natural Products |
| The marine sponge Agelas citrina as a source of the new pyrrole-imidazole alkaloids citrinamines A-D and N-methylagelongine | 2015 | Beilstein Journal of Organic Chemistry |
| The marine sponge *Diacarnus bismarckensis* as a source of peroxiterpene inhibitors of *Trypanosoma brucei*, the causative agent of sleeping sickness | 2009 | Journal of Natural Products |
| The nitrogenous hamigerans: unusual amino acid-derivatized aromatic diterpenoid metabolites from the New Zealand marine sponge *Hamigera tarangaensis* | 2015 | Journal of Organic Chemistry |
| The oxeatamides: nitrogenous spongian diterpenes from the New Zealand marine sponge *Darwinella oxeata* | 2014 | Journal of Natural Products |
| Theonellamide G, a potent antifungal and cytotoxic bicyclic glycopeptide from the Red Sea marine sponge *Theonella swinhoei* | 2014 | Marine Drugs |
| Theonellasterols and conicasterols from *Theonella swinhoei*. Novel marine natural ligands for human nuclear receptors | 2011 | Journal of Medicinal Chemistry |
| Thiaplakortones A-D: antimalarial thiazine alkaloids from the Australian marine sponge *Plakortis lita* | 2013 | Journal of Organic Chemistry |
| Three bioactive sesquiterpene quinones from the Fijian marine sponge of the genus *Hippospongia* | 2013 | Natural Product Research |
| Three new aaptamine derivatives from the South China Sea sponge *Aaptos aaptos* | 2015 | Journal of Asian Natural Products Research |
| Three new aaptamines from the marine sponge *Aaptos* sp. and their proapoptotic properties | 2010 | Natural Product Communications |
| Three new cytotoxic isomalabaricane triterpenes from the marine sponge *Stelletta tenuis* | 2015 | Fitoterapia |
| Three new non-brominated pyrrole alkaloids from the South China Sea sponge *Agelas nakamurai* | 2017 | Chinese Chemical Letters |
| Three new oxylipins from an Okinawan marine sponge *Plakortis* sp | 2015 | Tetrahedron Letters |
| Three new polyunsaturated lipids from a Guangxi marine sponge *Haliclona* sp | 2015 | Journal of Asian Natural Products Research |
| Three new sesquiterpene aminoquinones from a Vietnamese *Spongia* sp. and their biological activities | 2018 | Journal of Natural Medicines |
| Three new spongian diterpenes from the Fijian marine sponge *Spongia* sp | 2009 | Natural Product Communications |
| Topsendines A-F, new 3-alkylpyridine alkaloids from a Hainan sponge *Topsentia* sp | 2014 | Tetrahedron |
| Topsensterols A-C, cytotoxic polyhydroxylated sterol derivatives from a marine sponge *Topsentia* sp | 2016 | Marine Drugs |
| Topsentinols, 24-isopropyl steroids from the marine sponge *Topsentia* sp | 2010 | Journal of Natural Products |
| Towards new ligands of nuclear receptors. Discovery of malaitasterol A, an unique bis-secosterol from marine sponge *Theonella swinhoei* | 2011 | Organic & Biomolecular Chemistry |
| Trans-dimer D, a novel dimeric sesquiterpene with a bis-bisabolene skeleton from a Hainan sponge *Axinyssa variabilis* | 2011 | Journal of Asian Natural Products Research |
| Treasures from the deep: characellides as anti-inflammatory lipoglycotripeptides from the sponge *Characella pachastrelloides* | 2018 | Organic Letters |
| Tricyclic guanidine alkaloids from the marine sponge *Acanthella cavernosa* that stabilize the tumor suppressor PDCD4 | 2014 | Marine Drugs |
| Trikentramides A-D, indole alkaloids from the Australian sponge *Trikentrion flabelliforme* | 2013 | Journal of Natural Products |
| Trimeric hemibastadin congener from the marine sponge | 2013 | Journal of Natural Products |
| Triterpene galactosides of the pouoside class and corresponding aglycones from the sponge *Lipastrotethya* sp | 2011 | Journal of Natural Products |
| Tulongicin, an antibacterial tri-indole alkaloid from a deep-water *Topsentia* sp. sponge | 2017 | Journal of Natural Products |
| Twilight zone sponges from Guam yield theonellin isocyanate and psammaplysins I and J | 2012 | Journal of Natural Products |
| Two brominated cyclic dipeptides released by the coldwater marine sponge *Geodia barretti* act in synergy as chemical defense | 2011 | Journal of Natural Products |
| Two cell differentiation inducing pyridoacridines from a marine sponge *Biemna* sp. and their chemical conversions | 2015 | Tetrahedron |
| Two furanosesterterpenoids from the sponge *Luffariella variabilis* | 2017 | Marine Drugs |
| Two highly acetylated sterols from the marine sponge *Dysidea* sp | 2017 | Zeitschrift fur Naturforschung Section B-A Journal of Chemical Sciences |
| Two indole-alkaloids from a Korean marine sponge *Spongia* sp | 2015 | Bulletin of the Korean Chemical Society |
| Two isospongian diterpenes from the sponge *Luffariella* sp. | 2017 | Natural Product Communications |
| Two new 5,6-epoxysterols from calcareous marine sponge *Leucetta chagosensis* | 2018 | Natural Product Research |
| Two new alkaloids from marine sponge *Callyspongia* sp | 2013 | Natural Product Research |
| Two new compounds from an Indonesian sponge *Dysidea* sp | 2014 | Journal of Asian Natural Products Research |
| Two new cytotoxic candidaspongiolides from an Indonesian sponge | 2011 | ISRN Pharmaceutics |
| Two new diterpene alkaloids from the South China Sea sponge *Agelas aff. nemoechinata* | 2016 | Chinese Chemical Letters |
| Two new imidazole alkaloids from *Leucetta chagosensis* sponge | 2009 | Saudi Pharmaceutical Journal |
| Two new indole derivatives from a marine sponge *Ircinia* sp. collected at Iriomote Island | 2015 | Journal of Natural Medicines |
| Two new jaspamide derivatives from the marine sponge *Jaspis splendens* | 2009 | Marine Drugs |
| Two new mycosporine-like amino acids LC-343 and mycosporine-ethanolamine from the Micronesian marine sponge *Lendenfeldia chondrodes* | 2017 | Chemistry Letters |
| Two new phthalate derivatives from the marine sponge *Haliclona* sp | 2018 | Chemistry of Natural Compounds |
| Two new polyacetylene derivatives from the Red Sea sponge *Xestospongia* sp | 2015 | Zeitschrift fur Naturforschung Section C-A Journal of Biosciences |
| Two new polyhydroxylated sterol derivatives from the sponge *Topsentia* sp. collected from the South China Sea | 2018 | Chemistry of Natural Compounds |
| Two new protein tyrosine phosphatase 1B inhibitors, hyattellactones A and B, from the Indonesian marine sponge *Hyattella* sp | 2015 | Bioorganic & Medicinal Chemistry Letters |
| Two new pyrrolo-2-aminoimidazoles from a Myanmarese marine sponge, *Clathria prolifera* | 2018 | Journal of Natural Medicines |
| Two new scalaranes from a Korean marine Sponge *Spongia* sp | 2015 | Natural Product Sciences |
| Two new scaralane-type sesterterpenoids isolated from the marine sponge *Hyrtios erectus* | 2014 | Records of Natural Products |
| Two new spongian diterpene analogues isolated from the marine sponge *Acanthodendrilla* sp | 2018 | Natural Product Research |
| Two new steroids with cytotoxicity from the marine sponge *Dactylospongia elegans* collected from the South China Sea | 2018 | Natural Product Research |
| Two new thyminenol derivatives from the marine sponge *Haliclona* sp | 2014 | Natural Product Communications |
| Two new unprecedented acetonyl-bearing sesquiterpenes from the Hainan sponge *Dysidea fragilis* | 2009 | Chemistry & Biodiversity |
| Two novel alkaloids from the South China Sea marine sponge *Dysidea* sp | 2010 | The Journal of Antibiotics |
| Two rare-class tricyclic diterpenes with antitubercular activity from the Caribbean sponge *Svenzea flava*. application of vibrational circular dichroism spectroscopy for determining absolute configuration | 2013 | Journal of Organic Chemistry |
| Two-dimensional ultra high pressure liquid chromatography quadrupole/time-of-flight mass spectrometry for semi-targeted natural compounds identification | 2014 | Phytochemistry Letters |
| Tyrokeradines A and B, new bromotyrosine alkaloids with an imidazolyl-quinolinone moiety from a Verongid sponge | 2009 | Bioorganic & Medicinal Chemistry Letters |
| Tyrokeradines C-F, new bromotyrosine alkaloids from the verongid sponges | 2012 | Chemical & Pharmaceutical Bulletin |
| Tyrokeradines G and H, new bromotyrosine alkaloids from an Okinawan Verongid sponge | 2015 | Bioorganic & Medicinal Chemistry Letters |
| Ulososides and urabosides - triterpenoid saponins from the Caribbean marine sponge *Ectyoplasia ferox* | 2013 | Molecules |
| Unguiculin A and ptilomycalins E-H, antimalarial guanidine alkaloids from the marine sponge *Monanchora unguiculata* | 2017 | Journal of Natural Products |
| Unguiculins A-C: cytotoxic bis-guanidine alkaloids from the French Polynesian sponge, *Monanchora* n. sp | 2018 | Natural Product Research |
| Unprecedented stylissazoles A-C from *Stylissa carteri*: another dimension for marine pyrrole-2-aminoimidazole metabolite diversity | 2010 | Angewandte Chemie-International Editio |
| Untenolide A, a new polyketide from an Okinawan marine sponge *Plakortis* sp | 2010 | Tetrahedron Letters |
| Unusual anti-allergic diterpenoids from the marine sponge *Hippospongia lachne* | 2017 | Scientific Reports |
| Unusual anti-inflammatory meroterpenoids from the marine sponge *Dactylospongia* sp. | 2018 | Organic & Biomolecular Chemistry |
| Urupocidin A: a new, inducing iNOS expression bicyclic guanidine alkaloid from the marine sponge *Monanchora pulchra* | 2014 | Organic Letters |
| Using enzyme assays to evaluate the structure and bioactivity of sponge-derived meroterpenes | 2009 | Journal of Natural Products |
| Viscosalines B1,2 and E1,2: challenging new 3-alkyl pyridinium alkaloids from the marine sponge *Haliclona viscosa* | 2012 | Chemistry-A European Journal |
| Woodylides A-C, new cytotoxic linear polyketides from the South China Sea sponge *Plakortis simplex* | 2012 | Marine Drugs |
| Xestoproxamines A-C from *Neopetrosia proxima*. assignment of absolute stereostructure of bis-piperidine alkaloids by integrated degradation-cd analysis | 2011 | Journal of Natural Products |
| Xestosaprol D and E from the Indonesian marine sponge *Xestospongia* sp | 2010 | Tetrahedron Letters |
| Xestosaprols from the Indonesian marine sponge *Xestospongia* sp | 2010 | Journal of Natural Products |
| Xestospongienols A-L, brominated acetylenic acids from the Chinese marine sponge *Xestospongia testudinaria* | 2011 | Helvetica Chimica acta |
| Yaku'amides A and B, cytotoxic linear peptides rich in dehydroamino acids from the marine sponge *Ceratopsion* sp | 2010 | Journal of the American Chemical Society |
| Yakushinamides, polyoxygenated fatty acid amides that inhibit HDACs and SIRTs, from the marine sponge *Theonella swinhoei* | 2016 | Journal of Natural Products |
| Zamamidine C, 3,4-dihydro-6-hydroxy-10,11-epoxymanzamine A, and 3,4-dihydromanzamine J N-oxide, new manzamine alkaloids from sponge *Amphimedon* sp | 2009 | Tetrahedron |
| Zamamidine D, a manzamine alkaloid from an Okinawan *Amphimedon* sp. marine sponge | 2017 | Journal of Natural Products |
| Zamamiphidin A, a new manzamine related alkaloid from an Okinawan marine sponge *Amphimedon* sp | 2013 | Organic Letters |
| Zampanolides B-E from the marine sponge *Cacospongia mycofijiensis*: potent cytotoxic macrolides with microtubule-stabilizing activity | 2018 | Journal of Natural Products |
| Zeamide, a glycosylinositol phosphorylceramide with the novel core aarap(1β→6)ins motif from the marine sponge *Svenzea zeai* | 2017 | Molecules |
